# The EU(7)-PIM list: a list of potentially inappropriate medications for older people consented by experts from seven European countries

**DOI:** 10.1007/s00228-015-1860-9

**Published:** 2015-05-14

**Authors:** Anna Renom-Guiteras, Gabriele Meyer, Petra A. Thürmann

**Affiliations:** Institute of General Practice and Family Medicine, Faculty of Health, University of Witten/Herdecke, Alfred-Herrhausen-Straße 50, 58448 Witten, Germany; Universitat Autònoma de Barcelona, Barcelona, Spain; Institute for Health and Nursing Science, Medical Faculty, Martin Luther University Halle-Wittenberg, Magdeburger Straße 8, 06112 Halle (Saale), Germany; School of Nursing Science, Faculty of Health, University of Witten/Herdecke, Witten, Germany; Clinical Pharmacology, Faculty of Health, University of Witten/Herdecke, Witten, Germany; Philipp Klee-Institute of Clinical Pharmacology, HELIOS Klinikum Wuppertal, Heusnerstraße 40, 42283 Wuppertal, Germany

**Keywords:** Potentially inappropriate medication, Inappropriate prescribing [MeSH term], Aged [MeSH term], Screening, Europe [MeSH term]

## Abstract

**Purpose:**

The aim of the study was to develop a European list of potentially inappropriate medications (PIM) for older people, which can be used for the analysis and comparison of prescribing patterns across European countries and for clinical practice.

**Methods:**

A preliminary PIM list was developed, based on the German PRISCUS list of potentially inappropriate medications and other PIM lists from the USA, Canada and France. Thirty experts on geriatric prescribing from Estonia, Finland, France, the Netherlands, Spain and Sweden participated; eight experts performed a structured expansion of the list, suggesting further medications; twenty-seven experts participated in a two-round Delphi survey assessing the appropriateness of drugs and suggesting dose adjustments and therapeutic alternatives. Finally, twelve experts completed a brief final survey to decide upon issues requiring further consensus.

**Results:**

Experts reached a consensus that 282 chemical substances or drug classes from 34 therapeutic groups are PIM for older people; some PIM are restricted to a certain dose or duration of use. The PIM list contains suggestions for dose adjustments and therapeutic alternatives.

**Conclusions:**

The European Union (EU)(7)-PIM list is a screening tool, developed with participation of experts from seven European countries, that allows identification and comparison of PIM prescribing patterns for older people across European countries. It can also be used as a guide in clinical practice, although it does not substitute the decision-making process of individualised prescribing for older people. Further research is needed to investigate the feasibility and applicability and, finally, the clinical benefits of the newly developed list.

**Electronic supplementary material:**

The online version of this article (doi:10.1007/s00228-015-1860-9) contains supplementary material, which is available to authorized users.

## Background

Appropriate prescribing for older people is a public health concern, and several assessment tools are available for its evaluation. Most of the tools focus on pharmacological appropriateness of prescribing [1]; they address various aspects of appropriateness, including overprescribing of medications that are clinically not indicated, omission of medications that are needed, and incorrect prescriptions of medications that may be indicated [2]. The term “potentially inappropriate medications (PIM) for older people” has been used to refer to those drugs which should not be prescribed for this population because the risk of adverse events outweighs the clinical benefit, particularly when there is evidence in favour of a safer or more effective alternative therapy for the same condition [3, 4].

The prevalence of inappropriate prescribing and/or use of PIM has been analysed by several authors and ranges from 20 to 79 % depending on the population studied, the setting or country, and the specific tool used [5–10]. Inappropriate prescribing and use of PIM can be associated with adverse outcomes such as adverse drug events [11–13], hospitalisation [6, 14] and death [15].

A recently published systematic review identified 46 tools or criteria for assessing inappropriate prescribing [16]. A prior systematic review identified 14 criteria specific for individuals aged 65 and older [1]. Generally, the assessment tools have been developed based on expert opinion due to the lack of high-quality studies on the use of drugs in older people [17], although some tools have additionally used a literature search [18, 19]. Criteria have been classified into explicit or implicit or mixed approach [1]. Explicit criteria are generally lists of medications or criteria which can be applied with little or no clinical judgement but do not address individual differences between patients [2]. Implicit criteria are based on the judgement of a professional and are person-specific [20], requiring individual patient data for application, however, they are time-consuming and more dependent on the user [2]. No single ideal tool has been identified so far, but each tool seems to have its strengths and weaknesses, and the choice of a tool may depend on the purpose of use (i.e. daily practice, research) and availability of data [16].

Assessment tools are being used increasingly for the evaluation of prescribing quality in older people, but their application cannot substitute the individual assessment of prescribing appropriateness [16]. One of the limitations of the tools is the fact that the majority was developed following country-specific guidelines, national drug markets and prescribing habits, hence, limiting their transferability to other countries [1, 21]. For instance, the German PRISCUS list of potentially inappropriate medications, a purely explicit list, defines 83 PIM drugs, of which twelve are not on the drug market in France, the USA and Canada. However, there are 124 drugs on the PIM lists of these countries which are not part of the German PRISCUS list, because seventy of them are not on the German drug market and many others are almost never used [22]. To the best of our knowledge, no assessment tool covers the drug markets of several European countries and could thus enable the analysis of European databases.

The present study was conceived when planning to analyse the prescription of PIM among a European cohort of older people with dementia participating in the *RightTimePlaceCare* study [23]. The primary aim of our study was to develop an expert-consensus list of potentially inappropriate medications covering the drug markets of seven European countries, which can be used for the analysis of potentially inappropriate prescription patterns in and across several European countries. Additionally, the list should be applicable in clinical practice to alert health care professionals to the likelihood of inappropriate prescribing, possible dose adjustments required and therapeutic alternatives.

## Methods

A research team consisting of a clinical pharmacologist, a pharmacist, a nursing scientist and a geriatrician planned and coordinated the development of the European Union (EU)(7)-PIM list. Two members of the research team were developers of the German PRISCUS list [22]. The study comprised five consecutive phases:

1. *Preparation of a preliminary PIM list*. We prepared a preliminary PIM list which contained 85 PIM (82 active substances plus one combination of active substances and two different preparations of one substance) from the German PRISCUS list [22] and 99 PIM from the French [3], American [24, 25] and Canadian [26] lists. These tools have been used in research to evaluate the prescription of PIM and factors associated with PIM use [5, 6, 14, 27–29]. The main reason for each drug being PIM was formulated using the information provided by the original lists. This process was supported by a comprehensive literature search. The anatomical therapeutic chemical (ATC) code classification system was used (2011) [30].
2. *Recruitment of experts on geriatric prescribing*/*pharmacotherapy*. We established a collaboration with the Seventh Framework European project *RightTimePlaceCare* [23], a project aiming to develop best practice recommendations for dementia care throughout Europe. The consortium partners of this project supported the recruitment of experts on geriatric prescribing or pharmacotherapy in their respective countries. Thirty-three experts from six European countries agreed to participate; they came from Finland (*n* = 3), Estonia (*n* = 9), the Netherlands (*n* = 4), France (*n* = 2), Spain (*n* = 7) and Sweden (*n* = 8). The following professions were represented as follows: geriatricians (*n* = 14), pharmacists (*n* = 3), clinical pharmacologists (*n* = 7) and other medical specialists (*n* = 9). Experts were sent information documents describing the aims, concepts and steps of the study and were asked whether they preferred to participate in the expansion phase (phase 3), in the Delphi survey (phase 4), or in both.
3. *Expansion of the preliminary PIM list*. We asked thirteen experts representing the six countries to expand the preliminary PIM list by adding drugs that they considered should be PIM and which were not represented, paying special attention to those drugs available on their respective countries’ markets. Expansion of the preliminary list was Internet-based and concluded in May 2012.
4. *Two-round Delphi survey*. A two-round Delphi survey was performed [31]. The first Delphi round took place between October and December 2012, and the second Delphi round between March and May 2013. In the first round, we asked 29 experts to assess each drug of the preliminary expanded list for appropriateness by using a 1–5 points Likert scale where “1” represented “I strongly agree that the drug is potentially inappropriate for older people”; “2”, “I agree that the drug is potentially inappropriate for older people”; “3”, “average/neutral/undecided”; “4”, “I disagree that the drug is potentially inappropriate for older people”; “5”, “I strongly disagree that the drug is potentially inappropriate for older people”; and “0”, “no answer; I do not feel qualified to answer”. Experts were asked to provide suggestions for dose adjustments and safer therapeutic alternatives for those drugs judged as inappropriate. Experts were free to insert additional comments and were invited to expand the list with any further drugs they considered to be PIM.
  In the second Delphi round, we asked 28 experts to assess the appropriateness of those drugs classified as questionable PIM during the first round (see “Expert agreement and statistics”), as well as the further suggestions for PIM made by the experts during the first Delphi round, and also eight drugs appearing in the recently published updated Beers list [18]. Some PIM concepts were adapted taking the experts’ suggestions made during the first Delphi round into account. The additional suggestions for PIM were given a justification as to why they may be classified as PIM, taking published data into consideration when necessary. Again, experts assessed the appropriateness of these drugs and were asked to provide dose adjustments, therapeutic alternatives, and to insert additional comments if necessary. Drugs were classified into PIM, non-PIM and questionable PIM (see “Expert agreement and statistics”).
5. *Preparation of the final PIM list*. Dose adjustments and drug alternatives suggested by the experts during the Delphi survey were compiled and included in the EU(7)-PIM list, prioritising in each case those made by the higher number of experts. Suggestions were complemented, if necessary, with information available from the other PIM lists and from Micromedex® [32], a commercially available database which contains comprehensive information on drug use. We identified those drugs for which some discussion issues raised by the experts still remained open and those drugs where inconsistency in the results was identified after checking the literature. In order to solve these problems, a reduced number of experts (*n* = 12) was invited to participate in the last brief survey which took place in September 2013.

### Expert agreement and statistics

Several approaches have been suggested in the literature to define expert agreement within Delphi surveys [31]. In this study, after the first and second Delphi rounds, we calculated the means, the corresponding 95 % confidence intervals (CI) and the medians of all Likert scores given to each drug; expert agreement was considered if the CI of the mean score for each drug did not cross over the value 3. Thus, each drug was classified into PIM (if both the mean value of the score and the upper limit of the CI were lower than 3), non-PIM (if both the mean value of the score and the lower limit of the CI exceeded 3) and questionable PIM (if the CI was on both sides of the value 3). Statistical calculations were performed with SPSS, version 21.0.

## Results

The preliminary PIM list contained 184 drugs (including two combinations of two drugs) and preparations (e.g. sustained-release preparations of oxybutynine). Eight of the 13 invited experts (62 %) participated in the expansion phase and suggested 75 additional drugs and preparations. Twenty-six out of the 29 invited experts (90 %) participated in the first Delphi round, and 24 out of the 28 invited experts (86 %) participated in the second Delphi round. Two experts from Spain and three experts from Finland chose to collaborate together in two teams to provide their assessments in both Delphi rounds. All the 12 experts invited participated in the last brief survey.

**Fig. 1:**
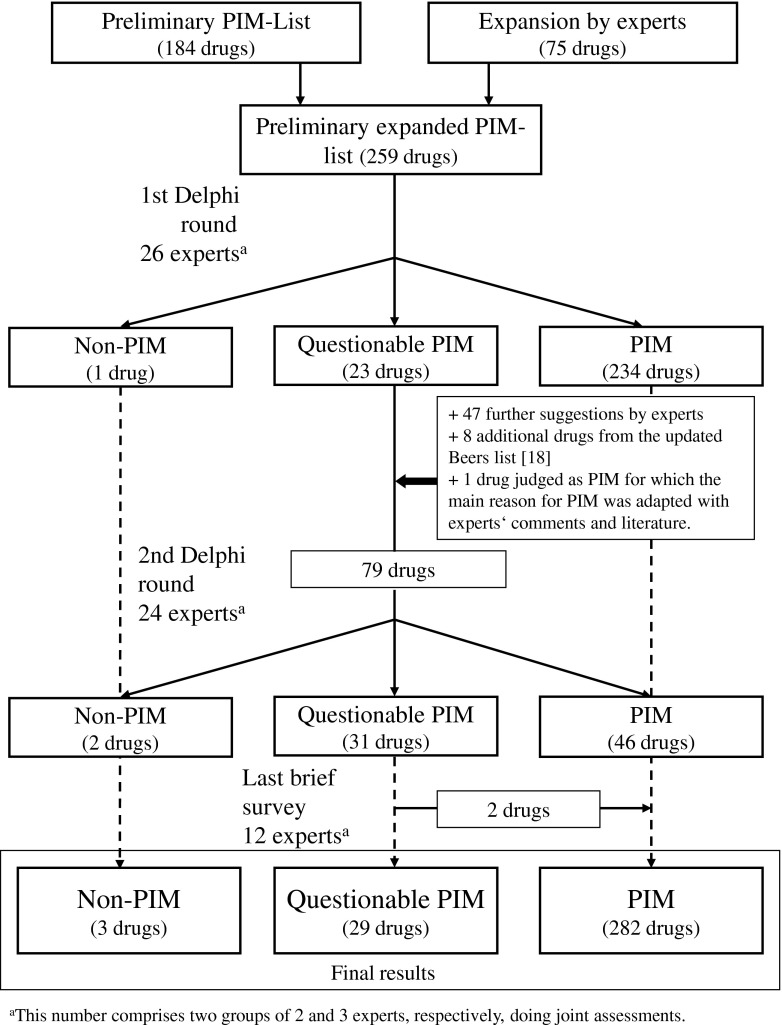
The development process of the EU(7)-PIM list

Figure 1 shows the development process of the list. In the first Delphi round, experts assessed 259 drugs and preparations, of which the majority (*n* = 234) were classified as PIM and only one drug as non-PIM. In the second Delphi round, experts assessed 79 drugs and preparations, comprising 23 questionable PIM, 47 further suggestions by experts, eight additional drugs from the updated Beers list [18] and one drug (naproxen) judged as PIM for which the main reason for PIM was adapted taking recent published data and experts’ comments into consideration. Again, 31 drugs and preparations remained as questionable PIM and 46 drugs were classified as PIM. Overall, after the third brief survey, 282 drugs and preparations were classified as PIM, 29 as questionable PIM and three as non-PIM.

The level of agreement between experts varied in the assessment of appropriateness. For example, experts reached consensus for diazepam being PIM with a mean Likert score of 1.61, confidence interval between 1.32 and 1.89, and median of 2. Consensus was reached also for digoxin being PIM (mean Likert score 2.19; confidence interval 1.57–2.81; median 2), but in this case, the Likert scores ranged from 1 to 5. No consensus was reached on the appropriateness of some drugs such as metamizole, which was classified as questionable PIM. For this drug, the disparity seemed to be in part due to the experts’ country of origin, since the majority of the Spanish experts considered metamizole to be appropriate when used in adequate doses, whereas the majority of Finnish experts considered this drug to be clearly inappropriate.

The last brief survey consisted of 11 questions with multiple-choice answers and covered issues regarding 13 drugs. The questions covered mostly dose-related issues commented by the experts during the survey which remained open (four drugs) and inconsistencies in the results identified after checking the literature (three drugs). Additionally, the research group asked the experts to provide their opinion on the use of three drugs. Finally, the research group did minimal corrections in the PIM which needed experts’ approval (three drugs). All of the issues could be solved.

Table 1 displays an abbreviated version of the EU(7)-PIM list, with the 72 PIM most frequently identified among the participants of the *RightTimePlaceCare* survey [23], a European cohort of older people with dementia (data not shown).

**Table 1:** PIM according to the EU(7)-PIM list^a^

PIM	Main reason	Dose adjustment/special considerations of use	Alternative drugs and/or therapies
Drugs for peptic ulcer and gastro-oesophageal reflux			
Ranitidine	CNS adverse effects including confusion	CrCl <50 mL/min 150 mg q 24h (oral); 50 mg q 18–24 h (iv). E	When indication is appropriate, PPI (<8 weeks, low dose). E
PPI (>8 weeks) e.g. omeprazole, pantoprazole	Long-term high dose PPI therapy is associated with an increased risk of *C. difficile* infection and hip fracture. Inappropriate if used >8 weeks in maximal dose without clear indication		
Propulsives			
Metoclopramide	Antidopaminergic and anticholinergic effects, may worsen peripheral arterial blood flow and precipitate intermittent claudication	Short-term use and dose reduction; CrCl <40 mL/min 50 % of normal dose; maximum dose 20 mg/d; may be used in palliative care. E	Domperidone (<30 mg/d) if no contraindications. E
Laxatives			
Senna glycosides	Stimulant laxative. Adverse events include abdominal pain, fluid and electrolyte imbalance and hypoalbuminemia. May exacerbate bowel dysfunction		Recommend proper dietary fibre and fluid intake; osmotically active laxatives: macrogol, lactulose. E, P
Sodium picosulfate			
Antipropulsives			
Loperamide (>2 days)	Risk of somnolence, constipation, nausea, abdominal pain and bloating. Rare adverse events include dizziness. May precipitate toxic megacolon in inflammatory bowel disease, may delay recovery in unrecognised gastroenteritis	Start with a dose of 4 mg followed by 2 mg in each deposition until normalisation of bowel; do not exceed 16 mg/d; use no longer than 2 days; may be useful in palliative care for persisting non-infectious diarrhoea. E	Non-pharmacological measures, e.g. diet; phloroglucinol. E
Insulins and analogues			
Insulin, sliding scale	No benefits demonstrated in using sliding-scale insulin. Might facilitate fluctuations in glycemic levels	Lower doses to avoid hypoglycemia. E	Basal insulin. E
Blood glucose lowering drugs, excluding insulins			
Glibenclamide	Risk of protracted hypoglycemia	Use conservative initial dose (1.25 mg/d for non-micronized glibenclamide; 0.75 mg/d for micronized glibenclamide) and maintenance dose; not recommended if CrCl <50 mL/min. M	Diet; metformin (<2 × 850 mg/d); insulin; gliclazide may be safer than the other short-acting sulphonilureas. E
Glimepiride	Risk of protracted hypoglycemia	Adjust according to renal function. E For patients with renal failure and for older people, use initial dose of 1 mg/d followed by a conservative titration scheme. Titrate dose in increments of 1 to 2 mg no more than every 1 to 2 weeks based on individual response. M	
Sitagliptine	Limited safety data available for adults aged ≥75 years old. Subjects aged 65 to 80 had higher plasma concentrations than younger subjects. Risk of hypoglycemia, dizziness, headache and peripheral oedema	Reduce dose to 50 mg/d in cases of renal failure (CrCl 30–50 mL/min); reduce dose to 25 mg/d in cases of severe renal insufficiency (CrCl <30 mL/min). E, M	
Antithrombotic agents			
Acenocoumarol	Risk of bleeding, especially in people with difficult control of INR value		
Dipyridamole	Less efficient than aspirin; risk of vasodilatation and orthostatic hypotension. Proven beneficial only for patients with artificial heart valves		Clopidogrel; aspirin (<325 mg)^b^. E, L
Iron preparations			
Iron supplements / Ferrous sulfate (>325 mg/d)	Doses >325 mg/d do not considerably increase the amount absorbed but greatly increase the incidence of constipation		Intravenous iron. E
Cardiovascular system			
Cardiac glycosides			
Digitoxin	Elevated glycoside sensitivity in older people (women > men); risk of intoxication	Calculate digitalizing doses based on lean body mass and maintenance doses using actual CrCl. M	For tachycardia/atrial fibrillation: beta-blockers (except oxprenolol, pindolol, propranolol, sotalol, nadolol, labetalol). E, P For congestive heart failure: diuretics (except spironolactone >25 mg/d), ACE inhibitors. E
Digoxin		Calculate digitalizing doses based on lean body mass and maintenance doses using actual CrCl. M For older people, use dose 0.0625–0.125 mcg/d; in cases of renal failure (CrCl 10–50 mL/min), administer 25–75 % of dose or every 36 h; in cases of renal failure (CrCl <10 ml/min), administer 10–25 % of dose or every 48 h. E	
Antiarrhythmics, classes I and III			
Amiodarone	Associated with QT interval problems and risk of provoking torsades de pointes	Start dose at the low end of the dosing range. M Use lower maintenance dose, e.g. 200 mg/48 h. E	Data suggest that for most older people rate control yields better balance of benefits and harms than rhythm control for most of older people. B
Other cardiac preparations			
Trimetazidine	Can cause or worsen parkinsonian symptoms (tremor, akinesia, hyperthonia); caution in cases of moderate renal failure and with older people (>75 years old); efficacy for the treatment of tinnitus or dizziness not proven	20 mg twice per day for patients with moderate renal insufficiency. E	
Antiadrenergic agents, centrally acting			
Rilmenidine	Risk of orthostatic hypotension, bradycardia, syncope, CNS side effects (sedation, depression, cognitive impairment)	Reduce dose in cases of renal failure (CrCl <15 mL/min). M, E	Other antihypertensive drugs, e.g. ACE inhibitors, or other medication groups depending on comorbidity (exclude PIM). E
Antiadrenergic agents, peripherally acting			
Doxazosin	Higher risk of orthostatic hypotension, dry mouth, urinary incontinence/ impaired micturition, CNS side effects (e.g. vertigo, light-headedness, somnolence) and cerebrovascular and cardiovascular disease	Start with half of usual dose, taper in and out. P Start with 0.5 mg/d (immediate release) or 4–8 mg/d (extended release). E	Other antihypertensive drugs, e.g. ACE inhibitors, or other medication groups depending on comorbidity (exclude PIM). E
Potassium-sparing agent			
Spironolactone (>25 mg/d)	Higher risk of hyperkalaemia and hyponatremia in older people, especially if doses >25 mg/d, requiring periodic controls	Reduce dose in cases of moderate renal insufficiency. E, M GFR ≥50 mL/min/1.73 m: initial dose 12.5–25 mg/d, increase up to 25 mg 1–2/d; GFR 30–49 mL/min/1.73 m: initial dose 12.5 mg/d, increase up to 12.5–25 mg/d; reduce dose if potassium levels increase or renal function worsens. GFR <10 mL/min: avoid. M	Consider alternatives depending on the indication; exclude PIMs
Peripheral vasodilators			
Pentoxifylline	No proven efficacy; unfavourable risk/benefit profile; orthostatic hypotension and fall risks are increased with most vasodilators	Reduce dose to 400 mg twice daily in cases of moderate renal failure and to 400 mg once daily in cases of severe renal failure; close monitoring for toxicities. Avoid use if CrCl <30 mL/min. M	
Beta blocking agents			
Propranolol	Non-selective beta-adrenergic blocker; may exacerbate or cause respiratory depression; possible CNS adverse events	3 doses of 20 mg daily E start low—go slow for older people and patients with renal failure. M	Depending on the indication: cardioselective beta-blockers, ACE inhibitors, diuretics. E
Sotalol		Start at half or one third of the typical dose and increase slowly. P Reduce dose and dosing interval in cases of renal failure. M	Cardioselective beta-blockers (e.g. metoprolol, bisoprolol, carvedilol, atenolol). E
Selective calcium channel blockers with mainly vascular effects			
Nifedipine (non-sustained-release)	Increased risk of hypotension; myocardial infarction; increased mortality	Lower initial dose, half of usual dose, taper in and out. P	Other antihypertensive drugs (amlodipine, cardioselective beta-blockers, ACE inhibitors, diuretics). E, L
Nifedipine (sustained-release)		Lower initial dose, half of usual dose, taper in and out. P Initial dose 30 mg/d; maintenance dose 30–60 mg/d. E	
Selective calcium channel blockers with direct cardiac effects			
Verapamil	May worsen constipation; risk of bradycardia	Immediate-release tablets: initial dose 40 mg three times daily; sustained release tablets initial dose 120 mg daily; oral controlled onset extended release initial dose 100 mg/d. M	Other antihypertensive drugs (amlodipine, cardioselective beta-blockers, ACE inhibitors, diuretics). E
Diltiazem		Reduce dose or increase dosing interval. M 60 mg three times daily. E	
Oestrogens			
Oestrogen	Evidence for carcinogenic potential (breast and endometrial cancer) and lack of cardioprotective effect in older women		Specific treatment for osteoporosis. E Local administration (i.e. vaginal application) considered safe and efficient. E, B
Other urologicals, including antispasmodics			
Oxybutynine (non-sustained-release)	Anticholinergic side effects (e.g. constipation, dry mouth, CNS side effects); ECG changes (prolonged QT)	Start immediate-release oxybutynin chloride in frail older people with 2.5 mg orally 2 or 3 times daily. M	Non-pharmacological treatment (pelvic floor exercises, physical and behavioural therapy). E
Oxybutynine (sustained-release)			
Tolterodine (non-sustained-release)		1 mg orally twice daily in cases of significantly impaired renal function. M	
Tolterodine (sustained-release)		Use 2 mg orally once daily in cases of severe renal failure (CrCl 10–30 mL/min); avoid use if CrCl <10 mL/min. M	
Solifenacin		Dose reduction may be needed. M	
Anti-inflammatory and antirheumatic products, non-steroid (NSAID)			
Diclofenac	Very high risk of GI bleeding, ulceration, or perforation, which may be fatal; cardiovascular contraindications	50 mg/d; start using low dose; the risk of bleeding may be reduced if combined with proton-pump inhibitors (use <8 weeks, low dose). E	Paracetamol; ibuprofen (≤3 × 400 mg/d or for a period shorter than one week); naproxen (≤2 × 250 mg/d or for a period shorter than one week). E Opiods with lower risk of delirium (e.g. tilidine/naloxone, morphine^b^, oxycodone, buprenorphine, hydromorphone). E, P
Dexketoprofen		Start with lower dose, up to 50 mg/d in older people; in postoperative pain: 50 mg/d in case of renal or hepatic failure, maximum dose 50 mg/8 h; maximum length 48 h; the risk of bleeding may be reduced if combined with proton-pump inhibitors (use <8 weeks, low dose). E	
Etoricoxib		Shortest possible duration of therapy. P Start with lower dose; the risk of bleeding may be reduced if combined with proton-pump inhibitors (use <8 weeks, low dose). E	
Meloxicam	Very high risk of GI bleeding, ulceration, or perforation, which may be fatal	11 mg/d; start with lower dose; the risk of bleeding may be reduced if combined with proton-pump inhibitors (use <8 weeks, low dose). E	
Ibuprofen (>3 × 400 mg/d or for a period longer than one week)	Risk of GI bleeding and increased risk of cardiovascular complications at higher doses (>1200 mg/d), especially in case of previous cardiovascular disease	The risk of bleeding may be reduced if combined with proton-pump inhibitors (use <8 weeks, low dose). E	
Drugs affecting bone structure and mineralization			
Strontium ranelate	Higher risk of venous thromboembolism in persons who are temporarily or permanently immobilised. Evaluate the need for continued therapy for patients over 80 years old with increased risk of venous thromboembolism	Avoid in cases of severe renal failure (CrCl <30 mL/min). M	Bisphosphonates, vitamin D. E
Opioids			
Tramadol (sustained-release)	More adverse effects in older people; CNS side effects such as confusion, vertigo and nausea	Start low—go slow. Not to be used in cases of severe renal failure. E, M	Paracetamol; ibuprofen (≤3 × 400 mg/d or for a period shorter than one week); naproxen (≤2 × 250 mg/d or for a period shorter than one week). E Opioids with lower risk of delirium (e.g. tilidine/naloxone, morphine^b^, oxycodone, buprenorphine, hydromorphone). E, P
Tramadol (non-sustained-release)		Start low—go slow; in persons older than 75 years, daily doses over 300 mg are not recommended. M Start with 12.5 mg/8 h and progressive increases of 12.5 mg/8 h; maximum 100 mg/8 h. E Reduce dose and extend the dosing interval for patients with severe renal failure. M	
Antiepileptics			
Clonazepam	Risk of falls, paradoxical reactions.	Start low—go slow; 0.5 mg/day. E	Levetiracetam^b^; gabapentin^b^; lamotrigine^b^; valproic acid^b^. E
Carbamazepine	Increased risk of SIADH-like syndrome; adverse events like carbamazepine-induced confusion and agitation, atrioventricular block and bradycardia	Adjust dose to the response and serum concentration. E	
Dopaminergic agents			
Ropinirole	Risk of orthostatic hypotension, hallucinations, confusion, somnolence, nausea	Start with three intakes of 0.25 mg per day, increase gradually by 0.25 mg per intake each week for four weeks, up to 3 mg/d. Afterwards the dose may be increased weekly by 1.5 mg/d up to 24 mg/d. E	Levodopa; carbidopa-levodopa; benserazide levodopa; irreversible inhibitor of monoamine oxidase as rasagiline. E
Pramipexole	Side effects include orthostatic hypotension, GI tract symptoms, hallucinations, confusion, insomnia, peripheral oedema	Reduce dose in cases of moderate to severe renal failure. M Start with three intakes of 0.125 per day, increase gradually by 0.125 mg per intake every five to seven days, up to 1.5 to 4.5 mg. E	
Antipsychotics			
Chlorpromazine	Muscarinic-blocking drug; risk of orthostatic hypotension and falls; may lower seizure thresholds in patients with seizures or epilepsy	Start low—go slow; use one third to one half the normal adult dose for debilitated older people; use maintenance doses of 300 mg or less; doses greater than 1 g do not usually offer any benefit, but may be responsible for an increased incidence of adverse effects. M	Non-pharmacological treatment; risperidone (<6 weeks), olanzapine (<10 mg/d), haloperidol (<2 mg single dose; < 5 mg/d); quetiapine^b^. E
Levomepromazine	Anticholinergic and extrapyramidal side effects (tardive dyskinesia); parkinsonism; hypotonia; sedation; risk of falling; increased mortality in persons with dementia	Administer cautiously in cases of renal failure; start with doses of 5 to 10 mg in geriatric patients. M	
Haloperidol (>2 mg single dose; >5 mg/d)		Use oral doses of 0.75-1.5 mg; use for the shortest period possible. E	
Zuclopenthixol	Risk of hypotension, falls, extrapyramidal effects, QTc-prolongation	Use low oral doses of 2.5–5 mg/d. M	
Clozapine	Anticholinergic and extrapyramidal side effects (tardive dyskinesia); parkinsonism; hypotonia; sedation; risk of falling; increased mortality in persons with dementia; increased risk of agranulocytosis and myocarditis	Start with 12.5 mg/d. E Start low—go slow; reduce dose in cases of significant renal failure. M	
Risperidone (>6 weeks)	Problematic risk-benefit profile for the treatment of behavioural symptoms of dementia; increased mortality, with higher dose, in patients with dementia	Use the lowest dose required (0.5–1.5 mg/d) for the shortest time period necessary. E For geriatric patients or in cases of severe renal failure (CrCl <30 mL/min), start with 0.5 mg twice daily; increase doses by 0.5 mg twice daily; increases above 1.5 mg twice daily should be done at intervals of at least 1 week; slower titration may be necessary. For geriatric patients, if once-daily dosing desired, initiate and titrate on a twice-daily regimen for 2 to 3 days to achieve target dose and switch to once-daily dosing thereafter. M	
Anxiolytics			
Diazepam	Risk of falling with hip fracture; prolonged reaction times; psychiatric reactions (can also be paradoxical, e.g. agitation, irritability, hallucinations, psychosis); cognitive impairment; depression	Use the lowest possible dose, up to half of the usual dose, taper in and out, shortest possible duration of treatment. P, M Use initial oral dose of 2–2.5 mg once a day to twice a day. M	Non-pharmacological treatment; low doses of short-acting benzodiazepines such as lormetazepam (≤0.5 mg/d), brotizolam (≤0.125 mg/d); antidepressants with anxiolytic profile (SSRI^c^). E, P If used as hypnotics or sedatives: see alternatives proposed for “hypnotics and sedatives”
Lorazepam (>1 mg/d)		Reduce dose; use doses of 0.25–1 mg/d. E	
Bromazepam		Use the lowest possible dose, up to half of the usual dose, taper in and out according to individual response, shortest possible duration of treatment. P, M	
Alprazolam		Use the lowest possible dose, up to half of the usual dose, taper in and out, shortest possible duration of treatment. P Starting dose 0.25 mg/12 h. E Immediate release tablets (including orally disintegrating tablets): start with 0.25 mg administered two to three times a day and titrate as tolerated; extended-release tablets: start with 0.5 mg once daily, gradually increase as needed and tolerated. M	
Hypnotics and sedatives			
Flunitrazepam	Risk of falls and hip fracture, prolonged reaction time, psychiatric reactions (which can be paradoxical, e.g. agitation, irritability, hallucinations, psychosis), cognitive impairment and depression	Use the lowest possible dose, up to half of the usual dose, taper in and out, shortest possible duration of treatment. P Reduce dose, e.g. 0.5 mg/d; start low—go slow. E, M For induction of anaesthesia in older, poor-risk people, titrate dose carefully; administer in small intravenous increments of 0.3 to 0.5 mg, at 30-s intervals. M	Non-pharmacological treatment; mirtazapine^b^; passiflora, low doses of short-acting benzodiazepines such as lormetazepam (≤0.5 mg/d), brotizolam (≤0.125 mg/d); zolpidem (≤5 mg/d), zopiclon (≤3.75 mg/d), zaleplon (≤5 mg/d); trazodone. E, P
Lormetazepam (>0.5 mg/d)		Use the lowest possible dose, up to half of the usual dose, taper in and out, shortest possible duration of treatment. P	
Temazepam		Use the lowest possible dose, up to half of the usual dose, taper in and out, shortest possible duration of treatment. P Start with 7.5 mg/d and watch individual response. M	
Zopiclone (>3.75 mg/d)		Use the lowest possible dose, up to half of the usual dose, taper in and out, shortest possible duration of treatment. P	
Zolpidem (>5 mg/d)			
Clomethiazole	Risk of respiratory depression	Reduce dose. E, M Use sedative dose 500–1000 mg at bedtime. M	
Antidepressants			
Amitriptyline	Peripheral anticholinergic side effects (e.g. constipation, dry mouth, orthostatic hypotension, cardiac arrhythmia); central anticholinergic side effects (drowsiness, inner unrest, confusion, other types of delirium); cognitive deficit; increased risk of falling	Start at half the usual daily dose, increase slowly; reduce dose; start with 10 mg 3 times per day and 20 mg at bedtime. M, E, P Its use for treating neuropathic pain may be considered appropriate, with benefits overweighting the risks. E	Non-pharmacological treatment, SSRI (except PIM: fluoxetine, paroxetine, fluvoxamine)^c^, mirtazapine^b^, trazodone. E
Nortriptyline		Use 30–50 mg/d in divided doses. E, M Its use for treating neuropathic pain may be considered appropriate, with benefits overweighting the risks. E	
Fluoxetine	CNS side effects (nausea, insomnia, dizziness, confusion); hyponatremia	Reduce dose; start with 20 mg/d; maximum dose also 20 mg/d; avoid administration at bedtime. E, M	
Paroxetine	Higher risk of all-cause mortality, higher risk of seizures, falls and fractures. Anticholinergic adverse effects	For older people or for patients with renal failure, start immediate-release tablets with 10 mg/d (12.5 mg/d if controlled-release tablets), increased by 10 mg/d (12.5 mg/d if controlled-release tablets), up to 40 mg/d (50 mg/d if controlled-release tablets). E, M	
Venlafaxine	Higher risk of all-cause mortality, attempted suicide, stroke, seizures, upper gastrointestinal bleeding, falls and fracture	Start with 25–50 mg, two times per day and increase by 25 mg/dose; for extended-release formulation start with 37.5 mg once daily and increase by 37.5 mg every 4–7 days as tolerated. E Reduce the total daily dose by 25–50 % in cases of mild to moderate renal failure. M	
Psychostimulants, agents used for ADHD and nootropics			
Piracetam	No efficacy proven; unfavourable risk/benefit profile	Reduce dose for older people and for patients with renal failure. M	Non-pharmacological treatment; consider pharmacotherapy of Alzheimer-type dementia: acetylcholinesterase, memantine. E
Anti-dementia drugs			
Ginkgo biloba	No efficacy proven; increased risk of orthostatic hypotension and fall		Non-pharmacological treatment; consider pharmacotherapy of Alzheimer-type dementia: acetylcholinesterase, memantine. E
Other systemic drugs for airway diseases			
Theophylline	Higher risk of CNS stimulant effects	Start with a 25 % reduction compared to the doses for younger people. E Start with a maximum dose of 400 mg/d; monitor serum levels and reduce doses if needed; for healthy older people (>60 years), theophylline clearance is decreased by an average of 30 %. M	
Cough suppressants, excluding combinations with expectorants			
Codeine (>2 weeks)	Higher risk of adverse events (hypotension, sweating, constipation, vomiting, dizziness, sedation, respiratory depression). Avoid use for longer than 2 weeks for persons with chronic constipation without concurrent use of laxatives and for persons with renal impairment	Start treatment cautiously for older people (especially in cases of renal failure); start low—go slow; reduce dose to 75 % of the usual dose if GFR 10–50 mL/min and to 50 % if GFR <10 mL/min. M	If used for pain management consider alternative drugs proposed for “anti-inflammatory and antirheumatic products, non-steroid (NSAID)”
Antihistamines for systemic use			
Promethazine	Anticholinergic side effects (e.g. confusion, sedation)	Reduce dose; start low—go slow. M Reduce starting dose to 6.25–12.5 mg for iv injection. M	Non-sedating, non-anticholinergic antihistamines^d^ like loratadine, cetirizine, but not terfenadine (which is PIM). E If used for insomnia see alternatives proposed for “hypnotics and sedatives”
Hydroxyzine	Anticholinergic side effects (e.g. constipation, dry mouth); impaired cognitive performance, confusion, sedation; electrocardiographic changes (prolonged QT)	Reduce dose to at least 50 % less than dose used for healthy younger people. E, M	Non-sedating, non-anticholinergic antihistamines^d^ like loratadine, cetirizine, but not terfenadine (which is PIM). E Alternative therapies depending on indication. E

Note: if nothing is stated under “Dose adjustment/special considerations of use”, this means that no suggestion was made either by the experts or in Micromedex®

*E* experts, *M* Micromedex® [32], *P* PRISCUS list [22], *L* Laroche et al. (2007) [3], *B* Beers list (2012) [18], *ACE* angiotensin-converting enzyme, *CNS* central nervous system, *ECG* electrocardiographic, *GI* gastrointestinal, *PIM* potentially inappropriate medication, *PPI* proton-pump inhibitors, *RTPC RightTimePlaceCare* [23], *SIADH* syndrome of inappropriate antidiuretic hormone secretion, *ADHD* attention deficit hyperactivity disorder

Dosage abbreviations: *CrCl* creatinine clearance, *d* day, *GFR* glomerular filtration rate, *iv* intravenous, *mcg* micrograms, *mg* milligram, *min* minute, *mL* millilitre, *q* every

^a^Only the details on the drugs most commonly used in the RTPC database are presented—see also EU(7)-PIM long version in Appendix 1

^b^Caution: this drug was judged to be questionable PIM

^c^The following drugs belonging to this medication group were judged to be questionable PIM: citalopram, sertraline, and escitalopram

^d^In the group of non-sedating antihistamines, only loratadine was evaluated and judged to be questionable PIM; other drugs such as cetirizine were not evaluated

Appendix 1 shows the complete EU(7)-PIM list, which comprises 275 chemical substances (i.e. 7-digit ATC codes; e.g. amitriptyline) including two combinations of two chemical substances, plus seven drug classes (i.e. 5-digit ATC codes; e.g. triptans), belonging to 55 therapeutic classes (i.e. 4-digit ATC codes; e.g. antidepressants) and 34 therapeutic groups (i.e. 3-digit ATC codes; e.g. the nervous system). Some PIM concepts are dose-related (e.g. zopiclone used at doses higher than 3.75 mg/day) or defined by length of use (e.g. proton-pump inhibitors used longer than 8 weeks) or drug regimen (e.g. insulin, sliding scale). Appendix 1 contains also information on the number of experts who assessed each PIM, the mean, median and standard deviation of the scores given by experts to each drug (Likert scale), and the results of the compilation and selection of suggestions for dose adjustments and therapeutic alternatives. Furthermore, Appendix 1 shows two categories of those drugs (active substances characterised by their ATC code) on the EU-PIM list that are included also on other PIM lists. Category A means that precisely this active substance is named as a PIM which should be avoided in older people. Category B means that (i) this active substance is characterised as a PIM only in the case of certain clinical conditions or co-morbidities or (ii) this active substance is not specifically named but considered as a PIM drug class (e.g. anticholinergics or long-acting benzodiazepines). This information refers to six international PIM lists or criteria [3, 18, 19, 22, 26, 33] and shows that 24 drugs do not appear as PIM in any of the other lists, while the rest varies from appearing in one list only to appearing in all the lists.

The full lists of questionable PIM and non-PIM and the results of their assessments are presented in Appendix 2 and 3, respectively.

## Discussion

We developed the EU(7)-PIM list in order to analyse the prescription patterns of potentially inappropriate medication (PIM) across several European countries, and more specifically among the people with dementia participating in the *RightTimePlaceCare* Seventh Framework European project [23]. We also aimed to develop a list that would be applicable in clinical practice. The development of the EU(7)-PIM list took several international PIM lists (i.e. the German PRISCUS list [22], the American Beers list [18, 24, 25], the Canadian list [26], and the French list [3]) into consideration, as well as further drugs suggested by experts on geriatric prescribing from seven European countries who belonged to different professions.

The EU(7)-PIM list can be seen as a screening tool for the identification of PIM for older people across many European countries. We have covered several regions of Europe including Finland and Sweden in Scandinavia, France and Spain in southern Europe, Germany and the Netherlands in central Europe, and Estonia in eastern Europe. As shown by Fialová et al. [5], the prevalence of PIM use in several European countries varies widely, depending on the PIM criteria set. Thus, the creation of a PIM list suitable for pharmacoepidemiological studies and clinical use in Europe seems to be mandatory. Attempts are being undertaken to develop prescribing quality indicators which are useful for the electronic monitoring of the quality of prescribing in older people in Europe [34], and the EU(7)-PIM list could represent a part of this.

We expect the EU(7)-PIM list to be a sensitive tool because of its inclusive development process. In contrast, other tools have been seen to be less sensitive, motivating some authors to use two or three assessment tools for the assessment of PIM use in their populations in order to increase the sensitivity [5, 6, 35, 36].

We aimed at developing a list which can be used even if the clinical information available is minimal. Therefore, we chose to develop explicit PIM criteria, restricted to drugs or drug classes, in some instances restricted to high doses or prolonged treatment duration. Thus, the EU(7)-PIM list is suitable for pharmacoepidemiological applications using administrative databases or surveys without any clinical information about the individuals concerned.

To the best of our knowledge, this is the first list focusing on chemical substances and requiring only a small amount of clinical data for its application that has been developed taking into account several existing PIM lists and European markets, and that has been consented by experts from different European countries. This is also one of the few lists including suggestions for dose adjustments and therapeutic alternatives. Furthermore, the list enables a distinction between different drugs belonging to the same pharmacological subgroup and provides different suggestions for each of them. The recently published screening tool of older person’s prescriptions (STOPP)/screening tool to alert doctors to right treatment (START) criteria for potentially inappropriate prescribing for older people (version 2) were developed also with the participation of a European panel of experts [19]. However, these criteria often consider as PIM the use of pharmacological subgroups (e.g. thiazide diuretics) within specific clinical contexts (e.g. history of gout, or current significant hypokalaemia). Thus, the application of the START/STOPP criteria (both versions 1 and 2) [4, 19] requires clinical information, making these criteria more suitable in the clinical context for a comprehensive drug review of individual patients.

The development process of the EU(7)-PIM list resembles those of most other PIM lists, such as the French list [3], the German PRISCUS list [22], the Austrian PIM list [37], but also the most recent Beers list [18]. One major aspect of criticism of all PIM lists is that the classification of PIM is usually done without using evidence derived from randomised, controlled trials and relies on the expertise of the participants in the Delphi process [38]. However, this is partially justified by the lack of evidence on drug efficacy and safety in older people, due to their low enrolment in clinical trials [17]. In our study, we identified relevant literature and used it during the development process, but we did not systematically review and report it, which may be seen as a limitation.

The Delphi technique has also been criticised because of the lack of one standardised method, the difficulties in analysing the data, the difficulties in defining what an expert is, the often heterogeneous expert group, and the vague concept of consensus [38]. In order to minimise the limitations of the Delphi technique, in the present study, the characteristics of the survey were predefined (e.g. steps, consensus concept), and researchers provided experts with all necessary information to favour their engagement and participation. Researchers compiled discussion issues raised by the experts and took them into consideration for the consecutive steps of the development process.

Only seven European countries participated in the development of the EU(7)-PIM list (Estonia, Finland, France, Germany, the Netherlands, Spain and Sweden). Furthermore, the number of experts participating from some countries was limited. Certain drugs may not have been assessed for appropriateness because they were neither included in the preliminary list nor were they suggested by the experts. Certain drugs were classified as PIM with a lower level of expert agreement than others; some disagreements seemed related to the experts’ country of origin, which may show that there are international differences in prescription patterns or attitudes. Regular updates of the list should take into consideration the inclusion of other European markets, the changes in the drug markets, the prescribing tendencies, and above all, the new existing evidence.

The application of the EU(7)-PIM list cannot substitute the individual assessment of prescribing appropriateness, which should take into account other aspects such as the aims of the treatment, individual responses, and the older person’s functional level, values and preferences, among others [39]. This limitation has been recognised in the literature with regard to most tools assessing appropriateness of prescription [16]. Despite its limitations, the concept of PIM suggests that their use should be associated with less favourable outcomes. Indeed, the use of PIM has been found associated with a higher rate of adverse drug reactions in several studies, as reported in a systematic review [40], with some variations depending on the settings studied. Other authors have suggested an association between PIM use and other adverse outcomes such as injuries [41] and hospitalisation [6, 14]. A limited number of studies on interventions involving the use of some of these tools have suggested benefits in terms or relevant outcomes [42–44]. However, according to a recent systematic review, it is unclear whether such interventions result in clinically significant improvements, although benefits in terms of reducing inappropriate prescribing may exist [45].

Future research should study whether the use of PIM according to the EU(7)-PIM list shows any association with clinically relevant outcomes for older people, and whether the application of the list is associated with any benefits, both in a population and on individual levels. The acceptability of the list among health professionals should also be investigated, including the usefulness of the suggestions for drug adjustments and therapeutic alternatives.

In conclusion, the EU(7)-PIM list is an expert-consensus list of potentially inappropriate medications for older people, which was developed taking into consideration the medications appearing in six country-specific PIM lists, as well as medications used in seven European countries. It is an explicit list of chemical substances and contains suggestions for dose adjustments and therapeutic alternatives. It can be applied as a screening tool to identify potentially inappropriate medications in databases where little clinical information is available and in individual data. It can also be used for international comparisons of the prescription patterns of PIMs and may be used as a guide in the clinical practice. The application of the EU(7)-PIM list is a first step towards the identification of areas of improvement in both individual and population levels and towards the harmonisation of the prescription quality throughout Europe.

## Electronic supplementary material

ESM 1 (DOCX 586 kb)

ESM 2 (DOCX 25.9 kb)

ESM 3 (DOCX 19 kb)
