# Supplementary material for: The EU(7)-PIM list: a list of potentially inappropriate medications for older people consented by experts from seven European countries
Source: Eur J Clin Pharmacol. 2015 May 14;71(7):861–75. doi: 10.1007/s00228-015-1860-9 (PMC4464049; doi:10.1007/s00228-015-1860-9)
Supplement: Supplementary file 1 — (DOCX 586 kb) [file 228_2015_1860_MOESM1_ESM.docx]

| **ATC-Code** (according to WHO ATC-code [30] (2011)) | **Potentially inappropriate drugs**  Lists or criteria which include the specific drug (following either category A or B)^a^:  1: Laroche (2007) [3]  2: McLeod (1997) [26]  3: Finnish (2013) [33]  4: PRISCUS (2010) [22]  5: Beers (2012) [18]  6: STOPP/START (2014) [19] | **Results of the Delphi survey**  (number of experts’ answers at decisive Delphi round^b^; Likert-scale mean value [95% CI]; median) | **Main reason for PIM** | **Dose adjustment/special considerations of use** | **Alternative drugs and/or therapies** |
| --- | --- | --- | --- | --- | --- |
| **A** | **Alimentary tract and metabolism** |  |  |  |  |
| ***A02*** | ***Drugs for acid- related disorders*** |  |  |  |  |
| ***A02A*** | ***Antacids*** |  |  |  |  |
| A02AA04 | Magnesium hydroxide  In lists: 3 (A) | 20; 2.50 [2.01-2.99]; 2.00 | Risk of hypermagnesemia, which is higher in moderate to severe renal failure | Maximum dose: 5 ml/8h; reduce dose for moderate to severe renal failure. *E* | Used as laxative: osmotically active laxatives (macrogol, lactulose) *E* Used as antacid, when indication is appropriate: PPI (<8 weeks, low dose) *E* |
| A02AB, A02AD | Aluminium-containing antacids  In lists: 3 (A); 6 (B) | 23; 2.09 [1.72-2.45]; 2.00 | Renal excretion of aluminium decreases in older individuals. Risk of CNS toxicity | Adjust dose in severe renal failure. *M* Use for short periods (3-4 days). *E* | When indication is appropriate: PPI (<8 weeks, low dose) *E* |
| ***A02B*** | ***Drugs for peptic ulcer and gastro-oesophageal reflux disease*** |  |  |  |  |
| A02BA01 | Cimetidine  In lists: 1 (A); 2, 5 (B) | 23; 1.43 [1.18-1.69]; 1.00 | CNS adverse effects including confusion | 200 mg four times daily or 300 mg twice daily, due to a decrease in renal and hepatic function in adults aged ≥65 years old. *M* | When indication is appropriate: PPI (<8 weeks, low dose) *E* |
| A02BA02 | Ranitidine  In lists: 5 (B) | 23; 2.26 [1.84-2.68]; 2.00 | CNS adverse effects including confusion | CrCl <50 ml/min: 150 mg c/24h (oral); 50 mg c/18-24 h (iv) *E* | When indication is appropriate: PPI (<8 weeks, low dose) *E* |
| A02BA03 | Famotidine  In lists: 5 (B) | 23; 2.17 [1.84-2.51]; 2.00 | CNS adverse effects including confusion | CrCl <50 ml/min: administer 50% of dose or increase the dosing interval to every 36-48 h. *E* | When indication is appropriate: PPI (<8 weeks, low dose) *E* |
| A02BC | Proton pump inhibitors (PPI) (>8 weeks) e.g. omeprazole, pantoprazole  In lists: 6 (B) | 21; 2.00 [1.57-2.43]; 2.00 | Long-term high dose PPI therapy is associated with an increased risk of *C. difficile* infection and hip fracture. Inappropriate if used >8 weeks in maximal dose without clear indication |  | When indication is appropriate: PPI (<8 weeks, low dose) *E* |
| ***A03*** | ***Drugs for functional gastrointestinal disorders*** |  |  |  |  |
| ***A03A*** | ***Drugs for functional bowel disorder*** |  |  |  |  |
| A03AA04 | Mebeverine^c^  In lists: does not appear as PIM | 20; 1.60 [1.16-2.04]; 1.00 | Side effects such as dizziness, insomnia, anorexia | Caution if marked renal insufficiency. *M* Use only for short periods. *E* | Non-pharmacological measures, e.g. diet. *E* |
| A03AA05 | Trimebutine  In lists: 1, 2, 6 (B) | 19; 1.47 [1.07-1.88]; 1.00 | Anticholinergic and antimuscarinic side effects like agitation, sedation or confusion; no proven efficacy |  | Non-pharmacological measures, e.g. diet. *E* |
| A03AA08 | Dihexyverine  In lists: 1 (A); 2, 6 (B) | 14; 1.57 [1.03-2.11]; 1.00 | Anticholinergic and antimuscarinic side effects like agitation, sedation or confusion; no proven efficacy |  | Phloroglucinol. *L*  Non-pharmacological measures, e.g. diet. *E, McL* |
| A03AB06 | Otilonium bromide  In lists: 2, 6 (B) | 18; 1.50 [1.07-1.93]; 1.00 | Anticholinergic and antimuscarinic side effects like agitation, sedation or confusion; no proven efficacy |  | Non-pharmacological measures, e.g. diet. *E* |
| A03AB17 | Tiemonium (iodide)  In lists: 1 (A); 2, 6 (B) | 15; 1.60 [1.10-2.10]; 1.00 | Anticholinergic and antimuscarinic side effects like agitation, sedation or confusion; no proven efficacy |  | Phloroglucinol. *L*  Non-pharmacological measures, e.g. diet. *E, McL* |
| A03AX04 | Pinaverium^c^  In lists: does not appear as PIM | 18; 1.50 [1.07-1.93]; 1.00 | Side effects such as dizziness or esophageal ulceration |  | Non-pharmacological measures, e.g. diet. *E* |
| ***A03B*** | ***Belladonna and derivates, plain*** |  |  |  |  |
| A03BA03 | Hyoscyamine  In lists: 5 (A); 1, 2, 5, 6 (B) | 20; 1.05 [0.95-1.29]; 1.00 | Highly anticholinergic, substantial toxic effects in older adults and uncertain effectiveness / no proven efficacy |  | Butylscopolamine 20mg/6-12h for a short time, especially in palliative care. *E* Phloroglucinol *E* Non-pharmacological measures, e.g. diet. *E, McL* |
| A03BA04 | Belladonna alkaloids  In lists: 1, 5 (A); 2, 5, 6 (B) | 22; 1.14 [0.98-1.29]; 1.00 | Highly anticholinergic, substantial toxic effects in older adults and uncertain effectiveness / no proven efficacy |  | Butylscopolamine *E* Phloroglucinol *E, L* Non-pharmacological measures, e.g. diet. *E, McL* |
| ***A03C*** | ***Antispasmodics in combination with psycholeptics*** |  |  |  |  |
| A03CA02 | Clidinium-Chlordiazepoxide  In lists: 1, 3, 5 (A); 2, 6 (B) | 19; 1.21 [1.01-1.41]; 1.00 | Long half-life in older adults (often several days), producing prolonged sedation and increasing the risk of falls and fractures | Do not exceed chlordiazepoxide 10 mg, clidinium 5 mg/d; increase gradually and limit to the smallest effective dose. *M* | Phloroglucinol *E, L*  Non-pharmacological measures, e.g. diet. *E, McL* |
| ***A03D*** | ***Antispasmodics in combination with analgesics*** |  |  |  |  |
| A03DA02 | Pitofenone  In lists: 3 (A); 1, 2, 6 (B) | 18; 2.00 [1.55-2.45]; 2.00 | Anticholinergic side effects |  | Non-pharmacological measures, e.g. diet. *E* |
| ***A03F*** | ***Propulsives*** |  |  |  |  |
| A03FA01 | Metoclopramide  In lists: 3, 5 (A); 6 (B) | 23; 2.43 [1.97-2.90]; 2.00 | Antidopaminergic and anticholinergic effects;  may worsen peripheral arterial blood flow and precipitate intermittent claudication | Short-term use and dose reduction; CrCl <40 ml/min: 50% of normal dose; maximum dose: 20 mg/d; may be used in palliative care. *E* | Domperidone (<30 mg/d) if no contraindications. *E* |
| A03FA03 | Domperidone (>30 mg/d)^c^  In lists: does not appear as PIM | 18; 2.11 [1.70-2.53]; 2.00 | Increased risk of serious ventricular arrhythmia or sudden cardiac death in older adults | Treatment should be initiated at the lowest possible dose and titrated cautiously. *E* | Domperidone (<30 mg/d) if no contraindications. *E* |
| A03FA05 | Alizapride  In lists: 1 (A) | 19; 1.53 [1.23-1.82]; 1.00 | No proven efficacy; muscarinic-blocking agents; side effects such as confusion and sedation | Adjustment may be recommended in cases of renal failure. *M* |  |
| ***A04*** | ***Antiemetics and antinauseants*** |  |  |  |  |
| ***A04A*** | ***Antiemetics and antinauseants*** |  |  |  |  |
| A04AB02^g^ | Dimenhydrinate  In lists: 1, 4 (A); 5, 6 (B) | 19; 1.68 [1.29-2.08]; 1.00 | Anticholinergic side effects | Caution for patients with enlarged prostate. *E* | Domperidone (<30 mg/d) if no contraindications. *E* |
| A04AD01 | Scopolamine  In lists: 1, 3 (A); 5 (B) | 22; 1.68 [1.36-2.00]; 2.00 | Anticholinergic side effects; no proven efficacy | 5 mg/4h; may be appropriate and useful in palliative care. *E* | Domperidone (<30 mg/d) if no contraindications. *E* |
| A04AD05 | Metopimazine  In lists: 1(A) | 19; 1.68 [1.26-2.11]; 1.00 | No proven efficacy; muscarinic blocking agent; side effects such as confusion and sedation |  | Domperidone (<30 mg/d) if no contraindications. *E* |
| ***A06*** | ***Laxatives*** |  |  |  |  |
| ***A06A*** | ***Laxatives*** |  |  |  |  |
| A06AA01 | Viscous paraffin (=Liquid paraffin)  In lists: 4, 5 (A) | 21; 2.43 [1.88-2.98]; 2.00 | Pulmonary side effects if aspirated |  | Recommend proper dietary fibre and fluid intake; osmotically active laxatives: macrogol, lactulose. *E, P* |
| A06AA02 | Docusate sodium (oral)  In lists: 1 (A) | 19; 1.95 [1.57-2.32]; 2.00 | Stool softener laxative. Adverse events include cramping, nausea, diarrhoea. May exacerbate bowel disfunction |  | Recommend proper dietary fibre and fluid intake; osmotically active laxatives: macrogol, lactulose. *E, P* |
| A06AB02 | Bisacodyl (>3 days)  In lists: 1, 3 (A); 5 (B) | 21; 1.90 [1.59-2.22]; 2.00 | Stimulant laxative. Adverse events include abdominal pain, fluid and electrolyte imbalance and hypoalbuminemia. May exacerbate bowel disfunction |  | Recommend proper dietary fibre and fluid intake; osmotically active laxatives: macrogol, lactulose. *E, P* |
| A06AB05 | Castor oil (=Ricinus communis, =Neoloid)  In lists: 1 (A), 5 (B) | 21; 2.24 [1.70-2.77]; 2.00 | Stimulant laxative. Adverse events include abdominal pain, fluid and electrolyte imbalance and hypoalbuminemia. May exacerbate bowel disfunction |  | Recommend proper dietary fibre and fluid intake; osmotically active laxatives: macrogol, lactulose. *E, P* |
| A06AB06 | Senna glycosides  In lists: 3 (A) | 23; 2.35 [1.79-2.91]; 2.00 | Stimulant laxative. Adverse events include abdominal pain, fluid and electrolyte imbalance and hypoalbuminemia. May exacerbate bowel disfunction |  | Recommend proper dietary fibre and fluid intake; osmotically active laxatives: macrogol, lactulose. *E, P* |
| A06AB07 | Cascara sagrada  In lists: 1 (A); 5 (B) | 19; 2.32 [1.71-2.92]; 2.00 | Stimulant laxative. Adverse events include abdominal pain, fluid and electrolyte imbalance and hypoalbuminemia. May exacerbate bowel disfunction |  | Recommend proper dietary fibre and fluid intake; osmotically active laxatives: macrogol, lactulose. *E, P* |
| A06AB08 | Sodium picosulfate  In lists: 1, 3 (A) | 22; 2.32 [1.82-2.82]; 2.00 | Stimulant laxative. Adverse events include abdominal pain, fluid and electrolyte imbalance and hypoalbuminemia. May exacerbate bowel dysfunction |  | Recommend proper dietary fibre and fluid intake; osmotically active laxatives: macrogol, lactulose. *E, P* |
| A06AB13^g^ | Aloe  In lists: 1 (A) | 16; 2.13 [1.65-2.60]; 2.00 | Stimulant laxative. Adverse events include abdominal pain, fluid and electrolyte imbalance and hypoalbuminemia. May exacerbate bowel disfunction |  | Recommend proper dietary fibre and fluid intake; osmotically active laxatives: macrogol, lactulose. *E, P* |
| A06AX05^h^ | Prucalopride  In lists: does not appear as PIM | 11; 2.09 [1.46-2.73]; 2.00 | Adverse effects can include abdominal pain, diarrhoea, headache, dizziness | Reduce dose for older adults and in cases of severe renal failure (GFR<30 ml/min); starting dose for persons over 65 years old: 1 mg/d; maximum dose: 2 mg/d (1 mg/d if severe renal failure) *E, M* | Recommend proper dietary fibre and fluid intake; osmotically active laxatives: macrogol, lactulose. *E, P* |
| ***A07*** | ***Antidiarrhoeal, intestinal anti-inflammatory / anti-infective agents*** |  |  |  |  |
| ***A07D*** | ***Antipropulsives*** |  |  |  |  |
| A07DA01 (Diphenoxylate) A03BA01 (Atropine) | Diphenoxylate-Atropine  In lists: 1 (A); 2, 5, 6 (B) | 22; 1.73 [1.29-2.16]; 1.00 | No proven efficacy; muscarinic blocking agent |  | Non-pharmacological measures, e.g. diet. *E* Phloroglucinol *L* |
| A07DA03 | Loperamide (>2 days)  In lists: does not appear as PIM | 21; 1.81 [1.47-2.15]; 2.00 | Risk of somnolence, constipation, nausea, abdominal pain and bloating. Rare adverse events include dizziness. May precipitate toxic megacolon in inflammatory bowel disease, may delay recovery in unrecognized gastroenteritis | Start with a dose of 4 mg followed by 2 mg in each deposition until normalisation of bowel; do not exceed 16 mg/d; use no longer than 2 days; may be useful in palliative care for persisting non-infectious diarrhoea. *E* | Non-pharmacological measures, e.g. diet; phloroglucinol. *E* |
| ***A07X*** | ***Other antidiarrheals*** |  |  |  |  |
| A07XA04 | Racecadotril  In lists: does not appear as PIM | 16; 2.31 [1.68-2.95]; 2.00 | No proven efficacy; selective inhibitor of enkephalinase enzyme responsible for the degradation of the enkephalins, endogenous opioids which act by decreasing the intestinal lumen secretion of water and electrolytes | Maximum dose 100 mg/8h; maximum duration 7 days. *E* | Non-pharmacological measures, e.g. diet. *E* |
| ***A10*** | ***Drug used in Diabetes*** |  |  |  |  |
| ***A10A*** | ***Insulins and analogues*** |  |  |  |  |
| no ATC, treatment concept PIM | Insulin, sliding scale  In lists: 5 (A) | 13; 2.00 [1.45-2.55]; 2.00 | No benefits demonstrated in using sliding-scale insulin. Might facilitate fluctuations in glycemic levels | Lower doses to avoid hypoglycemia. *E* | Basal insulin. *E* |
| ***A10B*** | ***Blood glucose lowering drugs, excl. insulins*** |  |  |  |  |
| A10BB01 | Glibenclamide  In lists: 1, 5 (A); 6 (B) | 23; 2.00 [1.55-2.45]; 2.00 | Risk of protracted hypoglycemia | Use conservative initial dose (1.25 mg/d for nonmicronized glibenclamide and 0.75 mg/d for micronized glibenclamide) and maintenance dose; not recommended if CrCl <50 ml/min. *M* | Diet; metformin (<2 x 850 mg/d); insulin; gliclazide may be safer than the other short-acting sulphonilureas. *E* |
| A10BB02 | Chlorpropamide  In lists: 5 (A); 1, 6 (B) | 20; 1.40 [1.12-1.68]; 1.00 | Risk of protracted hypoglycemia | Use initial doses of 100 to 125 mg/d. *M* In cases of mild renal failure (GFR >50 ml/min), decrease dose by 50%. *M, E* In cases of moderate to severe renal failure (GFR <50 ml/min), avoid. *M* | Diet; metformin (<2 x 850 mg/d); insulin; gliclazide may be safer than the other short-acting sulphonilureas. *E* |
| A10BB06 | Carbutamide  In lists: 1 (A), 6 (B) | 16; 2.06 [1.61-2.52]; 2.00 | Risk of protracted hypoglycemia | Adjust dose to renal function. *E* | Diet; metformin (<2 x 850 mg/d); insulin; gliclazide may be safer than the other short-acting sulphonilureas. *E* |
| A10BB07 | Glipizide  In lists: 1 (A) | 22; 2.45 [2.01-2.90]; 2.00 | Risk of protracted hypoglycemia | Use conservative initial and maintenance doses. *M* Starting dose: 2.5 mg/d *E, M* Increase by 2.5-5 mg/d at 1 to 2 week intervals. *E* | Diet; metformin (<2 x 850 mg/d); insulin; gliclazide may be safer than the other short-acting sulphonilureas. *E* |
| A10BB12 | Glimepiride  In lists: 3 (A); 6 (B) | 21; 2.05 [1.71-2.38]; 2.00 | Risk of protracted hypoglycemia | Adjust according to renal function. *E* For patients with renal failure and for older adults, use initial dose of 1 mg/d followed by a conservative titration scheme. Titrate dose in increments of 1 to 2 mg no more than every 1 to 2 weeks based on individual glycemic response. *M* | Diet; metformin (<2 x 850 mg/d); insulin; gliclazide may be safer than the other short-acting sulphonilureas. *E* |
| A10BF01 | Acarbose  In lists: does not appear as PIM | 23; 2.22 [1.68-2.75]; 2.00 | No proven efficacy |  | Diet; metformin (<2 x 850 mg/d); insulin; gliclazide may be safer than the other short-acting sulphonilureas. *E* |
| A10BG03 | Pioglitazone  In lists: 5, 6 (B) | 21; 1.71 [1.42-2.01]; 2.00 | Age-related risks include bladder cancer, fractures and heart failure. Use for more than one year has been associated with an increased risk of bladder cancer. May increase the incidence of fractures of the upper arms, hands and feet in female diabetics (compared to other oral antidiabetic agents). Can cause fluid retention in older adults, which may exacerbate or precipitate heart failure |  | Diet; metformin (<2 x 850 mg/d); insulin; gliclazide may be safer than the other short-acting sulphonilureas. *E* |
| A10BH01 | Sitagliptine  In lists: does not appear as PIM | 17; 1.94 [1.44-2.44]; 2.00 | Limited safety data is available for adults aged ≥75 years old. Subjects aged 65 to 80 years had higher plasma concentrations than younger subjects. Risk of hypoglycemia, dizziness, headache and peripheral oedema | Reduce dose to 50 mg/d in cases of renal failure (CrCl 30-50 ml/min); reduce dose to 25 mg/d in cases of severe renal insufficiency (CrCl <30 ml/min). *E, M* | Diet; metformin (<2 x 850 mg/d); insulin; gliclazide may be safer than the other short-acting sulphonilureas. *E* |
| A10BH02 | Vildagliptine  In lists: does not appear as PIM | 15; 1.87 [1.21-2.52]; 2.00 | Limited safety data available in older subjects. In healthy older adults (≥70 years) the overall exposure of vildagliptin (100 mg once daily) was increased by 32%, with an 18% increase in peak plasma concentration as compared to young healthy subjects (18-40 years). Adverse events (general population) include risk of hypoglycemia, dizziness, headache and peripheral oedema | Reduce dose to 50 mg/d in cases of moderate or severe renal failure. *E, M* | Diet; metformin (<2 x 850 mg/d); insulin; gliclazide may be safer than the other short-acting sulphonilureas. *E* |
| **B** | **Blood and blood forming organs** |  |  |  |  |
| ***B01*** | ***Antithrombotic agents*** |  |  |  |  |
| ***B01A*** | ***Antithrombotic agents*** |  |  |  |  |
| B01AA07 | Acenocoumarol  In lists: 6 (B) | 17; 2.35 [1.84-2.87]; 2.00 | Risk of bleeding, especially in people with difficult control of INR value |  |  |
| B01AC05 | Ticlopidine  In lists: 1, 4, 5, 6 (A); 6 (B) | 20; 1.70 [1.36-2.04]; 2.00 | Risk of altered blood counts | Dose reductions may be required in cases of renal failure. *M* | Clopidogrel; aspirin (<325mg)^d^. *E, L* |
| B01AC07 | Dipyridamole  In lists: 1, 2, 3, 5 (A); 6 (B) | 22; 2.14 [1.70-2.58]; 2.00 | Less efficient than aspirin; risk of vasodilatation and orthostatic hypotension Proven beneficial only for patients with artificial heart valves |  | Clopidogrel; aspirin (<325mg)^d^. *E, L* |
| B01AC22 | Prasugrel  In lists: 4 (A); 6 (B) | 18; 2.00 [1.41-2.59]; 2.00 | Unfavourable risk/benefit profile, especially for adults aged 75 and older |  | Clopidogrel; aspirin (<325mg)^d^. *E, L* |
| B01AE07 | Dabigatran^c^  In lists: 6 (B) | 22; 2.45 [2.01-2.90]; 2.00 | Limited information on use for older adults and on the risk of bleeding events in this population; no reversal agent is available in case of overdose | Reduce dose for adults aged >75 years old (150 mg/d) and CrCl 30-50 (110 mg twice per day); contraindicated if CrCl <30. *E* |  |
| B01AF01^g, h^ | Rivaroxaban^c^  In lists: 6 (B) | 19; 2.42 [2.02-2.82]; 2.00 | Limited information on use for older adults; risk of bleeding events; no reversal agent available in case of overdose; risk of bleeding may be higher in cases of severe renal failure | Reduce dose for adults aged >65 years and avoid use for persons with CrCl <30 ml/min. *E, M* |  |
| B01AF02^i^ | Apixaban^c^  In lists: 6 (B) | 16; 2.25 [1.75-2.75]; 2.00 | Limited information on use for older adults; risk of bleeding events; no reversal agent available in case of overdose | Reduce dose to 2.5 mg orally twice daily for patients with any 2 of the following *(M)* (1 of the following *(E))*: ≥80 years old, body weight ≤60 kg, or serum creatinine ≥1.5 mg/dL. Do not use if CrCl less than 15 mL/min or if undergoing dialysis; reduce dose to 2.5 mg twice per day in cases of severe renal failure (CrCl 15 mL/min to 29 mL/min); no dosage adjustment necessary in cases of mild (CrCl 51 to 80 mL/min) or moderate (CrCl 30 to 50 mL/min) renal failure. *M* |  |
| ***B03*** | ***Antianemic preparations*** |  |  |  |  |
| ***B03A*** | ***Iron preparations*** |  |  |  |  |
| B03AA | Iron supplements / Ferrous sulfate (>325 mg/d)  In lists: 6 (B) | 23; 2.22 [1.68-2.75]; 2.00 | Doses >325 mg/d do not considerably increase the amount absorbed but greatly increase the incidence of constipation |  | Intravenous iron *E* |
| **C** | **Cardiovascular system** |  |  |  |  |
| ***C01*** | ***Cardiac therapy*** |  |  |  |  |
| ***C01A*** | ***Cardiac glycosides*** |  |  |  |  |
| C01AA02 | Acetyldigoxin  In lists: 4 (A) | 14; 2.14 [1.47-2.82]; 2.00 | Elevated glycoside sensitivity in older adults (women >men); risk of intoxication | Calculate digitalizing doses based on lean body mass and maintenance doses using actual CrCl. *M* | For tachycardia/atrial fibrillation: beta-blockers (except oxprenolol, pindolol, propranolol, sotalol, nadolol, labetalol). *E, P* For congestive heart failure: diuretics (except spironolactone >25 mg/d), ACE-inhibitors. *E* |
| C01AA04 | Digitoxin  In lists: does not appear as PIM | 16; 2.19 [1.57-2.87]; 2.00 | Elevated glycoside sensitivity in older adults (women >men); risk of intoxication | Calculate digitalizing doses based on lean body mass and maintenance doses using actual CrCl. *M* | For tachycardia/atrial fibrillation: beta-blockers (except oxprenolol, pindolol, propranolol, sotalol, nadolol, labetalol). *E, P* For congestive heart failure: diuretics (except spironolactone >25 mg/d), ACE-inhibitors. *E* |
| C01AA05 | Digoxin  In lists: 4, 5 (A); 1, 6 (B) | 23; 2.35 [1.92-2.77]; 2.00 | Elevated glycoside sensitivity in older adults (women >men); risk of intoxication | Calculate digitalizing doses based on lean body mass and maintenance doses using actual CrCl. *M* For older adults, use dose 0.0625-0.125mcg/d; in cases of renal failure (CrCl 10-50 ml/min), administer 25-75% of dose or every 36 hours; in cases of renal failure (CrCl <10 ml/min), administer 10-25% of dose or every 48 hours. *E* | For tachycardia/atrial fibrillation: beta-blockers (except oxprenolol, pindolol, propranolol, sotalol, nadolol, labetalol). *E, P* For congestive heart failure: diuretics (except spironolactone >25 mg/d), ACE-inhibitors. *E* |
| C01AA08 | Metildigoxin  In lists: 4 (A) | 15; 2.20 [1.57-2.83]; 2.00 | Elevated glycoside sensitivity (women >men); risk of intoxication | Calculate digitalizing doses based on lean body mass and maintenance doses using actual CrCl. *M* In old adults with heart failure and normal renal function, oral maintenance dose requirement of digoxin is 1.4 times higher than metildigoxin. *M* | For tachycardia/atrial fibrillation: beta-blockers (except oxprenolol, pindolol, propranolol, sotalol, nadolol, labetalol). *E, P* For congestive heart failure: diuretics (except spironolactone >25 mg/d), ACE-inhibitors. *E* |
| ***C01B*** | ***Antiarrhythmics, Class I and III*** |  |  |  |  |
| C01BA01 | Quinidine  In lists: 3, 4, 5 (A) | 23; 1.48 [1.22-1.73]; 1.00 | CNS side effects; increased mortality.  Data suggest that for most older adults rate control yields better balance of benefits and harms than rhythm control *B* |  | Beta-blockers (except oxprenolol, pindolol, propranolol, sotalol, nadolol, labetalol). *E, P* |
| C01BA02 | Procainamide  In lists: 5 (A) | 21; 1.76 [1.41-2.11]; 2.00 | High risk of drug interactions. Data suggest that for most older adults rate control yields better balance of benefits and harms than rhythm control *B* | Adjust dose to the individual patient response. Lower doses or longer intervals between doses may be required. *M*  CrCl 10-50 ml/min administer every 6-12 h; CrCl <10 ml/min administer every 8-24 h. *E* | Beta-blockers (except oxprenolol, pindolol, propranolol, sotalol, nadolol, labetalol). *E, P* |
| C01BA03 | Disopyramide  In lists: 1, 2, 5 (A) | 23; 1.43 [1.18-1.69]; 1.00 | Potent negative inotrope; anticholinergic side effects; may induce heart failure; may cause sudden cardiac death. Data suggest that for most older adults rate control yields better balance of benefits and harms than rhythm control *B* | Start dose at the lower end of the dosing range and titrate upward to maximum dose as required for antiarrhythmic effects and based on CrCl. *M* | Beta-blockers (except oxprenolol, pindolol, propranolol, sotalol, nadolol, labetalol). *E, P* |
| C01BA51 | Quinidine in combination with verapamil  In lists: 4 (A) | 22; 1.36 [1.15-1.58]; 1.00 | CNS side effects and increased mortality. Data suggest that for most older adults rate control yields better balance of benefits and harms than rhythm control *B* |  | Beta-blockers (except oxprenolol, pindolol, propranolol, sotalol, nadolol, labetalol). *E, P* |
| C01BC03 | Propafenone  In lists: 3, 5 (A) | 19; 1.89 [1.44-2.35]; 1.00 | High risk of drug interactions. Data suggest that for most older adults rate control yields better balance of benefits and harms than rhythm control *B* | Start dose at the lower end of the dosing range and increase gradually. *M* A single oral 600 mg loading dose may be effective for converting recent-onset atrial fibrillation to sinus rhythm in persons older than 60 years without signs or symptoms of heart failure. *M* | Beta-blockers (except oxprenolol, pindolol, propranolol, sotalol, nadolol, labetalol). *E, P* |
| C01BC04 | Flecainide  In lists: 3, 4, 5 (A) | 22; 2.14 [1.66-2.62]; 2.00 | Higher rate of adverse effects, especially in older adults. Data suggest that for most older adults rate control yields better balance of benefits and harms than rhythm control *B* | Adjust dose in cases of renal failure. *M* | Beta-blockers (except oxprenolol, pindolol, propranolol, sotalol, nadolol, labetalol). *E, P* |
| C01BD01 | Amiodarone  In lists: 3, 5 (A); 6 (B) | 23; 2.30 [1.81-2.80]; 2.00 | Associated with QT interval problems and risk of provoking torsades de pointes. Data suggest that for most older adults rate control yields better balance of benefits and harms than rhythm control *B* | Start dose at the low end of the dosing range. *M* Use lower maintenance dose, e.g. 200 mg/48h. *E* |  |
| C01BD07 | Dronedarone  In lists: 3, 5 (A) | 21; 1.57 [1.23-1.91]; 2.00 | Frequent drug interactions; prolonged QT interval; not recommended in permanent atrial fibrillation; increased mortality due to cardiovascular causes. Data suggest that for most older adults rate control yields better balance of benefits and harms than rhythm control *B* |  |  |
| ***C01E*** | ***Other cardiac preparations*** |  |  |  |  |
| C01EB15 | Trimetazidine  In lists: does not appear as PIM | 13; 1.62 [1.22-2.01]; 2.00 | Can cause or worsen parkinsonian symptoms (tremor, akinesia, hypertonia); caution in cases of moderate renal failure and with older adults (>75 years old); efficacy for the treatment of tinnitus or dizziness not proven | 20 mg twice per day for patients with moderate renal insufficiency. *E* |  |
| C01EB17 | Ivabradine  In lists: does not appear as PIM | 16; 2.13 [1.61-2.64]; 2.00 | Common adverse events (1-10% of patients) may include first-degree AV block, ventricular extrasystoles, dizziness and blurred vision | Lower initial dose for older adults; starting dose 2 x 2.5 mg/d in >75 years. *M, E* Use with caution for patients with CrCl less than 15 mL/min*. M* |  |
| ***C02*** | ***Antihypertensives*** |  |  |  |  |
| ***C02A*** | ***Antiadrenergic agents, centrally acting*** |  |  |  |  |
| C02AA02 | Reserpine  In lists: 1, 2, 4, 5 (A); 6 (B) | 20; 1.25 [1.04-1.46]; 1.00 | Risk of orthostatic hypotension, bradycardia, syncope, CNS side effects (sedation, depression, cognitive impairment) | Low initial dose, half of usual dose, taper in and out. *P* Lower doses (0.05 mg/d) to normal doses (0.25 mg/d) are recommended. *M* Avoid if CrCl <10 ml/min. *M, E* | Other antihypertensive drugs, e.g. ACE inhibitors, or other mediation groups depending on comorbidity (exclude PIM). *E* |
| C02AB01 | Methyldopa  In lists: 1, 4, 5 (A); 6 (B) | 21; 1.38 [1.11-1.65]; 1.00 | Risk of orthostatic hypotension, bradycardia, syncope, CNS side effects (sedation, depression, cognitive impairment) | Low initial dose, half of usual dose, taper in and out. *P* Suggested initial daily dose is 250 mg of methyldopa with a maximal daily dose of 1000 mg. *M* CrCl >50 ml/min administer every 8 h; CrCl 10-50 ml/min administer every 8-12 h; CrCl <10 ml/min administer every 12-24 h. *E* | Other antihypertensive drugs, e.g. ACE inhibitors, or other mediation groups depending on comorbidity (exclude PIM). *E* |
| C02AC01 | Clonidine  In lists: 1, 3, 4, 5 (A); 6 (B) | 22; 1.36 [1.04-1.69]; 1.00 | Risk of orthostatic hypotension, bradycardia, syncope, CNS side effects (sedation, depression, cognitive impairment) | Lower doses for initial treatment of hypertension; half of usual dose, taper in and out. *M, P* | Other antihypertensive drugs, e.g. ACE inhibitors, or other medication groups depending on comorbidity (exclude PIM). *E* |
| C02AC02 | Guanfacine  In lists: 1, 5 (A); 6 (B) | 19; 1.42 [1.13-1.71]; 1.00 | Risk of orthostatic hypotension, bradycardia, syncope, CNS side effects (sedation, depression, cognitive impairment) | Cautious dosing when using guanfacine hydrochloride immediate-release; start dosing at the low end of the range. *M* | Other antihypertensive drugs, e.g. ACE inhibitors, or other medication groups depending on comorbidity (exclude PIM). *E* |
| C02AC05 | Moxonidine  In lists: 1, 3 (A); 6 (B) | 22; 1.77 [1.34-2.20]; 1.50 | Risk of orthostatic hypotension, bradycardia, syncope, CNS side effects (sedation, depression, cognitive impairment) | Caution in cases of moderate renal insufficiency (CrCl 30-60 ml/min): maximum doses 0.4 mg/d; avoid if CrCl <30ml/min. *M, E* | Other antihypertensive drugs, e.g. ACE inhibitors, or other medication groups depending on comorbidity (exclude PIM). *E* |
| C02AC06 | Rilmenidine  In lists: 1 (A); 6 (B) | 17; 1.53 [1.16-1.90]; 1.00 | Risk of orthostatic hypotension, bradycardia, syncope, CNS side effects (sedation, depression, cognitive impairment) | Reduce dose in cases of renal failure (CrCl <15 ml/min), *M, E* | Other antihypertensive drugs, e.g. ACE inhibitors, or other medication groups depending on comorbidity (exclude PIM). *E* |
| ***C02C*** | ***Antiadrenergic agents, peripherally acting*** |  |  |  |  |
| C02CA01 | Prazosin  In lists: 1, 3, 4, 5 (A); 6 (B) | 20; 1.55 [1.27-1.83]; 1.50 | Higher risk of orthostatic hypotension, dry mouth, urinary incontinence/ impaired micturition, CNS side effects (e.g. vertigo, light-headedness, somnolence) and cerebrovascular and cardiovascular disease | Lower dose for initial treatment of hypertension. *M* Start with half of usual dose, taper in and out. *P* First dose given at bedtime: initial 1-2 mg/d. *E* | Other antihypertensive drugs, e.g. ACE inhibitors, or other medication groups depending on comorbidity (exclude PIM). *E* |
| C02CA04 | Doxazosin  In lists: 4, 5 (A); 6 (B) | 22; 1.95 [1.61-2.30]; 2.00 | Higher risk of orthostatic hypotension, dry mouth, urinary incontinence/ impaired micturition, CNS side effects (e.g. vertigo, light-headedness, somnolence) and cerebrovascular and cardiovascular disease | Start with half of usual dose, taper in and out. *P* Start with 0.5mg/d (immediate release) or 4-8 mg/d (extended release). *E* | Other antihypertensive drugs, e.g. ACE inhibitors, or other medication groups depending on comorbidity (exclude PIM). *E* |
| C02CA06 | Urapidil  In lists: 1 (A); 6 (B) | 19; 1.68 [1.29-2.08]; 1.00 | Higher risk of orthostatic hypotension, dry mouth, urinary incontinence/ impaired micturition, CNS side effects (e.g. vertigo, light-headedness, somnolence) and cerebrovascular and cardiovascular disease | Reduce dose for older adults and patients with renal insufficiency. *M* | Other antihypertensive drugs, e.g. ACE inhibitors, or other medication groups depending on comorbidity (exclude PIM). *E* |
| C02CC02 | Guanethidine  In lists: does not appear as PIM | 19; 1.58 [1.25-1.91]; 1.00 | Higher risk of orthostatic hypotension, dry mouth, urinary incontinence/ impaired micturition, CNS side effects (e.g. vertigo, light-headedness, somnolence) and cerebrovascular and cardiovascular disease | Start low–go slow; Increase dose interval in cases of renal failure. *M* | Other antihypertensive drugs, e.g. ACE inhibitors, or other medication groups depending on comorbidity (exclude PIMs). *E* |
| ***C02D*** | ***Agents acting on Arteriolar Smooth muscle*** |  |  |  |  |
| C02DB02 | Hydralazine  In lists: 6 (B) | 21; 2.33 [1.73-2.93]; 2.00 | Risk of orthostatic hypotension | Start low–go slow; Increase dose interval in cases of renal failure. *M, E* |  |
| ***C03*** | ***Diuretics*** |  |  |  |  |
| ***C03D*** | ***Potassium-sparing agent*** |  |  |  |  |
| C03DA01 | Spironolactone (>25 mg/d)^c^  In lists: 5 (A); 6 (B) | 20; 2.50 [1.99-3.01]; 2.00 | Higher risk of hyperkalaemia and hyponatremia in older adults, especially if doses >25 mg/d, requiring periodic controls | Reduce dose in cases of moderate renal insufficiency. *E, M* GFR ≥50 mL/min/1.73 m: initial dose 12.5-25 mg/d, increase up to 25 mg 1-2x/d;  GFR 30-49 mL/min/1.73 m: initial dose 12.5 mg/d, increase up to 12.5-25 mg/d; reduce dose if potassium levels increase or renal function worsens.  GFR <10 mL/min: avoid. *M* | Consider alternatives depending on the indication; exclude PIMs. |
| ***C04*** | ***Peripheral vasodilators*** |  |  |  |  |
| ***C04A*** | ***Peripheral vasodilators*** |  |  |  |  |
| C04AD03 | Pentoxifylline  In lists: 1, 2, 3, 4 (A); 6 (B) | 21; 1.95[1.42-2.48]; 2.00 | No proven efficacy; unfavourable risk/benefit profile; orthostatic hypotension and fall risks are increased with most vasodilators | Reduce dose to 400 mg twice daily in cases of moderate renal failure and to 400 mg once daily in cases of severe renal failure; close monitoring for toxicities. Avoid use if CrCl <30 ml/min. *M* |  |
| C04AE02 | Nicergoline  In lists: 1, 4 (A); 6 (B) | 19; 1.63 [1.12-2.15]; 1.00 | No proven efficacy; unfavourable risk/benefit profile; orthostatic hypotension and fall risks are increased with most vasodilators | Reduce daily dose in cases of renal failure (serum creatinine >2 mg/dl). *M* |  |
| C04AE04 | Dihydroergocristine  In lists: 1 (A), 6 (B) | 19; 1.42 [1.05-1.79]; 1.00 | No proven efficacy; unfavourable risk/benefit profile; orthostatic hypotension and fall risks are increased with most vasodilators |  |  |
| C04AE54 | Raubasine-Dihydroergocristine  In lists: 1 (A); 6 (B) | 18; 1.33 [0.99-1.67]; 1.00 | No proven efficacy; unfavourable risk/benefit profile; orthostatic hypotension and fall risks are increased with most vasodilators |  |  |
| C04AX01 | Cyclandelate (=Cyclospasmol)  In lists: 6 (B) | 18; 1.33 [1.04-1.63]; 1.00 | No proven efficacy; unfavourable risk/benefit profile; orthostatic hypotension and fall risks are increased with most vasodilators |  |  |
| C04AX07 | Vincamine  In lists: 1 (A); 6 (B) | 17; 1.53 [1.12-1.94]; 1.00 | No proven efficacy; unfavourable risk/benefit profile; orthostatic hypotension and fall risks are increased with most vasodilators |  |  |
| C04AX10 | Moxisylyte  In lists: 1 (A); 6 (B) | 17; 1.53 [1.12-1.94]; 1.00 | No proven efficacy; unfavourable risk/benefit profile; orthostatic hypotension and fall risks are increased with most vasodilators |  |  |
| C04AX17 | Vinburnine  In lists: 1 (A); 6 (B) | 17; 1.53 [1.12-1.94]; 1.00 | No proven efficacy; unfavourable risk/benefit profile; orthostatic hypotension and fall risks are increased with most vasodilators |  |  |
| C04AX20 | Buflomedil  In lists: 6 (B) | 16; 1.69 [1.08-2.29]; 1.00 | No proven efficacy; unfavourable risk/benefit profile; orthostatic hypotension and fall risks are increased with most vasodilators |  |  |
| C04AX21 | Naftidrofuryl  In lists: 1, 4 (A); 6 (B) | 17; 1.59 [1.11-2.07]; 1.00 | No proven efficacy; unfavourable risk/benefit profile; orthostatic hypotension and fall risks are increased with most vasodilators |  |  |
| ***C05*** | ***Vasoprotectives*** |  |  |  |  |
| ***C05C*** | ***Capillary stabilizing agents*** |  |  |  |  |
| C05CA05 | Hidrosmin  In lists: 6 (B) | 17; 1.82 [1.41-2.24]; 2.00 | No proven efficacy; unfavourable risk/benefit profile; orthostatic hypotension and fall risks are increased with most vasodilators |  | Compression stocking . *E* |
| C05CA07^g^ | Escin (=Aescin)  In lists: 6 (B) | 18; 1.83 [1.37-2.29]; 2.00 | No proven efficacy; unfavourable risk/benefit profile; orthostatic hypotension and fall risks are increased with most vasodilators |  | Compression stocking . *E* |
| C05CA51 | Vincamine-Rutoside  In lists: 1 (A); 6 (B) | 16; 1.75 [1.34-2.16]; 2.00 | No proven efficacy; unfavourable risk/benefit profile; orthostatic hypotension and fall risks are increased with most vasodilators |  | Compression stocking . *E* |
| C05CA54 | Troxerutin-Vincamine  In lists: 1 (A); 6 (B) | 16; 1.81 [1.33-2.30]; 2.00 | No proven efficacy; unfavourable risk/benefit profile; orthostatic hypotension and fall risks are increased with most vasodilators |  | Compression stocking . *E* |
| ***C07*** | ***Beta-blocking agents*** |  |  |  |  |
| ***C07A*** | ***Beta-blocking agents*** |  |  |  |  |
| C07AA02 | Oxprenolol  In lists: 2, 6 (B) | 16; 2.25 [1.79-2.71]; 2.00 | Non-selective beta-adrenergic blocker; may exacerbate or cause respiratory depression; possible CNS adverse events |  | Cardio-selective beta-blockers (e.g. metoprolol, bisoprolol, carvedilol, atenolol). *E* |
| C07AA03 | Pindolol  In lists: 3 (A); 2, 6 (B) | 20; 2.40 [1.91-2.89]; 2.00 | Non-selective beta-adrenergic blocker; may exacerbate or cause respiratory depression; possible CNS adverse events |  | Cardio-selective beta-blockers (e.g. metoprolol, bisoprolol, carvedilol, atenolol). *E* |
| C07AA05 | Propranolol  In lists: 3 (A); 6 (B) | 21; 2.33 [1.94-2.72]; 2.00 | Non-selective beta-adrenergic blocker; may exacerbate or cause respiratory depression; possible CNS adverse events | 3 doses of 20 mg daily *E* Start low–go slow for older adults and patients with renal failure. *M* | Depending on the indication: cardio-selective beta-blockers, ACE inhibitors, diuretics. *E* |
| C07AA07 | Sotalol  In lists: 4, 5 (A); 2, 6 (B) | 21; 1.86 [1.64-2.07]; 2.00 | Non-selective beta-adrenergic blocker; may exacerbate or cause respiratory depression; possible CNS adverse events | Start at half or one third of the typical dose and increase slowly. *P* Reduce dose and dosing interval in cases of renal failure. *M* | Cardio-selective beta-blockers (e.g. metoprolol, bisoprolol, carvedilol, atenolol). *E* |
| C07AA12 | Nadolol  In lists: 2, 6 (B) | 16; 2.44 [1.89-2.99]; 2.00 | Non-selective beta-adrenergic blocker; may exacerbate or cause respiratory depression | If CrCl 31-50 ml/min: administer every 24-36 h; if CrCl 10-30 ml/min: administer every 24-48h; if CrCl <10 ml/min: administer every 40-60 h. *E, M* | Cardio-selective beta-blockers (e.g. metoprolol, bisoprolol, carvedilol, atenolol). *E* |
| C07AG01 | Labetalol  In lists: 2, 6 (B) | 20; 2.30 [1.87-2.73]; 2.00 | Non-selective beta-adrenergic blocker; may exacerbate or cause respiratory depression | Start dose 100 mg once or twice per day. E Maintenance dose 100-200 mg once or twice per day. *M* | Cardio-selective beta-blockers (e.g. metoprolol, bisoprolol, carvedilol, atenolol). *E* |
| ***C08*** | ***Calcium channel blockers*** |  |  |  |  |
| ***C08C*** | ***Selective calcium channel blockers with mainly vascular effects*** |  |  |  |  |
| C08CA04 | Nicardipine  In lists: 1 (A); 2, 6 (B) | 19; 2.00 [1.38-2.62]; 1.00 | Risk of orthostatic hypotension, myocardial infarction or stroke | Lower initial dose. *M* | Other antihypertensive drugs (amlodipine, cardioselective beta-blockers, ACE inhibitors, diuretics). *E, L* |
| C08CA05 | Nifedipine (non-sustained-release)  In lists: 1, 4, 5 (A); 2, 6 (B) | 23; 1.74 [1.28-2.19]; 1.00 | Increased risk of hypotension; myocardial infarction; increased mortality | Lower initial dose, half of usual dose, taper in and out. *P* | Other antihypertensive drugs (amlodipine, cardioselective beta-blockers, ACE inhibitors, diuretics). *E, L* |
| C08CA05 | Nifedipine (sustained-release)  In lists: 1 (A); 2, 6 (B) | 21; 1.95 [1.51-2.40]; 2.00 | Increased risk of hypotension; myocardial infarction; increased mortality | Lower initial dose, half of usual dose, taper in and out. *P* Initial dose: 30 mg/d; maitenance dose: 30-60 mg/d. *E* | Other antihypertensive drugs (amlodipine, cardioselective beta-blockers, ACE inhibitors, diuretics). *E, L* |
| ***C08D*** | ***Selective calcium channel blockers with direct cardiac effects*** |  |  |  |  |
| C08DA01 | Verapamil  In lists: 3, 5 (A); 2, 6 (B) | 23; 2.39 [1.98-2.80]; 2.00 | May worsen constipation; risk of bradycardia | Immediate release tablets: initial dose 40 mg three times daily; sustained release tablets: initial dose 120 mg daily; oral controlled onset extended release: initial dose 100 mg/d. *M* | Other antihypertensive drugs (amlodipine, cardioselective beta-blockers, ACE inhibitors, diuretics). *E* |
| C08DB01 | Diltiazem  In lists: 3, 5 (A); 2, 6 (B) | 23; 2.57 [2.18-2.95]; 2.00 | May worsen constipation; risk of bradycardia | Reduce dose or increase dosing interval. *M* 60 mg three times daily. *E* |  |
| ***C10*** | ***Lipid modifiying agents*** |  |  |  |  |
| ***C10A*** | ***Lipid modifiying agents, plain*** |  |  |  |  |
| C10AD02 | Niacin (=Nicotinic acid)  In lists: 2 (A) | 22; 1.77 [1.28-2.26]; 1.00 | Moderate risk of side effects; ineffective for the treatment of dementia |  |  |
| **G** | **Genito-urinary system and sex hormones** |  |  |  |  |
| ***G03*** | ***Sex hormones and modulator of the genital system*** |  |  |  |  |
| ***G03C*** | ***Oestrogens*** |  |  |  |  |
| G03C | Oestrogen (oral)  In lists: 5 (A); 6 (B) | 21; 1.52 [1.21-1.83]; 1.00 | Evidence for carcinogenic potential (breast and endometrial cancer) and lack of cardioprotective effect in older women |  | Specific treatment for osteoporosis. *E* Local administration (i.e. vaginal application) considered safe and efficient. *E, B* |
| ***G04*** | ***Urologicals*** |  |  |  |  |
| ***G04B*** | ***Other urologicals, incl. antispasmodics*** |  |  |  |  |
| G04BD02 | Flavoxat  In lists: 5, 6 (B) | 16; 1.75 [1.22-2.28]; 1.00 | May decrease urinary flow, leading to urinary retention |  | Non-pharmacological treatment (pelvic floor exercises, physical and behavioural therapy). *E* |
| G04BD04 | Oxybutynine (non-sustained-release)  In lists: 1, 3, 4, 5 (A); 5, 6 (B) | 23; 1.43 [1.78-1.69]; 1.00 | Anticholinergic side effects (e.g. constipation, dry mouth, CNS side effects); ECG changes (prolonged QT) | Start immediate-release oxybutynin chloride in frail older adults with 2.5 mg orally 2 or 3 times daily. *M* | Non-pharmacological treatment (pelvic floor exercises, physical and behavioural therapy). *E* |
| G04BD04 | Oxybutynine (sustained-release)  In lists: 1, 3, 4, 5 (A); 5, 6 (B) | 23; 1.57 [1.16-1.97]; 1.00 | Anticholinergic side effects (e.g. constipation, dry mouth, CNS side effects); ECG changes (prolonged QT) |  | Non-pharmacological treatment (pelvic floor exercises, physical and behavioural therapy). *E* |
| G04BD07 | Tolterodine (non-sustained-release)  In lists: 1, 3, 4, 5 (A); 5, 6 (B) | 22; 1.59 [1.27-1.92]; 1.00 | Anticholinergic side effects (e.g. constipation, dry mouth, CNS side effects); ECG changes (prolonged QT) | 1 mg orally twice daily in cases of significantly impaired renal function. *M* | Non-pharmacological treatment (pelvic floor exercises, physical and behavioural therapy). *E* |
| G04BD07 | Tolterodine (sustained-release)  In lists: 1, 3, 5 (A); 5, 6 (B) | 22; 1.77 [1.32-2.23]; 1.00 | Anticholinergic side effects (e.g. constipation, dry mouth, CNS side effects); ECG changes (prolonged QT) | Use 2 mg orally once daily in cases of severe renal failure (CrCl 10-30 mL/min); avoid use if CrCl <10 mL/min. *M* | Non-pharmacological treatment (pelvic floor exercises, physical and behavioural therapy). *E* |
| G04BD08 | Solifenacin  In lists: 1, 3, 4, 5 (A); 5, 6 (B) | 21; 1.81 [1.34-2.28]; 1.00 | Anticholinergic side effects (e.g. constipation, dry mouth, CNS side effects); ECG changes (prolonged QT) | Dose reduction may be needed. *M* | Non-pharmacological treatment (pelvic floor exercises, physical and behavioural therapy). *E* |
| G04BD09 | Trospium  In lists: 5 (A); 5, 6 (B) | 18; 1.94 [1.42-2.47]; 2.00 | Anticholinergic side effects (e.g. constipation, dry mouth, CNS side effects) | CrCl <30 mL/min: 20 mg/d (immediate release); avoid the use of extended release trospium. *M*  In adults aged ≥75 years old, the dose frequency of trospium immediate release may be reduced to 20 mg/d. *M* | Non-pharmacological treatment (pelvic floor exercises, physical and behavioural therapy). *E* |
| G04BD10 | Darifenacin  In lists: 3, 5 (A); 5, 6 (B) | 14; 1.79 [2.27-2.30]; 2.00 | Higher incidence of antimuscarinic adverse events (e.g., dry mouth, constipation, dyspepsia, increased residual urine, dizziness) and urinary tract infection in persons aged 75 years and older compared with younger patients |  | Non-pharmacological treatment (pelvic floor exercises, physical and behavioural therapy). *E* |
| G04BD11 | Fesoterodin  In lists: 3, 5 (A); 5, 6 (B) | 14; 1.71 [1.24-2.19]; 1.50 | Higher incidence of antimuscarinic adverse events (e.g., dry mouth, constipation, dyspepsia, increased residual urine, dizziness) and urinary tract infection in persons aged 75 years and older compared with younger patients | CrCl <30 mL/min: maximum dose 4 mg/d. *M* | Non-pharmacological treatment (pelvic floor exercises, physical and behavioural therapy). *E* |
| ***G04C*** | ***Drug used in benign prostatic hypertrophy*** |  |  |  |  |
| G04CA03 | Terazosin  In lists: 4, 5 (A); 6 (B) | 21; 1.52 [1.25-1.80]; 1.00 | Higher risk of orthostatic hypotension, dry mouth, urinary incontinence/ impaired micturition, CNS side effects (e.g. vertigo, light-headedness, somnolence) and cerebrovascular and cardiovascular disease | Low initial dose, half of usual dose, taper in and out. *P* Initial dose: 1 mg at bedtime; up to 10 mg/d may be required. *E* | If used as antihypertensive, other antihypertensive agents: ACE inhibitors, beta-blockers, calcium antagonists, diuretics (exclude PIM). *E* |
| **J** | **Antiinfectives for systematic use** |  |  |  |  |
| ***J01*** | ***Antibacterial for systemic use*** |  |  |  |  |
| ***J01M*** | ***Quinolone antibacterials*** |  |  |  |  |
| J01MA01 | Ofloxacin  In lists: does not appear as PIM | 22; 2.23 [1.70-2.76]; 2.00 | Its half-life may be prolonged with elevated serum concentrations in older adults; increased risk of torsade de pointes and tendinitis or tendon rupture | Reduce dose and increase dosing interval if renal failure. *M* | Other antibiotics in accordance with sensitivity and resistance testing. *E* |
| ***J01X*** | ***Other antibacterials*** |  |  |  |  |
| J01XE01 | Nitrofurantoin (>1 week)  In lists: 1, 4, 5 (A) | 21; 2.00 [1.59-2.41]; 2.00 | Unfavourable risk/benefit ratio, particularly with long-term use (pulmonary side effects, liver damage, etc.); contraindicated if severe renal failure due to decreased excretion and increased risk of toxicity | 50-100 mg/8h; use shorter than one week. *E* | Other antibiotics in accordance with sensitivity and resistance testing. *E* |
| **M** | **Musculo-skeletal system** |  |  |  |  |
| ***M01*** | ***Anti-inflammatory and anti-rheumatic products*** |  |  |  |  |
| ***M01A*** | ***Anti-inflammatory and anti-rheumatic products, non-steroid (NSAID)*** |  |  |  |  |
| M01AA01 | Phenylbutazone  In lists: 1, 2, 4 (A); 5, 6 (B) | 19; 1.21 [1.01-1.41]; 1.00 | Very high risk of GI bleeding, ulceration, or perforation, which may be fatal;  risk of blood dyscrasia | Use for the shortest period possible. *P* The risk of bleeding may be reduced if combined with proton-pump inhibitors (use <8 weeks, low dose)*. E* | Paracetamol; ibuprofen (≤3 x 400 mg/d or for a period shorter than one week); naproxen (≤2 x 250 mg/d or for a period shorter than one week). *E* Opioids with lower risk of delirium (e.g., tilidine/naloxone, morphine^d^, oxycodone, buprenorphine, hydromorphone). *E, P* |
| M01AB01 | Indometacin  In lists: 1, 3, 4, 5 (A); 2, 5, 6 (B) | 23; 1.39 [1.08-1.70]; 1.00 | Very high risk of GI bleeding, ulceration, or perforation, which may be fatal; risk of CNS disturbances | Reduce dose reduction by 25%. *M* Use for the shortest period possible. *P* The risk of bleeding may be reduced if combined with proton-pump inhibitors (use <8 weeks, low dose). *E* | Paracetamol; ibuprofen (≤3 x 400 mg/d or for a period shorter than one week); naproxen (≤2 x 250 mg/d or for a period shorter than one week). *E* Opioids with lower risk of delirium (e.g., tilidine/naloxone, morphine^d^, oxycodone, buprenorphine, hydromorphone). *E, P* |
| M01AB05 | Diclofenac  In lists: 5 (A); 1, 2, 5, 6 (B) | 23; 2.00 [1.59-2.41]; 2.00 | Very high risk of GI bleeding, ulceration, or perforation, which may be fatal; cardiovascular contraindications | 50 mg/d; start using low dose; the risk of bleeding may be reduced if combined with proton-pump inhibitors (use <8 weeks, low dose). *E* | Paracetamol; ibuprofen (≤3 x 400 mg/d or for a period shorter than one week); naproxen (≤2 x 250 mg/d or for a period shorter than one week). *E* Opioids with lower risk of delirium (e.g., tilidine/naloxone, morphine^d^, oxycodone, buprenorphine, hydromorphone). *E, P* |
| M01AB11 | Acemetacin  In lists: 4 (A); 1, 2, 4, 5, 6 (B) | 16; 1.50 [1.22-1.78]; 1.50 | Very high risk of GI bleeding, ulceration, or perforation, which may be fatal | Use for the shortest period possible. *P* The risk of bleeding may be reduced if combined with proton-pump inhibitors (use <8 weeks, low dose). *E* | Paracetamol; ibuprofen (≤3 x 400 mg/d or for a period shorter than one week); naproxen (≤2 x 250 mg/d or for a period shorter than one week). *E* Opioids with lower risk of delirium (e.g., tilidine/naloxone, morphine^d^, oxycodone, buprenorphine, hydromorphone). *E, P* |
| M01AB15 | Ketorolac  In lists: 5 (A); 1, 2, 5, 6 (B) | 21; 1.76 [1.44-2.08]; 2.00 | Very high risk of GI bleeding, ulceration, or perforation, which may be fatal | Contraindicated in cases of advanced renal failure; oral dose not indicated as initial dose; recommended continuation dose after intravenous or intramuscular dosing is 10 mg every 4-6 hours, maximum 40 mg/d and for 5 days. *M* The risk of bleeding may be reduced if combined with proton-pump inhibitors (use <8 weeks, low dose). *E* | Paracetamol; ibuprofen (≤3 x 400 mg/d or for a period shorter than one week); naproxen (≤2 x 250 mg/d or for a period shorter than one week). *E* Opioids with lower risk of delirium (e.g., tilidine/naloxone, morphine^d^, oxycodone, buprenorphine, hydromorphone). *E, P* |
| M01AB16 | Aceclofenac  In lists: 1, 2, 5, 6 (B) | 20; 1.85 [1.50-2.20]; 2.00 | Very high risk of GI bleeding, ulceration, or perforation, which may be fatal; cardiovascular contraindications | Start using low dose; the risk of bleeding may be reduced if combined with proton-pump inhibitors (use <8 weeks, low dose). *E* | Paracetamol; ibuprofen (≤3 x 400 mg/d or for a period shorter than one week); naproxen (≤2 x 250 mg/d or for a period shorter than one week). *E* Opioids with lower risk of delirium (e.g., tilidine/naloxone, morphine^d^, oxycodone, buprenorphine, hydromorphone). *E, P* |
| M01AC01 | Piroxicam  In lists: 4, 5 (A); 1, 2, 5, 6 (B) | 22; 1.55 [1.28-1.81]; 1.50 | Very high risk of GI bleeding, ulceration, or perforation, which may be fatal | Doses >20 mg are associated with increased GI toxicity and ulceration, especially in older adults. *M* Use for the shortest period possible. *P* 10 mg/d; start with lower dose; the risk of bleeding may be reduced if combined with proton-pump inhibitors (use <8 weeks, low dose). *E* | Paracetamol; ibuprofen (≤3 x 400 mg/d or for a period shorter than one week); naproxen (≤2 x 250 mg/d or for a period shorter than one week). *E* Opioids with lower risk of delirium (e.g., tilidine/naloxone, morphine^d^, oxycodone, buprenorphine, hydromorphone). *E, P* |
| M01AC05 | Lornoxicam  In lists: 1, 2, 5, 6 (B) | 19; 1.74 [1.35-2.13]; 2.00 | Very high risk of GI bleeding, ulceration, or perforation, which may be fatal; cardiovascular contraindications | Use for the shortest period possible. *P* Start with lower dose; the risk of bleeding may be reduced if combined with proton-pump inhibitors (use <8 weeks, low dose). *E* | Paracetamol; ibuprofen (≤3 x 400 mg/d or for a period shorter than one week); naproxen (≤2 x 250 mg/d or for a period shorter than one week). *E* Opioids with lower risk of delirium (e.g., tilidine/naloxone, morphine^d^, oxycodone, buprenorphine, hydromorphone). *E, P* |
| M01AC06 | Meloxicam  In lists: 4, 5 (A); 1, 2, 5, 6 (B) | 23; 1.65 [1.34-1.96]; 2.00 | Very high risk of GI bleeding, ulceration, or perforation, which may be fatal | 11 mg/d; start with lower dose; the risk of bleeding may be reduced if combined with proton-pump inhibitors (use <8 weeks, low dose). *E* | Paracetamol; ibuprofen (≤3 x 400 mg/d or for a period shorter than one week); naproxen (≤2 x 250 mg/d or for a period shorter than one week). *E* Opioids with lower risk of delirium (e.g., tilidine/naloxone, morphine^d^, oxycodone, buprenorphine, hydromorphone). *E, P* |
| M01AE01 | Ibuprofen (>3 x 400 mg/d or for a period longer than one week)^c^  In lists: 5 (A); 5, 6 (B) | 21; 2.43 [1.98-2.87]; 2.00 | Risk of GI bleeding and increased risk of cardiovascular complications at higher doses (>1200 mg/d), especially in cases of previous cardiovascular disease | The risk of bleeding may be reduced if combined with proton-pump inhibitors (use <8 weeks, low dose). *E* | Paracetamol; ibuprofen (≤3 x 400 mg/d or for a period shorter than one week); naproxen (≤2 x 250 mg/d or for a period shorter than one week). *E* Opioids with lower risk of delirium (e.g., tilidine/naloxone, morphine^d^, oxycodone, buprenorphine, hydromorphone). *E, P* |
| M01AE02 | Naproxen (>2 x 250 mg/d or for a period longer than one week)^c^  In lists: 5 (A); 5, 6 (B) | 23; 2.04 [1.62-2.47]; 2.00 | Risk of GI bleeding | Reduce dose; start low–go slow in older adults; avoid if CrCl <30 mL/min. *M*  The risk of bleeding may be reduced if combined with proton-pump inhibitors (use <8 weeks, low dose). *E* | Paracetamol; ibuprofen (≤3 x 400 mg/d or for a period shorter than one week); naproxen (≤2 x 250 mg/d or for a period shorter than one week). *E* Opioids with lower risk of delirium (e.g., tilidine/naloxone, morphine^d^, oxycodone, buprenorphine, hydromorphone). *E, P* |
| M01AE03 | Ketoprofen  In lists: 4, 5 (A); 1, 2, 5, 6 (B) | 23; 1.87 [1.45-2.29]; 2.00 | Very high risk of GI bleeding, ulceration, or perforation, which may be fatal | Reduce dose if CrCl <20 mL/min; start with lower dose and use reduced maintenance dose in older adults. *M*  Use for the shortest period possible. *P* The risk of bleeding may be reduced if combined with proton-pump inhibitors (use <8 weeks, low dose). *E* | Paracetamol; ibuprofen (≤3 x 400 mg/d or for a period shorter than one week); naproxen (≤2 x 250 mg/d or for a period shorter than one week). *E* Opioids with lower risk of delirium (e.g., tilidine/naloxone, morphine^d^, oxycodone, buprenorphine, hydromorphone). *E, P* |
| M01AE09 | Flurbiprofen  In lists: 1, 2, 5, 6 (B) | 19; 1.84 [1.41-2.28]; 2.00 | Very high risk of GI bleeding, ulceration, or perforation, which may be fatal; cardiovascular contraindications | Start with lower dose; the risk of bleeding may be reduced if combined with proton-pump inhibitors (use <8 weeks, low dose). *E* | Paracetamol; ibuprofen (≤3 x 400 mg/d or for a period shorter than one week); naproxen (≤2 x 250 mg/d or for a period shorter than one week). *E* Opioids with lower risk of delirium (e.g., tilidine/naloxone, morphine^d^, oxycodone, buprenorphine, hydromorphone). *E, P* |
| M01AE17 | Dexketoprofen  In lists: 1, 2, 5, 6 (B) | 23; 1.91 [1.50-2.32]; 2.00 | Very high risk of GI bleeding, ulceration, or perforation, which may be fatal; cardiovascular contraindications | Start with lower dose, up to 50 mg/d in older adults; in postoperative pain: 50 mg/d in case of renal or hepatic failure, maximum dose 50 mg/8h; maximum length 48 hours; the risk of bleeding may be reduced if combined with proton-pump inhibitors (use <8 weeks, low dose). *E* | Paracetamol; ibuprofen (≤3 x 400 mg/d or for a period shorter than one week); naproxen (≤2 x 250 mg/d or for a period shorter than one week). *E* Opioids with lower risk of delirium (e.g., tilidine/naloxone, morphine^d^, oxycodone, buprenorphine, hydromorphone). *E, P* |
| M01AG01 | Mefenamic acid  In lists: 5 (A); 1, 2, 5, 6 (B) | 18; 1.72 [1.35-2.10]; 2.00 | Very high risk of GI bleeding, ulceration, or perforation, which may be fatal; cardiovascular contraindications | Start with lower dose; the risk of bleeding may be reduced if combined with proton-pump inhibitors (use <8 weeks, low dose). *E* | Paracetamol; ibuprofen (≤3 x 400 mg/d or for a period shorter than one week); naproxen (≤2 x 250 mg/d or for a period shorter than one week). *E* Opioids with lower risk of delirium (e.g., tilidine/naloxone, morphine^d^, oxycodone, buprenorphine, hydromorphone). *E, P* |
| M01AH01 | Celecoxib  In lists: 1, 2, 5, 6 (B) | 21; 1.67 [1.28-2.06]; 1.00 | Very high risk of GI bleeding, ulceration, or perforation, which may be fatal;  cardiovascular contraindications | The risk of bleeding may be reduced if combined with proton-pump inhibitors (use <8 weeks, low dose). *E* | Paracetamol; ibuprofen (≤3 x 400 mg/d or for a period shorter than one week); naproxen (≤2 x 250 mg/d or for a period shorter than one week). *E* Opioids with lower risk of delirium (e.g., tilidine/naloxone, morphine^d^, oxycodone, buprenorphine, hydromorphone). *E, P* |
| M01AH05 | Etoricoxib  In lists: 4 (A); 1, 2, 5, 6 (B) | 22; 1.73 [1.34-2.12]; 1.50 | Very high risk of GI bleeding, ulceration, or perforation, which may be fatal;  cardiovascular contraindications | Shortest possible duration of therapy. *P* Start with lower dose; the risk of bleeding may be reduced if combined with proton-pump inhibitors (use <8 weeks, low dose). *E* | Paracetamol; ibuprofen (≤3 x 400 mg/d or for a period shorter than one week); naproxen (≤2 x 250 mg/d or for a period shorter than one week). *E* Opioids with lower risk of delirium (e.g., tilidine/naloxone, morphine^d^, oxycodone, buprenorphine, hydromorphone). *E, P* |
| M01AX01 | Nabumetone  In lists: 5 (A); 1, 2, 5, 6 (B) | 20; 1.70 [1.33-2.08]; 1.50 | Very high risk of GI bleeding, ulceration, or perforation, which may be fatal; cardiovascular contraindications | Adjust dose in cases of moderate or severe renal failure; maximum starting dose should not exceed 750 mg or 500 mg/d, to a maximum of 1500 mg and 1000 mg/d; older adults should receive single daily doses of 1000mg; dose reduction recommended, consider low starting dose. *M* The risk of bleeding may be reduced if combined with proton-pump inhibitors (use <8 weeks, low dose). *E* | Paracetamol; ibuprofen (≤3 x 400 mg/d or for a period shorter than one week); naproxen (≤2 x 250 mg/d or for a period shorter than one week). *E* Opioids with lower risk of delirium (e.g., tilidine/naloxone, morphine^d^, oxycodone, buprenorphine, hydromorphone). *E, P* |
| ***M03*** | ***Muscle relaxants*** |  |  |  |  |
| ***M03B*** | ***Muscle relaxants, centrally acting agents*** |  |  |  |  |
| M03BA02 | Carisoprodol  In lists: 5 (A); 5 (B) | 13; 1.62 [1.15-2.08]; 1.00 | Risk of anticholinergic and CNS side effects including orthostatic hypotension, falls, sedation, weakness, confusion, amnesia |  |  |
| M03BA03 | Methocarbamol  In lists: 1, 2, 5 (A) | 13; 1.62 [1.15-2.08]; 1.00 | Risk of anticholinergic and CNS side effects including orthostatic hypotension, falls, sedation, weakness, confusion, amnesia |  | Rehabilitation; botulinum toxin. *E* |
| M03BC01 | Orphenadrine  In lists: 3, 5 (A); 5 (B) | 16; 1.38 [1.11-1.64]; 1.00 | Risk of anticholinergic and CNS side effects including orthostatic hypotension, falls, sedation, weakness, confusion, amnesia |  | Rehabilitation; botulinum toxin. *E* |
| M03BX01 | Baclofen  In lists: 1, 3, 4 (A) | 22; 2.14 [1.72-2.55]; 2.00 | Risk of anticholinergic and CNS side effects including orthostatic hypotension, falls, sedation, weakness, confusion, amnesia | Dose reductions may be required in cases of renal failure; start low–go slow in older adults. *M* Start with 5 mg 2-3 times daily and increase gradually as needed; maximum dose: 10 mg 3 times daily. *E* | Rehabilitation; botulinum toxin. *E* |
| M03BX02 | Tizanidine  In lists: 3 (A), 5 (B) | 18; 1.94 [1.37-2.52]; 2.00 | Risk of anticholinergic and CNS side effects including orthostatic hypotension, falls, sedation, weakness, confusion, amnesia | Dose reductions may be required in cases of renal failure. *M* | Rehabilitation; botulinum toxin. *E* |
| M03BX07 | Tetrazepam  In lists: 1, 4 (A) | 15; 1.80 [1.37-2.23]; 2.00 | Risk of anticholinergic and CNS side effects including orthostatic hypotension, falls, sedation, weakness, confusion, amnesia | Cautious dosing in cases of renal failure. *M* Conservative dosing for older adults. *M, E* | Rehabilitation; botulinum toxin. *E* |
| M03BX08 | Cyclobenzaprine  In lists: 2, 5 (A); 5 (B) | 16; 1.69 [1.22-2.15]; 1.00 | Risk of anticholinergic and CNS side effects including orthostatic hypotension, falls, sedation, weakness, confusion, amnesia | Start low–go slow. *M* |  |
| ***M04*** | ***Antigout preparations*** |  |  |  |  |
| ***M04A*** | ***Antigout preparations*** |  |  |  |  |
| M04AC01 | Colchicin  In lists: 6 (B) | 18; 2.11 [1.66-2.56]; 2.00 | Higher risk of toxicity in older adults, particularly in cases of existing renal, GI or cardiac disease | Reduce dose by 50% in older adults (>70 years old). *M* Reduce dose in cases of renal failure. *E, M* | Ibuprofen (≤3 x 400 mg/d or for a period shorter than one week); naproxen (≤2 x 250 mg/d or for a period shorter than one week). *E* |
| ***M05*** | ***Drugs for treatment of bone diseases*** |  |  |  |  |
| ***M05B*** | ***Drugs affecting bone structure and mineralization*** |  |  |  |  |
| M05BX03 | Strontium ranelate  In lists: does not appear as PIM | 18; 1.72 [1.35-2.10]; 2.00 | Higher risk of venous thromboembolism in persons who are temporarily or permanently immobilised. Evaluate the need for continued therapy for patients over 80 years old with increased risk of venous thromboembolism | Avoid in cases of severe renal failure (CrCl <30 mL/min). *M* | Bisphosphonates, Vitamin D. *E* |
| ***M09*** | ***Other drugs for disorders of the musculo-skeletal system*** |  |  |  |  |
| ***M09A*** | ***Other drugs for disorders of the musculo-skeletal system*** |  |  |  |  |
| M09AA | Quinine and derivatives  In lists: does not appear as PIM | 15; 2.13 [1.44-2.82]; 2.00 | Risk of cardiac and idiosyncratic adverse effects | Adjust dose in cases of renal failure. *M* |  |
| **N** | **Nervous system** |  |  |  |  |
| ***N02*** | ***Analgesics*** |  |  |  |  |
| ***N02A*** | ***Opioids*** |  |  |  |  |
| N02AB02 | Pethidine (=Meperidine)  In lists: 4, 5 (A); 2, 6 (B) | 22; 1.50 [1.24-1.77]; 1.00 | Risk of falls, fractures, confusion, dependency and withdrawal syndrome | Start low–go slow. *M, P* Use for the shortest period possible. *P* 50 mg every 4-6 hours. *E*  Use 75% of the normal dose at the usual intervals in cases of moderate renal failure (GFR 10-50 mL/min); use 50% of the normal dose at the usual intervals in cases of severe renal failure (GFR <10 mL/min). *M* | Paracetamol; ibuprofen (≤3 x 400 mg/d or for a period shorter than one week); naproxen (≤2 x 250 mg/d or for a period shorter than one week). *E* Opioids with lower risk of delirium (e.g., tilidine/naloxone, morphine^d^, oxycodone, buprenorphine, hydromorphone). *E, P* |
| N02AD01 | Pentazocine  In lists: 5 (A); 2, 6 (B) | 18; 1.28 [1.05-1.51]; 1.00 | Risk of delirium and agitation | For patients with GFR between 10 and 50 mL/min the dose should be reduced by 25% and for patients with GFR less than 10 mL/min, the dose should be decreased by 50%. *M* | Paracetamol; ibuprofen (≤3 x 400 mg/d or for a period shorter than one week); naproxen (≤2 x 250 mg/d or for a period shorter than one week). *E* Opioids with lower risk of delirium (e.g., tilidine/naloxone, morphine^d^, oxycodone, buprenorphine, hydromorphone). *E, P* |
| N02AX02 | Tramadol (sustained-release)  In lists: 5, 6 (B) | 23; 1.83 [1.44-2.21]; 2.00 | More adverse effects in older adults; CNS side effects such as confusion, vertigo and nausea | Start low–go slow.  Not to be used in cases of severe renal failure. *E, M* | Paracetamol; ibuprofen (≤3 x 400 mg/d or for a period shorter than one week); naproxen (≤2 x 250 mg/d or for a period shorter than one week). *E* Opioids with lower risk of delirium (e.g., tilidine/naloxone, morphine^d^, oxycodone, buprenorphine, hydromorphone). *E, P* |
| N02AX02 | Tramadol (non-sustained-release)  In lists: 5, 6 (B) | 21; 2.33 [1.77-2.90]; 2.00 | More adverse effects in older adults; CNS side effects such as confusion, vertigo and nausea | Start low–go slow; in persons older than 75 years, daily doses over 300 mg are not recommended. *M* Start with 12.5 mg/8h and progressive increases of 12.5 mg/8h; maximum 100mg/8h. *E* Reduce dose and extend the dosing interval for patients with severe renal failure. *M* | Paracetamol; ibuprofen (≤3 x 400 mg/d or for a period shorter than one week); naproxen (≤2 x 250 mg/d or for a period shorter than one week). *E* Opioids with lower risk of delirium (e.g., tilidine/naloxone, morphine^d^, oxycodone, buprenorphine, hydromorphone). *E, P* |
| N07BC02 | Methadone  In lists: 6 (B) | 22; 1.82 [1.47-2.17]; 2.00 | Very long-acting especially in the elderly | Lowest possible dose. *E* Start low–go slow.  Lower initial methadone dose with longer dosing intervals are recommended, along with a slower dose titration for patients with renal failure. *M* | Paracetamol; ibuprofen (≤3 x 400 mg/d or for a period shorter than one week); naproxen (≤2 x 250 mg/d or for a period shorter than one week). *E* Opioids with lower risk of delirium (e.g., tilidine/naloxone, morphine^d^, oxycodone, buprenorphine, hydromorphone). *E, P* |
| ***N02B*** | ***Other analgesics and antipyretics*** |  |  |  |  |
| N02BA01 | Acetylsalicylic acid (>325 mg)  In lists: 3, 5 (A); 2, 5, 6 (B) | 23; 1.83 [1.33-2.33]; 1.00 | May exacerbate existing GI ulcers or produce new GI ulcers; increased risk of bleeding due to prolongated clotting time, elevation of INR values or inhibition of platelet aggregation |  | Paracetamol; ibuprofen (≤3 x 400 mg/d or for a period shorter than one week); naproxen (≤2 x 250 mg/d or for a period shorter than one week). *E* Opioids with lower risk of delirium (e.g., tilidine/naloxone, morphine^d^, oxycodone, buprenorphine, hydromorphone). *E, P* |
| ***N02C*** | ***Antimigraine preparations*** |  |  |  |  |
| N02CA02 | Ergotamine  In lists: 4 (A) | 20; 1.55 [1.08-2.02]; 1.00 | Unfavourable risk/benefit profile |  | Paracetamol; ibuprofen (≤3 x 400 mg/d or for a period shorter than one week); naproxen (≤2 x 250 mg/d or for a period shorter than one week); non-pharmacological treatment (silence, rest, darkness). *E* |
| N02CC | Triptanes (e.g. Sumatriptan, Eletriptan, Naratriptan, Zolmitriptan)  In lists: does not appear as PIM | 23; 2.13 [1.78-2.48]; 2.00 | Safety and efficacy in older adults have not been established Naratriptan and sumatriptan use for older adults has an increased risk of decreased hepatic function and reduced clearance due to renal dysfunction, higher risk for coronary artery disease, and increases in blood pressure *M* | Start low–go slow. *M* Eletriptan Hydrobromide: initial dose of 20 mg, may be repeated after 2 hours; usual dose of 20-40 mg; maximum dose: 40 mg for older adults. *M*  Naratriptan: contraindicated in cases of severe renal failure (CrCl <15 mL/min). In cases of mild to moderate renal failure, a lower starting dose should be considered and the maximum dose is 2.5 mg/d. *M* | Paracetamol; ibuprofen (≤3 x 400 mg/d or for a period shorter than one week); naproxen (≤2 x 250 mg/d or for a period shorter than one week); non-pharmacological treatment (silence, rest, darkness). *E* |
| ***N03*** | ***Antiepileptics*** |  |  |  |  |
| ***N03A*** | ***Antiepileptics*** |  |  |  |  |
| N03AA02 | Phenobarbital  In lists: 4, 5 (A); 5 (B) | 22; 1.50 [1.24-1.77]; 1.00 | Risk of sedation, paradoxical excitation | Use lowest possible dose. *E, M* Start at the lowest possible dose, taper down to half of the usual dose. *P*  Administer every 12-16 hours in cases of severe renal failure (GFR <10 ml/min). Avoid longer acting barbiturates for long term use in cases of renal failure. Decrease doses significantly for short-term therapy. *M* | Levetiracetam^d^; gabapentin^d^; lamotrigine^d^; valproic acid^d^. *E* |
| N03AB02 | Phenytoin  In lists: 3 (A); 5 (B) | 22; 2.18 [1.76-2.61]; 2.00 | Narrow therapeutic window; increased risk of toxicity in older adults (e.g. CNS and hematologic toxicity) | Lower doses or less frequent dosing may be necessary for older adults due to reduced clearance, hypoalbuminemia or renal disease*. M* Start with 3 mg/kg/day, in divided doses, adjust the dosage according to serum hydantoin concentrations and patient response; use as a guide the plasma levels, increase the dose in increments of 50-100 mg/d every 5-7 days to achieve an effective dose; the usual maintenance dose is 300-500 mg/d or 4-7 mg / kg / d in 2 doses. *E* | Levetiracetam^d^; gabapentin^d^; lamotrigine^d^; valproic acid^d^. *E* |
| N03AE01 | Clonazepam  In lists: 3, 5 (A); 5 (B) | 23; 1.70 [1.45-1.94]; 2.00 | Risk of falls, paradoxical reactions | Start low–go slow; 0.5 mg/d. *E* | Levetiracetam^d^; gabapentin^d^; lamotrigine^d^; valproic acid^d^. *E* |
| N03AF01 | Carbamazepine  In lists: 5 (A); 5 (B) | 23; 2.17 [1.71-2.64]; 2.00 | Increased risk of SIADH-like syndrome; adverse events like carbamazepine-induced confusion and agitation, atrioventricular block and bradycardia | Adjust dose to the response and serum concentration. *E* | Levetiracetam^d^; gabapentin^d^; lamotrigine^d^; valproic acid^d^. *E* |
| N03AX11 | Topiramate  In lists: 5 (B) | 19; 2.53 [2.12-2.93]; 2.00 | Risk of cognitive-related dysfunction (e.g., confusion, psychomotor slowing) | Dosage adjustment may be indicated in older adults to the extent renal function is reduced. In cases of evident impaired renal function (CrCl <70 mL/min/1.73 m), use one-half the usual dose. *M* Use initial dose of 25 mg/d and increase 25 mg/d weekly up to 100-200 mg/d. *E* | Levetiracetam^d^; gabapentin^d^; lamotrigine^d^; valproic acid^d^. *E* |
| ***N04*** | ***Antiparkinson drugs*** |  |  |  |  |
| ***N04A*** | ***Anticholinergic agents*** |  |  |  |  |
| N04AA01 | Trihexyphenidyl  In lists: 1, 5 (A); 2, 5, 6 (B) | 17; 1.53 [1.08-1.98]; 1.00 | Risk of anticholinergic and CNS side effects including orthostatic hypotension, falls, sedation, weakness, confusion, amnesia | Start low–go slow. *M* | Levodopa; carbidopa-levodopa; benserazide levodopa; irreversible inhibitor of monoamine oxidase as rasagiline. *E* |
| N04AA02 | Biperiden  In lists: 1, 3 (A); 2, 6 (B) | 20; 1.50 [1.78-1.82]; 1.00 | Risk of anticholinergic and CNS side effects including orthostatic hypotension, falls, sedation, weakness, confusion, amnesia |  | Levodopa; carbidopa-levodopa; benserazide levodopa; irreversible inhibitor of monoamine oxidase as rasagiline. *E* |
| N04AA12 | Tropatepin  In lists: 1 (A); 2, 6 (B) | 15; 1.40 [1.05-1.75]; 1.00 | Risk of anticholinergic and CNS side effects including orthostatic hypotension, falls, sedation, weakness, confusion, amnesia |  | Levodopa; carbidopa-levodopa; benserazide levodopa; irreversible inhibitor of monoamine oxidase as rasagiline. *E* |
| N04AC01 | Benzatropine  In lists: 2, 6 (B) | 14; 1.14 [0.93-1.35]; 1.00 | Risk of anticholinergic and CNS side effects including orthostatic hypotension, falls, sedation, weakness, confusion, amnesia | Start low–go slow. *M* | Levodopa; carbidopa-levodopa; benserazide levodopa; irreversible inhibitor of monoamine oxidase as rasagiline. *E* |
| ***N04B*** | ***Dopaminergic agents*** |  |  |  |  |
| N04BB01 | Amantadine  In lists: does not appear as PIM | 20; 1.70 [1.39-2.00]; 2.00 | Risk of anticholinergic and CNS side effects including orthostatic hypotension, falls, sedation, weakness, confusion, amnesia | Start with 100 mg/d in 2 divided daily doses. *E* | Levodopa; carbidopa-levodopa; benserazide levodopa; irreversible inhibitor of monoamine oxidase as rasagiline. *E* |
| N04BC01 | Bromocriptine  In lists: 3 (A); 6 (B) | 22; 1.86 [1.38-2.34]; 1.50 | Risk of CNS side effects |  | Levodopa; carbidopa-levodopa; benserazide levodopa; irreversible inhibitor of monoamine oxidase as rasagiline. *E* |
| N04BC02 | Pergolide  In lists: 6 (B) | 16; 1.88 [1.45-2.30]; 2.00 | Adverse events include dyskinesia, dizziness, hallucinations, dystonia, confusion, somnolence, insomnia, anxiety, nausea |  | Levodopa; carbidopa-levodopa; benserazide levodopa; irreversible inhibitor of monoamine oxidase as rasagiline. *E* |
| N04BC03 | Dihydroergocryptine  In lists: 1, 4 (A); 6 (B) | 13; 2.15 [1.42-2.89]; 2.00 | Unfavourable risk/benefit profile |  | Levodopa; carbidopa-levodopa; benserazide levodopa; irreversible inhibitor of monoamine oxidase as rasagiline. *E* |
| N04BC04 | Ropinirole^c^  In lists: 6 (B) | 17; 2.47 [1.92-3.02]; 2.00 | Risk of orthostatic hypotension, hallucinations, confusion, somnolence, nausea | Start with three intakes of 0.25 mg per day, increase gradually by 0.25 mg per intake each week for four weeks, up to 3 mg/d. Afterwards the dose may be increased weekly by 1.5 mg/d up to 24 mg/d. *E* | Levodopa; carbidopa-levodopa; benserazide levodopa; irreversible inhibitor of monoamine oxidase as rasagiline. *E* |
| N04BC05 | Pramipexole^c^  In lists: 6 (A) | 19; 2.32 [1.86-2.77]; 2.00 | Side effects include orthostatic hypotension, GI tract symptoms, hallucinations, confusion, insomnia, peripheral oedema | Reduce dose in cases of moderate to severe renal failure. *M* Start with three intakes of 0.125 per day, increase gradually by 0.125 mg per intake every five to seven days, up to 1.5 to 4.5 mg. *E* | Levodopa; carbidopa-levodopa; benserazide levodopa; irreversible inhibitor of monoamine oxidase as rasagiline. *E* |
| N04BC06 | Cabergoline^c^  In lists: 3 (A); 6 (B) | 18; 1.78 [1.25-2.31]; 1.50 | CNS side effects |  | Levodopa; carbidopa-levodopa; benserazide levodopa; irreversible inhibitor of monoamine oxidase as rasagiline. *E* |
| N04BC08 | Piribedil  In lists: 1 (A); 6 (B) | 11; 1.73 [1.29-2.16]; 2.00 | Risk of orthostatic hypotension and falls |  | Levodopa; carbidopa-levodopa; benserazide levodopa; irreversible inhibitor of monoamine oxidase as rasagiline. *E* |
| N04BC09 | Rotigotine  In lists: 6 (B) | 15; 2.33 [1.68-2.98]; 2.00 | Side effects include orthostatic hypotension, headache, nausea, fatigue, sleep disorder, sudden onset of sleep, somnolence | One patch per day, usually started at 2 mg/24h and titrated weekly by increasing the patch size in increments of 2 mg/24h, up to 6 mg/24h; do not stop the treatment abruptly: sudden withdrawal may produce a syndrome resembling neuroleptic malignant syndrome or akinetic crisis. *E* | Levodopa; carbidopa-levodopa; benserazide levodopa; irreversible inhibitor of monoamine oxidase as rasagiline. *E* |
| N04BD01 | Selegiline  In lists: 3 (A) | 21; 2.29 [1.78-2.79]; 2.00 | Increased risk of orthostatic hypotension and dizziness | Do not use at doses >10 mg/d; 6mg/24h patch recommended; increase dose cautiously, paying attention to changes in orthostatic blood pressure. *E* | Levodopa; carbidopa-levodopa; benserazide levodopa; irreversible inhibitor of monoamine oxidase as rasagiline. *E* |
| ***N05*** | ***Psycholeptics*** |  |  |  |  |
| ***N05A*** | ***Antipsychotics*** |  |  |  |  |
| N05AA01 | Chlorpromazine  In lists: 1, 5 (A); 2, 5, 6 (B) | 21; 1.38 [1.11-1.65]; 1.00 | Muscarinic-blocking drug; risk of orthostatic hypotension and falls; may lower seizure thresholds in patients with seizures or epilepsy | Start low–go slow; use one-third to one-half the normal adult dose for debilitated older adults; use maintenance doses of 300 mg or less; doses greater than 1 gram do not usually offer any benefit, but may be responsible for an increased incidence of adverse effects. *M* | Non-pharmacological treatment; risperidone (<6 weeks), olanzapine (<10 mg/d), haloperidol (<2 mg single dose; <5mg/d); quetiapine^d^. *E* |
| N05AA02 | Levomepromazine  In lists: 1, 3, 4 (A); 5, 6 (B) | 22; 1.36 [1.15-1.58]; 1.00 | Anticholinergic and extrapyramidal side effects (tardive dyskinesia); parkinsonism; hypotonia; sedation; risk of falling; increased mortality in persons with dementia | Administer cautiously in cases of renal failure; start with doses of 5 to 10 mg in geriatric patients. *M* | Non-pharmacological treatment; risperidone (<6 weeks), olanzapine (<10 mg/d), haloperidol (<2 mg single dose; <5mg/d); quetiapine^d^. *E* |
| N05AA04 N05BA05 | Clorazepate-Acepromazine  In lists: 1 (A); 6 (B) | 14; 1.57 [1.08-2.06]; 1.00 | Protracted activity; risk of adverse effects such as drowsiness and falls |  | Non-pharmacological treatment; antidepressant with anxiolytic profile (SSRI^e^). *E* |
| N05AA06 | Cyamemazine  In lists: 1 (A); 5, 6 (B) | 12; 1.58 [1.08-2.09]; 1.00 | Muscarinic-blocking drug |  | Non-pharmacological treatment; risperidone (<6 weeks), olanzapine (<10 mg/d), haloperidol (<2 mg single dose; <5mg/d); quetiapine^d^. *E* |
| N05AB02 | Fluphenazine  In lists: 1, 4, 5 (A); 5, 6 (B) | 21; 1.43 [1.09-1.77]; 1.00 | Anticholinergic and extrapyramidal side effects (tardive dyskinesia); parkinsonism; hypotonia; sedation; risk of falling; increased mortality in persons with dementia | Start with oral dose of 1-2.5 mg/day. *M* | Non-pharmacological treatment; risperidone (<6 weeks), olanzapine (<10 mg/d), haloperidol (<2 mg single dose; <5mg/d); quetiapine^d^. *E* |
| N05AB03 | Perphenazine  In lists: 1, 3, 4, 5 (A); 5, 6 (B) | 20; 1.40 [1.05-1.75]; 1.00 | Anticholinergic and extrapyramidal side effects (tardive dyskinesia); parkinsonism; hypotonia; sedation; risk of falling; increased mortality in persons with dementia | Start low–go slow; use one-third to one-half the usual adult dose. *M* | Non-pharmacological treatment; risperidone (<6 weeks), olanzapine (<10 mg/d), haloperidol (<2 mg single dose; <5mg/d); quetiapine^d^. *E* |
| N05AB04 | Prochlorperazine  In lists: 3, 5 (A); 5, 6 (B) | 17; 1.47 [1.10-1.84]; 1.00 | Risk of anticholinergic side effects, sedation, falls, QTc-prolongation | Reduce dose; start low–go slow. *E, M* | Non-pharmacological treatment; risperidone (<6 weeks), olanzapine (<10 mg/d), haloperidol (<2 mg single dose; <5mg/d); quetiapine^d^. *E* |
| N05AB06 | Trifluoperazine  In lists: 5 (A); 5, 6 (B) | 15; 1.80 [1.37-2.23]; 2.00 | Risk of hypotension and neuromuscular reactions | Start low go slow. *M* | Non-pharmacological treatment; risperidone (<6 weeks), olanzapine (<10 mg/d), haloperidol (<2 mg single dose; <5mg/d); quetiapine^d^. *E* |
| N05AC01 | Propericiazine (=Periciazine)  In lists: 1, 3 (A); 5, 6 (B) | 14; 1.79 [1.32-2.25]; 2.00 | Anticholinergic and extrapyramidal side effects (tardive dyskinesia); parkinsonism; hypotonia; sedation; risk of falling; increased mortality in persons with dementia |  | Non-pharmacological treatment; risperidone (<6 weeks), olanzapine (<10 mg/d), haloperidol (<2 mg single dose; <5mg/d); quetiapine^d^. *E* |
| N05AC02 | Thioridazine  In lists: 4, 5 (A); 5, 6 (B) | 19; 1.37 [1.08-1.65]; 1.00 | Anticholinergic and extrapyramidal side effects (tardive dyskinesia); parkinsonism; hypotonia; sedation; risk of falling; increased mortality in persons with dementia | Reduce dose. *M* | Non-pharmacological treatment; risperidone (<6 weeks), olanzapine (<10 mg/d), haloperidol (<2 mg single dose; <5mg/d); quetiapine^d^. *E* |
| N05AC04 | Pipotiazine  In lists: 1 (A); 5, 6 (B) | 14; 1.50 [1.06-1.94]; 1.00 | Muscarinic-blocking drug | Reduce dose; start with doses of less than 25 mg. *M* | Non-pharmacological treatment; risperidone (<6 weeks), olanzapine (<10 mg/d), haloperidol (<2 mg single dose; <5mg/d); quetiapine^d^. *E* |
| N05AD01 | Haloperidol (>2 mg single dose; >5mg/d)  In lists: 4, 5 (A); 5, 6 (B) | 22; 1.59 [1.33-1.85]; 2.00 | Anticholinergic and extrapyramidal side effects (tardive dyskinesia); parkinsonism; hypotonia; sedation; risk of falling; increased mortality in persons with dementia | Use oral doses of 0.75-1.5 mg; use for the shortest period possible. *E* | Non-pharmacological treatment; risperidone (<6 weeks), olanzapine (<10 mg/d), haloperidol (<2 mg single dose; <5mg/d); quetiapine^d^. *E* |
| N05AD08 | Droperidol  In lists: 5, 6 (B) | 15; 1.73 [1.20-2.27]; 1.00 | Anticholinergic and extrapyramidal side effects (tardive dyskinesia); parkinsonism; hypotonia; sedation; risk of falling; increased mortality in persons with dementia | Reduce dose in cases of renal failure and in older adults. *M* | Non-pharmacological treatment; risperidone (<6 weeks), olanzapine (<10 mg/d), haloperidol (<2 mg single dose; <5mg/d); quetiapine^d^. *E* |
| N05AE03 | Sertindole  In lists: 3 (A); 5, 6 (B) | 16; 1.63 [1.20-2.05]; 1.00 | Risk of hypotension, falls, QTc-prolongation | 10 mg/d. *E* | Non-pharmacological treatment; risperidone (<6 weeks), olanzapine (<10 mg/d), haloperidol (<2 mg single dose; <5mg/d); quetiapine^d^. *E* |
| N05AE04 | Ziprasidone  In lists: 5, 6 (B) | 16; 2.13 [1.51-2.74]; 2.00 | Risk of QTc-prolongation, torsades de pointes, sedation, insomnia and orthostatic hypotension. Not approved for the treatment of dementia-related psychosis. Risk of increased mortality, increased with higher doses, when used for behavioural problems in dementia may be similar to the risk for risperidone | Starting dose 20 mg/d. *E* | Non-pharmacological treatment; risperidone (<6 weeks), olanzapine (<10 mg/d), haloperidol (<2 mg single dose; <5mg/d); quetiapine^d^. *E* |
| N05AF01 | Flupentixole  In lists: 3 (A); 5, 6 (B) | 17; 1.71 [1.27-2.14]; 2.00 | Adverse effects like tiredness, dizziness, QTc-prolongation | Dose adjustment may be required. *M* | Non-pharmacological treatment; risperidone (<6 weeks), olanzapine (<10 mg/d), haloperidol (<2 mg single dose; <5mg/d); quetiapine^d^. *E* |
| N05AF03 | Chlorprothixen  In lists: 3 (A); 5, 6 (B) | 15; 1.87 [1.24-2.49]; 2.00 | Lower seizure threshold | Start low–go slow. *M* | Non-pharmacological treatment; risperidone (<6 weeks), olanzapine (<10 mg/d), haloperidol (<2 mg single dose; <5mg/d); quetiapine^d^. *E* |
| N05AF05 | Zuclopenthixol  In lists: 3 (A); 5, 6 (B) | 12; 1.50 [1.07-1.93]; 1.00 | Risk of hypotension, falls, extrapyramidal effects, QT-prolongation | Use low oral doses of 2.5-5 mg/d. *M* | Non-pharmacological treatment; risperidone (<6 weeks), olanzapine (<10 mg/d), haloperidol (<2 mg single dose; <5mg/d); quetiapine^d^. *E* |
| N05AG02 | Pimozide  In lists: 5, 6 (B) | 14; 1.57 [1.27-1.87]; 2.00 | Anticholinergic and extrapyramidal side effects (tardive dyskinesia); parkinsonism; hypotonia; sedation; risk of falling; increased mortality and risk of cerebrovascular accident in persons with dementia. More rarely: neuroleptic malignant syndrome and QT-prolongation | Recommended initial dose of 1 mg/d. *E, M* | Non-pharmacological treatment; risperidone (<6 weeks), olanzapine (<10 mg/d), haloperidol (<2 mg single dose; <5mg/d); quetiapine^d^. *E* |
| N05AH02 | Clozapine  In lists: 3, 4, 5 (A); 5, 6 (B) | 22; 1.55 [1.28-1.81]; 1.50 | Anticholinergic and extrapyramidal side effects (tardive dyskinesia); parkinsonism; hypotonia; sedation; risk of falling; increased mortality in persons with dementia; increased risk of agranulocytosis and myocarditis | Start with 12.5 mg/d. *E* Start low–go slow; reduce dose in cases of significant renal failure. *M* | Non-pharmacological treatment; risperidone (<6 weeks), olanzapine (<10 mg/d), haloperidol (<2 mg single dose; <5mg/d); quetiapine^d^. *E* |
| N05AH03 | Olanzapine (>10 mg/d)  In lists: 4, 5 (A); 5, 6 (B) | 22; 1.64 [1.29-1.99]; 1.50 | Anticholinergic and extrapyramidal side effects (tardive dyskinesia); parkinsonism; hypotonia; sedation; risk of falling; increased mortality in persons with dementia |  | Non-pharmacological treatment; risperidone (<6 weeks), olanzapine (<10 mg/d), haloperidol (<2 mg single dose; <5mg/d); quetiapine^d^. *E* |
| N05AN01 | Lithium  In lists: 3 (A); 5, 6 (B) | 22; 2.27 [1.80-2.75]; 2.00 | Narrow therapeutic window; cumulation in renal failure | 300-600 mg/d. *E* Start low–go slow; it may be necessary to decrease dosage by as much as 50% in older adults to compensate for reduced clearance; dose reduction in cases of renal failure: GFR 10-50 ml/min, 50-75% of the usual dose; GFR <10 ml/min, 25-50% of the usual dose given at the normal dosage interval. *M, E* | Non-pharmacological treatment; SSRI^e^, mirtazapine^d^, trazodone. *E* |
| N05AX08 | Risperidone (>6 weeks)  In lists: 5 (A); 5, 6 (B) | 20; 2.45 [1.96-2.94]; 2.00 | Problematic risk-benefit profile for the treatment of behavioural symptoms of dementia; increased mortality, with higher dose, in patients with dementia | Use the lowest dose required (0.5-1.5 mg/d) for the shortest time period necessary. *E* For geriatric patients or in cases of severe renal failure (CrCl <30 mL/min), start with 0.5 mg twice daily; increase doses by 0.5 mg twice daily; increases above 1.5 mg twice daily should be done at intervals of at least 1 week; slower titration may be necessary. For geriatric patients, if once-daily dosing desired, initiate and titrate on a twice-daily regimen for 2 to 3 days to achieve target dose and switch to once-daily dosing thereafter. *M* | Non-pharmacological treatment; risperidone (<6 weeks), olanzapine (<10 mg/d), haloperidol (<2 mg single dose; <5mg/d); quetiapine^d^. *E* |
| N05AX12 | Aripiprazole  In lists: 5 (A); 5, 6 (B) | 16; 2.60 [1.46-2.66]; 2.00 | Risk of increased mortality when used for behavioural problems in dementia | Use the lowest dose required (7-12mg/d) for the shortest time period necessary. *E* | Non-pharmacological treatment; risperidone (<6 weeks), olanzapine (<10 mg/d), haloperidol (<2 mg single dose; <5mg/d); quetiapine^d^. *E* |
| ***N05B*** | ***Anxiolytics*** |  |  |  |  |
| N05BA01 | Diazepam  In lists: 1, 4, 5 (A); 2, 5, 6 (B) | 23; 1.61 [1.32-1.89]; 2.00 | Risk of falling with hip fracture; prolonged reaction times; psychiatric reactions (can also be paradoxical, e.g. agitation, irritability, hallucinations, psychosis); cognitive impairment; depression | Use the lowest possible dose, up to half of the usual dose, taper in and out, shortest possible duration of treatment. *P, M U*se initial oral dose of 2-2.5 mg once a day to twice a day. *M* | Non-pharmacological treatment; low doses of short-acting benzodiazepines such as lormetazepam (≤0.5 mg/d), brotizolam (≤0.125 mg/d); antidepressants with anxiolytic profile (SSRI^e^). *E, P* If used as hypnotics / sedatives: see alternatives proposed for drugs coded with N05C. |
| N05BA02 | Chlordiazepoxide  In lists: 1, 4, 5 (A); 5, 6 (B) | 19; 1.37 [1.08-1.66]; 1.00 | Risk of falling with hip fracture; prolonged reaction times; psychiatric reactions (can also be paradoxical, e.g. agitation, irritability, hallucinations, psychosis); cognitive impairment; depression | Use the lowest possible dose, up to half of the usual dose, taper in and out, shortest possible duration of treatment. *P* Reduce dose; for older adults use daily oral dose of 5 mg two to four times a day; in cases of severe renal failure (CrCl <10 ml/min), decrease dose by 50%. *M* | Non-pharmacological treatment; low doses of short-acting benzodiazepines such as lormetazepam (≤0.5 mg/d), brotizolam (≤0.125 mg/d); antidepressants with anxiolytic profile (SSRI^e^). *E, P* If used as hypnotics / sedatives: see alternatives proposed for drugs coded with N05C. |
| N05BA03 | Medazepam  In lists: 4 (A); 2, 5, 6 (B) | 14; 1.50 [1.12-1.88]; 1.00 | Risk of falling with hip fracture; prolonged reaction times; psychiatric reactions (can also be paradoxical, e.g. agitation, irritability, hallucinations, psychosis); cognitive impairment; depression | Use the lowest possible dose, up to half of the usual dose, taper in and out, shortest possible duration of treatment*. P* Reduce dose for older adults and for patients with renal failure. *M* | Non-pharmacological treatment; low doses of short-acting benzodiazepines such as lormetazepam (≤0.5 mg/d), brotizolam (≤0.125 mg/d); antidepressants with anxiolytic profile (SSRI^e^). *E, P* If used as hypnotics / sedatives: see alternatives proposed for drugs coded with N05C. |
| N05BA04 | Oxazepam (>60 mg/d)  In lists: 1, 4, 5 (A); 5, 6 (B) | 22; 1.50 [1.20-1.80]; 1.00 | Risk of falling with hip fracture; prolonged reaction times; psychiatric reactions (can also be paradoxical, e.g. agitation, irritability, hallucinations, psychosis); cognitive impairment; depression | Use the lowest possible dose, up to half of the usual dose, taper in and out, shortest possible duration of treatment. *P* Use doses of 10-20 mg/d; maximum dose: 30 mg/d. *E* | Non-pharmacological treatment; low doses of short-acting benzodiazepines such as lormetazepam (≤0.5 mg/d), brotizolam (≤0.125 mg/d); antidepressants with anxiolytic profile (SSRI^e^). *E, P* If used as hypnotics / sedatives: see alternatives proposed for drugs coded with N05C. |
| N05BA05 | Dipotassium clorazepate  In lists: 1, 4 (A); 2, 5, 6 (B) | 15; 1.40 [0.99-1.81]; 1.00 | Risk of falling with hip fracture; prolonged reaction times; psychiatric reactions (can also be paradoxical, e.g. agitation, irritability, hallucinations, psychosis); cognitive impairment; depression | Use the lowest possible dose, up to half of the usual dose, taper in and out, shortest possible duration of treatment. *P* | Non-pharmacological treatment; low doses of short-acting benzodiazepines such as lormetazepam (≤0.5 mg/d), brotizolam (≤0.125 mg/d); antidepressants with anxiolytic profile (SSRI^e^). *E, P* If used as hypnotics / sedatives: see alternatives proposed for drugs coded with N05C. |
| N05BA06 | Lorazepam (>1 mg/d)  In lists: 1, 4, 5 (A); 5, 6 (B) | 21; 1.67 [1.23-2.11]; 1.00 | Risk of falling with hip fracture; prolonged reaction times; psychiatric reactions (can also be paradoxical, e.g. agitation, irritability, hallucinations, psychosis); cognitive impairment; depression | Reduce dose; use doses of 0.25-1 mg/d. *E* | Non-pharmacological treatment; low doses of short-acting benzodiazepines such as lormetazepam (≤0.5 mg/d), brotizolam (≤0.125 mg/d); antidepressants with anxiolytic profile (SSRI^e^). *E, P* If used as hypnotics / sedatives: see alternatives proposed for drugs coded with N05C. |
| N05BA08 | Bromazepam  In lists: 1, 4 (A); 5, 6 (B) | 19; 1.63 [1.30-1.96]; 2.00 | Risk of falling with hip fracture; prolonged reaction times; psychiatric reactions (can also be paradoxical, e.g. agitation, irritability, hallucinations, psychosis); cognitive impairment; depression | Use the lowest possible dose, up to half of the usual dose, taper in and out, shortest possible duration of treatment. *P* | Non-pharmacological treatment; low doses of short-acting benzodiazepines such as lormetazepam (≤0.5 mg/d), brotizolam (≤0.125 mg/d); antidepressants with anxiolytic profile (SSRI^e^). *E, P* If used as hypnotics / sedatives: see alternatives proposed for drugs coded with N05C. |
| N05BA09 | Clobazam  In lists: 1, 3, 4 (A), 5, 6 (B) | 17; 1.41 [1.09-1.73]; 1.00 | Risk of falling with hip fracture; prolonged reaction times; psychiatric reactions (can also be paradoxical, e.g. agitation, irritability, hallucinations, psychosis); cognitive impairment; depression | Use the lowest possible dose, up to half of the usual dose, taper in and out, shortest possible duration of treatment. *E,* *P* Reduce dose; start with 5 mg/d orally and titrate no faster than every 7 days to 10-20 mg/d in 2 divided doses, depending on weight. If well tolerated, further titrate if necessary starting on day 21 to a maximum of 20-40 mg/d, depending on weight; older adults may receive half of the usual adult dose*. M* | Non-pharmacological treatment; low doses of short-acting benzodiazepines such as lormetazepam (≤0.5 mg/d), brotizolam (≤0.125 mg/d); antidepressants with anxiolytic profile (SSRI^e^). *E, P* If used as hypnotics / sedatives: see alternatives proposed for drugs coded with N05C. |
| N05BA11 | Prazepam  In lists: 1, 4 (A); 2, 5 (B) | 16; 1.31 [0.99-1.63]; 1.00 | Risk of falling with hip fracture; prolonged reaction times; psychiatric reactions (can also be paradoxical, e.g. agitation, irritability, hallucinations, psychosis); cognitive impairment; depression | Use the lowest possible dose, up to half of the usual dose, taper in and out, shortest possible duration of treatment. *P* Reduce dose; for older adults or debilitated patients, start with 10-15 mg/d orally (in divided doses). *M* | Non-pharmacological treatment; low doses of short-acting benzodiazepines such as lormetazepam (≤0.5 mg/d), brotizolam (≤0.125 mg/d); antidepressants with anxiolytic profile (SSRI^e^). *E, P* If used as hypnotics / sedatives: see alternatives proposed for drugs coded with N05C. |
| N05BA12 | Alprazolam  In lists: 1, 3, 4, 5 (A); 5, 6 (B) | 22; 1.91 [1.40-2.42]; 2.00 | Risk of falling with hip fracture; prolonged reaction times; psychiatric reactions (can also be paradoxical, e.g. agitation, irritability, hallucinations, psychosis); cognitive impairment; depression | Use the lowest possible dose, up to half of the usual dose, taper in and out, shortest possible duration of treatment. *P* Starting dose 0.25mg/12h. *E* Immediate release tablets (including orally disintegrating tablets): start with 0.25 mg administered two to three times a day, and titrate as tolerated; extended-release tablets: start with 0.5 mg once daily, gradually increase as needed and tolerated. *M* | Non-pharmacological treatment; low doses of short-acting benzodiazepines such as lormetazepam (≤0.5 mg/d), brotizolam (≤0.125 mg/d); antidepressants with anxiolytic profile (SSRI^e^). *E, P* If used as hypnotics / sedatives: see alternatives proposed for drugs coded with N05C. |
| N05BA13 | Halazepam  In lists: 6 (B) | 9; 2.00 [1.33-2.67]; 2.00 | Risk of falling with hip fracture; prolonged reaction times; psychiatric reactions (can also be paradoxical, e.g. agitation, irritability, hallucinations, psychosis); cognitive impairment; depression | Reduce dose; start with 20 mg once or twice daily for patients 70 years or older; adjust dose according to response. *M, E* | Non-pharmacological treatment; low doses of short-acting benzodiazepines such as lormetazepam (≤0.5 mg/d), brotizolam (≤0.125 mg/d); antidepressants with anxiolytic profile (SSRI^e^). *E, P* If used as hypnotics / sedatives: see alternatives proposed for drugs coded with N05C. |
| N05BA16 | Nordazepam  In lists: 1 (A); 2, 5, 6 (B) | 12; 1.75 [1.20-2.30]; 1.50 | Risk of falling with hip fracture; prolonged reaction times; psychiatric reactions (can also be paradoxical, e.g. agitation, irritability, hallucinations, psychosis); cognitive impairment; depression | Reduce dose. *M* | Non-pharmacological treatment; low doses of short-acting benzodiazepines such as lormetazepam (≤0.5 mg/d), brotizolam (≤0.125 mg/d); antidepressants with anxiolytic profile (SSRI^e^). *E, P* If used as hypnotics / sedatives: see alternatives proposed for drugs coded with N05C. |
| N05BA18 | (Ethyl-) Loflazepate  In lists: 1 (A); 5, 6 (B) | 12; 1.75 [1.20-2.30]; 1.50 | Risk of falling with hip fracture; prolonged reaction times; psychiatric reactions (can also be paradoxical, e.g. agitation, irritability, hallucinations, psychosis); cognitive impairment; depression | Reduce dose. *M* | Non-pharmacological treatment; low doses of short-acting benzodiazepines such as lormetazepam (≤0.5 mg/d), brotizolam (≤0.125 mg/d); antidepressants with anxiolytic profile (SSRI^e^). *E, P* If used as hypnotics / sedatives: see alternatives proposed for drugs coded with N05C. |
| N05BA21 | Clotiazepam (>5 mg/d)  In lists: 1 (A); 5, 6 (B) | 16; 1.56 [1.17-1.95]; 1.00 | Risk of falling with hip fracture; prolonged reaction times; psychiatric reactions (can also be paradoxical, e.g. agitation, irritability, hallucinations, psychosis); cognitive impairment; depression | Reduce dose. *M* | Non-pharmacological treatment; low doses of short-acting benzodiazepines such as lormetazepam (≤0.5 mg/d), brotizolam (≤0.125 mg/d); antidepressants with anxiolytic profile (SSRI^e^). *E, P* If used as hypnotics / sedatives: see alternatives proposed for drugs coded with N05C. |
| N05BC01 | Meprobamate  In lists: 1, 5 (A) | 18; 1.33 [1.09-1.57]; 1.00 | Risk of drowsiness, confusion | Reduce dose; start low–go slow; increase dosage interval in cases of renal failure; administer every 6 hours in cases of mild renal failure (GFR>50 ml/min), every 9 to 12 hours in cases of moderate renal failure (10 to 50 ml/min) and every 12 to 18 hours in cases of severe renal failure. *M* | Non-pharmacological treatment; low doses of short-acting benzodiazepines such as lormetazepam (≤0.5 mg/d), brotizolam (≤0.125 mg/d); antidepressants with anxiolytic profile (SSRI^e^). *E, P* If used as hypnotics / sedatives: see alternatives proposed for drugs coded with N05C. |
| ***N05C*** | ***Hypnotics and sedatives*** |  |  |  |  |
| N05CC01 | Chloralhydrate  In lists: 4, 5 (A); 5 (B) | 17; 1.53 [1.21-1.85]; 1.00 | Risk of dizziness and electrocardiographic changes. Higher risk in cases of renal failure | Use the lowest possible dose, up to half of the usual dose, taper in and out, shortest possible duration of treatment. *P* For the management of insomnia in geriatric patients, use initial oral dose of 250 mg/d. *M* | Non-pharmacological treatment; mirtazapine^d^; passiflora, low doses of short-acting benzodiazepines such as lormetazepam (≤0.5 mg/d), brotizolam (≤0.125 mg/d); zolpidem (≤5 mg/d), zopiclon (≤3.75 mg/d), zaleplon (≤5 mg/d); trazodone. *E, P* |
| N05CD01 | Flurazepam  In lists: 4, 5 (A); 5, 6 (B) | 20; 1.25 [1.04-1.46]; 1.00 | Risk of falls and hip fracture, prolonged reaction time, psychiatric reactions (which can be paradoxical, e.g. agitation, irritability, hallucinations, psychosis), cognitive impairment and depression | Use the lowest possible dose, up to half of the usual dose, taper in and out, shortest possible duration of treatment. *P* Start with 15 mg/d. *M* | Non-pharmacological treatment; mirtazapine^d^; passiflora, low doses of short-acting benzodiazepines such as lormetazepam (≤0.5 mg/d), brotizolam (≤0.125 mg/d); zolpidem (≤5 mg/d), zopiclon (≤3.75 mg/d), zaleplon (≤5 mg/d); trazodone. *E, P* |
| N05CD02 | Nitrazepam  In lists: 1, 3, 4 (A); 2, 5, 6 (B) | 20; 1.40 [1.12-1.68]; 1.00 | Risk of falls and hip fracture, prolonged reaction time, psychiatric reactions (which can be paradoxical, e.g. agitation, irritability, hallucinations, psychosis), cognitive impairment and depression | Use the lowest possible dose, up to half of the usual dose, taper in and out, shortest possible duration of treatment. *P* Use 2.5-5 mg/d at bedtime. *E, M* | Non-pharmacological treatment; mirtazapine^d^; passiflora, low doses of short-acting benzodiazepines such as lormetazepam (≤0.5 mg/d), brotizolam (≤0.125 mg/d); zolpidem (≤5 mg/d), zopiclon (≤3.75 mg/d), zaleplon (≤5 mg/d); trazodone. *E, P* |
| N05CD03 | Flunitrazepam  In lists: 1, 4 (A); 5, 6 (B) | 22; 1.32 [1.03-1.60]; 1.00 | Risk of falls and hip fracture, prolonged reaction time, psychiatric reactions (which can be paradoxical, e.g. agitation, irritability, hallucinations, psychosis), cognitive impairment and depression | Use the lowest possible dose, up to half of the usual dose, taper in and out, shortest possible duration of treatment. P Reduce dose, e.g. 0.5 mg/d; start low–go slow. *E, M* For induction of anaesthesia in older, poor-risk adults, titrate dose carefully; administer in small intravenous increments of 0.3 to 0.5 mg, at 30-second intervals. *M* | Non-pharmacological treatment; mirtazapine^d^; passiflora, low doses of short-acting benzodiazepines such as lormetazepam (≤0.5 mg/d), brotizolam (≤0.125 mg/d); zolpidem (≤5 mg/d), zopiclon (≤3.75 mg/d), zaleplon (≤5 mg/d); trazodone. *E, P* |
| N05CD04 | Estazolam  In lists: 1, 5 (A); 5, 6 (B) | 12; 1.42 [0.99-1.84]; 1.00 | Risk of falls and hip fracture, prolonged reaction time, psychiatric reactions (which can be paradoxical, e.g. agitation, irritability, hallucinations, psychosis), cognitive impairment and depression | For older adults who are debilitated or have a low weight, consider initial dose of 0.5 mg at bedtime. *M* | Non-pharmacological treatment; mirtazapine^d^; passiflora, low doses of short-acting benzodiazepines such as lormetazepam (≤0.5 mg/d), brotizolam (≤0.125 mg/d); zolpidem (≤5 mg/d), zopiclon (≤3.75 mg/d), zaleplon (≤5 mg/d); trazodone. *E, P* |
| N05CD05 | Triazolam  In lists: 1, 2, 3, 4, 5 (A); 5, 6 (B) | 18; 1.67 [1.18-2.15]; 1.00 | Risk of falls and hip fracture, prolonged reaction time, psychiatric reactions (which can be paradoxical, e.g. agitation, irritability, hallucinations, psychosis), cognitive impairment and depression | Use the lowest possible dose, up to half of the usual dose, taper in and out, shortest possible duration of treatment. *P* Reduce dose: 0.125-0.25 mg/d at bedtime Start low–go slow. *E, M* | Non-pharmacological treatment; mirtazapine^d^; passiflora, low doses of short-acting benzodiazepines such as lormetazepam (≤0.5 mg/d), brotizolam (≤0.125 mg/d); zolpidem (≤5 mg/d), zopiclon (≤3.75 mg/d), zaleplon (≤5 mg/d); trazodone. *E, P* |
| N05CD06 | Lormetazepam (>0.5 mg/d)  In lists: 1, 4 (A); 5, 6 (B) | 17; 1.47 [1.15-1.79]; 1.00 | Risk of falls and hip fracture, prolonged reaction time, psychiatric reactions (which can be paradoxical, e.g. agitation, irritability, hallucinations, psychosis), cognitive impairment and depression | Use the lowest possible dose, up to half of the usual dose, taper in and out, shortest possible duration of treatment. *P* | Non-pharmacological treatment; mirtazapine^d^; passiflora, low doses of short-acting benzodiazepines such as lormetazepam (≤0.5 mg/d), brotizolam (≤0.125 mg/d); zolpidem (≤5 mg/d), zopiclon (≤3.75 mg/d), zaleplon (≤5 mg/d); trazodone. *E, P* |
| N05CD07 | Temazepam  In lists: 1, 4, 5 (A); 5, 6 (B) | 17; 1.88 [1.34-2.42]; 2.00 | Risk of falls and hip fracture, prolonged reaction time, psychiatric reactions (which can be paradoxical, e.g. agitation, irritability, hallucinations, psychosis), cognitive impairment and depression | Use the lowest possible dose, up to half of the usual dose, taper in and out, shortest possible duration of treatment. *P* Start with 7.5 mg/d and watch individual response. *M* | Non-pharmacological treatment; mirtazapine^d^; passiflora, low doses of short-acting benzodiazepines such as lormetazepam (≤0.5 mg/d), brotizolam (≤0.125 mg/d); zolpidem (≤5 mg/d), zopiclon (≤3.75 mg/d), zaleplon (≤5 mg/d); trazodone. *E, P* |
| N05CD08 | Midazolam  In lists: 3 (A); 5, 6 (B) | 22; 2.45 [1.93-2.98]; 2.50 | Risk of falls and hip fracture, prolonged reaction time, psychiatric reactions (which can be paradoxical, e.g. agitation, irritability, hallucinations, psychosis), cognitive impairment and depression | Reduce dose to 50% of the dose used in healthy younger adults; start with 0.5-1 mg/d. *E* In cases of severe renal failure (CrCl <10 ml/min), the dose should be decreased by 50%. *M* | Non-pharmacological treatment; mirtazapine^d^; passiflora, low doses of short-acting benzodiazepines such as lormetazepam (≤0.5 mg/d), brotizolam (≤0.125 mg/d); zolpidem (≤5 mg/d), zopiclon (≤3.75 mg/d), zaleplon (≤5 mg/d); trazodone. *E, P* |
| N05CD09 | Brotizolam (>0.125 mg/d)  In lists: 4 (A); 5, 6 (B) | 15; 1.73 [1.29-2.18]; 2.00 | Risk of falls and hip fracture, prolonged reaction time, psychiatric reactions (which can be paradoxical, e.g. agitation, irritability, hallucinations, psychosis), cognitive impairment and depression | Reduce dose; start low–go slow. *E* Use the lowest possible dose, up to half of the usual dose, taper in and out, shortest possible duration of treatment. *P* | Non-pharmacological treatment; mirtazapine^d^; passiflora, low doses of short-acting benzodiazepines such as lormetazepam (≤0.5 mg/d), brotizolam (≤0.125 mg/d); zolpidem (≤5 mg/d), zopiclon (≤3.75 mg/d), zaleplon (≤5 mg/d); trazodone. *E, P* |
| N05CD10 | Quazepam  In lists: 5 (A); 2, 5, 6 (B) | 11; 1.82 [1.31-2.32]; 2.00 | Risk of falls and hip fracture, prolonged reaction time, psychiatric reactions (which can be paradoxical, e.g. agitation, irritability, hallucinations, psychosis), cognitive impairment and depression | Reduce dose; start low–go slow. *E* | Non-pharmacological treatment; mirtazapine^d^; passiflora, low doses of short-acting benzodiazepines such as lormetazepam (≤0.5 mg/d), brotizolam (≤0.125 mg/d); zolpidem (≤5 mg/d), zopiclon (≤3.75 mg/d), zaleplon (≤5 mg/d); trazodone. *E, P* |
| N05CD11 | Loprazolam (>0.5 mg/d)^c^  In lists: 1 (A); 5, 6 (B) | 16; 1.63 [1.24-2.01]; 1.50 | Risk of falls and hip fracture, prolonged reaction time, psychiatric reactions (which can be paradoxical, e.g. agitation, irritability, hallucinations, psychosis), cognitive impairment and depression | Reduce dose; start low–go slow. Use the lowest possible dose, up to half of the usual dose, taper in and out, shortest possible duration of treatment. *P*; *E* | Non-pharmacological treatment; mirtazapine^d^; passiflora, low doses of short-acting benzodiazepines such as lormetazepam (≤0.5 mg/d), brotizolam (≤0.125 mg/d); zolpidem (≤5 mg/d), zopiclon (≤3.75 mg/d), zaleplon (≤5 mg/d); trazodone. *E, P* |
| N05CF01 | Zopiclone (>3.75 mg/d)  In lists: 1, 4, 5, 6 (A); 5 (B) | 22; 2.27 [1.82-2.73]; 2.00 | Risk of falls and hip fracture, prolonged reaction time, psychiatric reactions (which can be paradoxical, e.g. agitation, irritability, hallucinations, psychosis), cognitive impairment and depression | Use the lowest possible dose, up to half of the usual dose, taper in and out, shortest possible duration of treatment. *P* | Non-pharmacological treatment; mirtazapine^d^; passiflora, low doses of short-acting benzodiazepines such as lormetazepam (≤0.5 mg/d), brotizolam (≤0.125 mg/d); zolpidem (≤5 mg/d), zopiclon (≤3.75 mg/d), zaleplon (≤5 mg/d); trazodone. *E, P* |
| N05CF02 | Zolpidem (>5 mg/d)  In lists: 1, 4, 5, 6 (A); 5 (B) | 22; 2.09 [1.66-2.52]; 2.00 | Risk of falls and hip fracture, prolonged reaction time, psychiatric reactions (which can be paradoxical, e.g. agitation, irritability, hallucinations, psychosis), cognitive impairment and depression | Use the lowest possible dose, up to half of the usual dose, taper in and out, shortest possible duration of treatment. *P* | Non-pharmacological treatment; mirtazapine^d^; passiflora, low doses of short-acting benzodiazepines such as lormetazepam (≤0.5 mg/d), brotizolam (≤0.125 mg/d); zolpidem (≤5 mg/d), zopiclon (≤3.75 mg/d), zaleplon (≤5 mg/d); trazodone. *E, P* |
| N05CF03 | Zaleplone (>5 mg/d)  In lists: 3, 4, 5, 6 (A); 5 (B) | 17; 1.94 [1.56-2.33]; 2.00 | Risk of falls and hip fracture, prolonged reaction time, psychiatric reactions (which can be paradoxical, e.g. agitation, irritability, hallucinations, psychosis), cognitive impairment and depression | Use the lowest possible dose, up to half of the usual dose, taper in and out, shortest possible duration of treatment. *P* | Non-pharmacological treatment; mirtazapine^d^; passiflora, low doses of short-acting benzodiazepines such as lormetazepam (≤0.5 mg/d), brotizolam (≤0.125 mg/d); zolpidem (≤5 mg/d), zopiclon (≤3.75 mg/d), zaleplon (≤5 mg/d); trazodone. *E, P* |
| N05CM02 | Clomethiazole  In lists: 5 (B) | 13; 2.23 [1.53-2.94]; 2.00 | Risk of respiratory depression | Reduce dose. *E, M* Use sedative dose 500-1000 mg at bedtime. *M* | Non-pharmacological treatment; mirtazapine^d^; passiflora, low doses of short-acting benzodiazepines such as lormetazepam (≤0.5 mg/d), brotizolam (≤0.125 mg/d); zolpidem (≤5 mg/d), zopiclon (≤3.75 mg/d), zaleplon (≤5 mg/d); trazodone. *E, P* |
| N05CM06 | Propiomazine  In lists: 5, 6 (B) | 10; 1.20 [0.90-1.50]; 1.00 | Risk of antimuscarinic effects, sedation and hypotension, dry mouth and extrapyramidal reactions |  | Non-pharmacological treatment; mirtazapine^d^; passiflora, low doses of short-acting benzodiazepines such as lormetazepam (≤0.5 mg/d), brotizolam (≤0.125 mg/d); zolpidem (≤5 mg/d), zopiclon (≤3.75 mg/d), zaleplon (≤5 mg/d); trazodone. *E, P* |
| No ATC | Aceprometazine  In lists: 1 (A); 6 (B) | 14; 1.64 [1.21-2.07]; 1.50 | Muscarinic-blocking drug, risk of cognitve impairment |  | Non-pharmacological treatment; mirtazapine^d^; passiflora, low doses of short-acting benzodiazepines such as lormetazepam (≤0.5 mg/d), brotizolam (≤0.125 mg/d); zolpidem (≤5 mg/d), zopiclon (≤3.75 mg/d), zaleplon (≤5 mg/d); trazodone. *E, P* |
| ***N06*** | ***Psychoanaleptics*** |  |  |  |  |
| ***N06A*** | ***Antidepressants*** |  |  |  |  |
| N06AA01 | Desipramine  In lists: 2, 5, 6 (B) | 14; 1.50 [1.12-1.88]; 1.00 | Peripheral anticholinergic side effects (e.g. constipation, dry mouth, orthostatic hypotension, cardiac arrhythmia); central anticholinergic side effects (drowsiness, inner unrest, confusion, other types of delirium); cognitive deficit; increased risk of falling | Use doses of 25-100 mg/d; maximum dose: 150 mg/d. *M* | Non-pharmacological treatment, SSRI (except PIM: fluoxetine, paroxetine, fluvoxamine)^e^, mirtazapine^d^, trazodone. *E* |
| N06AA02 | Imipramine  In lists: 1, 4, 5 (A); 2, 5, 6 (B) | 20; 1.50 [1.14-1.86]; 1.00 | Peripheral anticholinergic side effects (e.g. constipation, dry mouth, orthostatic hypotension, cardiac arrhythmia); central anticholinergic side effects (drowsiness, inner unrest, confusion, other types of delirium); cognitive deficit; increased risk of falling | Start at half the usual daily dose, increase slowly; reduce dose. *P* Use doses of 25-50 mg/d at bedtime; maximum dose: 100 mg/d. *E* | Non-pharmacological treatment, SSRI (except PIM: fluoxetine, paroxetine, fluvoxamine)^e^, mirtazapine^d^, trazodone. *E* |
| N06AA04 | Clomipramine  In lists: 1, 3, 4, 5 (A); 1, 2, 5, 6 (B) | 21; 1.48 [1.14-1.82]; 1.00 | Peripheral anticholinergic side effects (e.g. constipation, dry mouth, orthostatic hypotension, cardiac arrhythmia); central anticholinergic side effects (drowsiness, inner unrest, confusion, other types of delirium); cognitive deficit; increased risk of falling | Start with half the usual daily dose, increase slowly; reduce dose. *E, M, P* Starting dose 10-20 mg/d, max. 250 mg/day. *E* | Non-pharmacological treatment, SSRI (except PIM: fluoxetine, paroxetine, fluvoxamine)^e^, mirtazapine^d^, trazodone. *E* |
| N06AA06 | Trimipramine  In lists: 1, 3, 4, 5 (A); 2, 5, 6 (B) | 16; 1.44 [1.10-1.77]; 1.00 | Peripheral anticholinergic side effects (e.g. constipation, dry mouth, orthostatic hypotension, cardiac arrhythmia); central anticholinergic side effects (drowsiness, inner unrest, confusion, other types of delirium); cognitive deficit; increased risk of falling | Start at half the usual daily dose, increase slowly; reduce dose. *M, P* Start with 50 mg/d and do not exceed 100 mg/d. *M* | Non-pharmacological treatment, SSRI (except PIM: fluoxetine, paroxetine, fluvoxamine)^e^, mirtazapine^d^, trazodone. *E* |
| N06AA09 | Amitriptyline  In lists: 1, 3, 4, 5 (A); 2, 5, 6 (B) | 22; 1.68 [1.26-2.10]; 1.00 | Peripheral anticholinergic side effects (e.g. constipation, dry mouth, orthostatic hypotension, cardiac arrhythmia); central anticholinergic side effects (drowsiness, inner unrest, confusion, other types of delirium); cognitive deficit; increased risk of falling | Start at half the usual daily dose, increase slowly; reduce dose; start with 10 mg 3 times per day and 20 mg at bedtime. *M, E, P* Its use for treating neuropathic pain may be considered appropriate, with benefits overweighing the risks. *E* | Non-pharmacological treatment, SSRI (except PIM: fluoxetine, paroxetine, fluvoxamine)^e^, mirtazapine^d^, trazodone. *E* |
| N06AA10 | Nortriptyline  In lists: 3 (A); 2, 5, 6 (B) | 21; 2.10 [2.52-2.67]; 2.00 | Peripheral anticholinergic side effects (e.g. constipation, dry mouth, orthostatic hypotension, cardiac arrhythmia); central anticholinergic side effects (drowsiness, inner unrest, confusion, other types of delirium); cognitive deficit; increased risk of falling | Use 30-50 mg/d in divided doses*. E, M* Its use for treating neuropathic pain may be considered appropriate, with benefits overweighing the risks. *E* | Non-pharmacological treatment, SSRI (except PIM: fluoxetine, paroxetine, fluvoxamine)^e^, mirtazapine^d^, trazodone. *E* |
| N06AA12 | Doxepin  In lists: 1, 3, 4, 5 (A); 2, 5, 6 (B) | 20; 1.40 [1.05-1.75]; 1.00 | Peripheral anticholinergic side effects (e.g. constipation, dry mouth, orthostatic hypotension, cardiac arrhythmia); central anticholinergic side effects (drowsiness, inner unrest, confusion, other types of delirium); cognitive deficit; increased risk of falling | Start at half the usual daily dose, increase slowly. *P* 0.5 mg/d. *E* 3 mg/d, maximum dose: 6 mg/d. *M* | Non-pharmacological treatment, SSRI (except PIM: fluoxetine, paroxetine, fluvoxamine)^e^, mirtazapine^d^, trazodone. *E* |
| N06AA16 | Dosulepin  In lists: 1 (A); 2, 5, 6 (B) | 17; 1.29 [1.05-1.54]; 1.00 | Muscarinic-blocking agents with cardiotoxicity when overdosed | Start with 50-75 mg/d. *E, M* Reduce dose in cases of renal failure. *M* | Non-pharmacological treatment, SSRI (except PIM: fluoxetine, paroxetine, fluvoxamine)^e^, mirtazapine^d^, trazodone. *E* |
| N06AA17 | Amoxapine  In lists: 1 (A); 2, 5, 6 (B) | 14; 1.50 [1.12-1.88]; 1.00 | Muscarinic-blocking agents with cardiotoxicity when overdosed | Start with 25 mg given two to three times per day; by the end of the first week, increase to 50 mg given two to three times per day. 2-3x/d. *M* | Non-pharmacological treatment, SSRI (except PIM: fluoxetine, paroxetine, fluvoxamine)^e^, mirtazapine^d^, trazodone. *E* |
| N06AA21 | Maprotiline  In lists: 1, 4 (A); 2, 5, 6 (B) | 21; 1.43 [1.09-1.77]; 1.00 | Peripheral anticholinergic side effects (e.g. constipation, dry mouth, orthostatic hypotension, cardiac arrhythmia); central anticholinergic side effects (drowsiness, inner unrest, confusion, other types of delirium); cognitive deficit; increased risk of falling | Start at half the usual daily dose, increase slowly; reduce dose. *P, E* Start with 25 mg/d, increase by 25 mg increments up to 50-75 mg/d. *M* | Non-pharmacological treatment, SSRI (except PIM: fluoxetine, paroxetine, fluvoxamine)^e^, mirtazapine^d^, trazodone. *E* |
| N06AB03 | Fluoxetine  In lists: 3, 4 (A); 2, 5, 6 (B) | 22; 2.27 [1.80-2.75]; 2.00 | CNS side effects (nausea, insomnia, dizziness, confusion); hyponatremia | Reduce dose; start with 20 mg/d; maximum dose also 20 mg/d; avoid administration at bedtime. *E, M* | Non-pharmacological treatment, SSRI (except PIM: fluoxetine, paroxetine, fluvoxamine)^e^, mirtazapine^d^, trazodone. *E* |
| N06AB05 | Paroxetine  In lists: 2, 5, 6 (B) | 21; 2.29 [1.99-2.58]; 2.00 | Higher risk of all-cause mortality, higher risk of seizures, falls and fractures. Anticholinergic adverse effects | For older adults or for patients with renal failure, start immediate-release tablets with 10 mg/d (12.5 mg/d if controlled-release tablets), increased by 10 mg/d (12.5 mg/d if controlled-release tablets), up to 40 mg/d (50 mg/d if controlled-release tablets)*. E, M* | Non-pharmacological treatment, SSRI (except PIM: fluoxetine, paroxetine, fluvoxamine)^e^, mirtazapine^d^, trazodone. *E* |
| N06AB08 | Fluvoxamine  In lists: 2, 5, 6 (B) | 20; 2.05 [1.69-2.41]; 2.00 | Higher risk of all-cause mortality, self-harm, falls, fractures and hyponatraemia | Reduce dose for older adults and patients with renal failure; start with 50-100 mg/d; titrate slowly. *E, M* | Non-pharmacological treatment, SSRI (except PIM: fluoxetine, paroxetine, fluvoxamine)^e^, mirtazapine^d^, trazodone. *E* |
| N06AF04 | Tranylcypromine  In lists: 4 (A) | 15; 1.73 [1.06-2.41]; 1.00 | Irreversible MAO inhibitor. Risk of hypertensive crises, cerebral hemorrhage and malignant hyperthermia | Reduce dose: 30 mg/d; maximum dose: 60 mg/d. *E* | Non-pharmacological treatment, SSRI (except PIM: fluoxetine, paroxetine, fluvoxamine)^e^, mirtazapine^d^, trazodone. *E* |
| N06AX12 | Bupropion  In lists: 5 (B) | 20; 2.30 [1.77-2.83]; 2.00 | May lower seizure threshold | Reduce dose and dosing frequency for older adults and patients with renal failure. *M* | Non-pharmacological treatment, SSRI (except PIM: fluoxetine, paroxetine, fluvoxamine)^e^, mirtazapine^d^, trazodone. *E* |
| N06AX16 | Venlafaxine  In lists: does not appear as PIM | 21; 2.43 [2.06-2.80]; 2.00 | Higher risk of all-cause mortality, attempted suicide, stroke, seizures, upper gastrointestinal bleeding, falls and fracture | Start with 25-50 mg, two times per day and increase by 25 mg/dose; for extended-release formulation start with 37.5 mg once daily and increase by 37.5 mg every 4-7 days as tolerated. *E* Reduce the total daily dose by 25-50% in cases of mild to moderate renal failure. *M* | Non-pharmacological treatment, SSRI (except PIM: fluoxetine, paroxetine, fluvoxamine)^e^, mirtazapine^d^, trazodone. *E* |
| N06AX18 | Reboxetine  In lists: does not appear as PIM | 15; 1.87 [1.46-2.28]; 2.00 | Side effects (dry mouth, constipation, headache, drowsiness, dizziness, excessive sweating and insomnia). Higher risk of conduction disturbances, tachycardia, occasional atrial and ventricular ectopy | Reduce dose in cases of renal failure; start with 2 mg two times per day in cases of renal failure; for older adults, reduce dose to 4-6 mg/d. *M* | Non-pharmacological treatment, SSRI (except PIM: fluoxetine, paroxetine, fluvoxamine)^e^, mirtazapine^d^, trazodone. *E* |
| ***N06B*** | ***Psychostimulants, agents used for ADHD and nootropics*** |  |  |  |  |
| N06BA04 | Methylphenidat  In lists: 2 (A); 5 (B) | 19; 1.63 [1.14-2.12]; 1.00 | May cause or worsen insomnia;  concern due to CNS-altering effects;  concern due to appetite-supressing effects |  | Non-pharmacological treatment; consider pharmacotherapy of Alzheimer-type dementia: acetylcholinesterase, memantine^d^. *E* |
| N06BX03 | Piracetam  In lists: 1, 4 (A) | 19; 2.05 [1.40-2.70]; 2.00 | No efficacy proven; unfavorable risk/benefit profile | Reduce dose for older adults and for patients with renal failure. *M* | Non-pharmacological treatment; consider pharmacotherapy of Alzheimer-type dementia: acetylcholinesterase, memantine^d^ *E* |
| ***N06D*** | ***Anti-dementia drugs*** |  |  |  |  |
| N06DX02 | Ginkgo biloba  In lists: 1 (A) | 20; 2.05 [1.42-2.68]; 1.50 | No efficacy proven; increased risk of orthostatic hypotension and fall |  | Non-pharmacological treatment; consider pharmacotherapy of Alzheimer-type dementia: acetylcholinesterase, memantine^d^. *E* |
| C04AE01 | Ergoloid mesylate (dihydroergotoxine)  In lists: 1, 4 (A); 6 (B) | 21; 1.48 [1.03-1.92]; 1.00 | No efficacy proven; unfavourable risk/benefit profile; increased risk of orthostatic hypotension and fall | 1 mg three times daily. *M* | Non-pharmacological treatment; consider pharmacotherapy of Alzheimer-type dementia: acetylcholinesterase, memantine^d^. *E* |
| ***N07*** | ***Other nervous system drugs*** |  |  |  |  |
| ***N07A*** | ***Parasympathomimetics*** |  |  |  |  |
| N07AB02 | Bethanechol  In lists: does not appear as PIM | 14; 1.71 [1.24-2.19]; 1.50 | Anticholinergic bladder relaxants may cause obstruction in persons with benign prostatic hyperplasia |  |  |
| **R** | **Respiratory system** |  |  |  |  |
| ***R01*** | ***Nasal preparations*** |  |  |  |  |
| ***R01B*** | ***Nasal decongestants for systemic use*** |  |  |  |  |
| R01BA01 | Norephedrine (=Phenylpropanolamine)  In lists: 3 (A) | 21; 2.05 [1.56-2.54]; 2.00 | Higher risk of elevation of blood pressure secondary to sympathomimetic activity |  |  |
| R01BA02 | Pseudoephedrine  In lists: 5 (B) | 21; 2.00 [1.52-2.48]; 2.00 | Higher risk of elevation of blood pressure secondary to sympathomimetic activity | Adjust dose in cases of renal failure; 15-30 mg three times per day for the treatment of urinary incontinence in older adults. *M* |  |
| ***R03*** | ***Drugs for obstructive airway diseases*** |  |  |  |  |
| ***R03C*** | ***Adrenergics for systemic use*** |  |  |  |  |
| R03CC03 | Terbutaline (oral)  In lists: does not appear as PIM | 20; 1.75 [1.25-2.25]; 1.00 | Higher risk of adverse effects as compared to the inhaled form | Use 50% of the usual dose for patients with moderate renal failure (GFR 10-50 ml/min); avoid in cases of severe renal failure (GFR <10 ml/min). *M* | Inhaled form. *E* |
| ***R03D*** | ***Other systemic drugs for airway diseases*** |  |  |  |  |
| R03DA04 | Theophylline  In lists: 3 (A); 5, 6 (B) | 22; 2.27 [1.76-2.79]; 2.00 | Higher risk of CNS stimulant effects | Start with a 25% reduction compared to the doses for younger adults. *E* Start with a maximum dose of 400 mg/d; monitor serum levels and reduce doses if needed; for healthy older adults (>60 years), theophylline clearance is decreased by an average of 30%. *M* |  |
| ***R05*** | ***Cough and cold preparation*** |  |  |  |  |
| ***R05D*** | ***Cough suppressants, excl. combinations with expectorants*** |  |  |  |  |
| R05DA01 | Ethylmorphine  In lists: 3 (A) | 21; 1.90 [1.43-2.38]; 2.00 | No clear evidence in the treatment of acute cough |  |  |
| R05DA04 | Codeine (>2 weeks)  In lists: 6 (B) | 21; 2.00 [1.68-2.32]; 2.00 | Higher risk of adverse events (hypotension, sweating, constipation, vomiting, dizziness, sedation, respiratory depression). Avoid use for longer than 2 weeks for persons with chronic constipation without concurrent use of laxatives and for persons with renal failure | Start treatment cautiously  for older adults (especially in cases of renal failure); start low–go slow; reduce dose to 75% of the usual dose if GFR 10-50 ml/min and to 50% if GFR <10 ml/min. *M* | If used for pain management consider alternative drugs proposed for analgesics:  paracetamol; ibuprofen (≤3 x 400 mg/d or for a period shorter than one week); naproxen (≤2 x 250 mg/d or for a period shorter than one week). *E* Opioids with lower risk of delirium (e.g., tilidine/naloxone, morphine^d^, oxycodone, buprenorphine, hydromorphone). *E, P* |
| R05DA09 | Dextrometorphan  In lists: 3 (A) | 20; 2.10 [1.55-2.65]; 2.00 | No clear evidence in the treatment of acute cough |  |  |
| ***R06*** | ***Antihistamines for systemic use*** |  |  |  |  |
| ***R06A*** | ***Antihistamines for systemic use*** |  |  |  |  |
| R06AA02 | Diphenhydramine  In lists: 1, 4, 5 (A); 5, 6 (B) | 21; 1.48 [1.20-1.75]; 1.00 | Anticholinergic side effects, sedation, dizziness; electrocardiographic changes | Reduce dose for older adults; start low–go slow. *M* Use the lowest possible dose, up to half of the usual dose, taper in and out, shortest possible duration of treatment. *P* Increase the dosing interval to every 6 hours in cases of mild renal failure (GFR >50 ml/min), every 6-12 hours in cases of moderate renal failure (GFR 10-50 ml/min), and every 12-18 hours in cases of severe renal failure (GFR <10 ml/min). *M* | Non-sedating, non-anticholinergic antihistamines^f^ like loratadine, cetirizine, but not terfenadine (which is PIM). *E* If used for insomnia: non-pharmacological treatment, passiflora, mirtazapine^d^, trazodone. *E* Consider low doses of short-acting benzodiazepines such as lormetazepam (≤0.5 mg/d), brotizolam (≤0.125 mg/d); zolpidem (≤5 mg/d), zopiclon (≤3.75 mg/d), zaleplon (≤5 mg/d) (suggested aternatives to hypnotic/sedative drugs) |
| R06AA04 | Clemastine  In lists: 4 (A); 5, 6 (B) | 22; 1.77 [1.37-2.18]; 2.00 | Anticholinergic side effects (e.g. constipation, dry mouth); impaired cognitive performance;  electrocardiographic changes (prolonged QT) | Reduce dose. *M* | Non-sedating, non-anticholinergic antihistamines^f^ like loratadine, cetirizine, but not terfenadine (which is PIM). *E* |
| R06AA08 | Carbinoxamine  In lists: 1 (A); 5, 6 (B) | 14; 1.64 [1.16-2.13]; 1.00 | Muscarinic-blocking drug; higher risk of sedation, drowsiness | Start low–go slow. *M* | Non-sedating, non-anticholinergic antihistamines^f^ like loratadine, cetirizine, but not terfenadine (which is PIM). *E* |
| R06AA09 | Doxylamine  In lists: 1, 4, 5 (A); 5, 6 (B) | 16; 1.38 [1.05-1.70]; 1.00 | Anticholinergic side effects, dizziness; electrocardiographic changes | Reduce dose. *M* Use the lowest possible dose, up to half of the usual dose, taper in and out, shortest possible duration of treatment. *P* | Non-sedating, non-anticholinergic antihistamines^f^ like loratadine, cetirizine, but not terfenadine (which is PIM). *E* If used for insomnia: non-pharmacological treatment, passiflora, mirtazapine^d^, trazodone. *E* Consider low doses of short-acting benzodiazepines such as lormetazepam (≤0.5 mg/d), brotizolam (≤0.125 mg/d); zolpidem (≤5 mg/d), zopiclon (≤3.75 mg/d), zaleplon (≤5 mg/d) (suggested aternatives to hypnotic/sedative drugs) |
| R06AB01 | Brompheniramine  In lists: 1 (A); 5, 6 (B) | 15; 1.60 [1.14-2.06]; 1.00 | Muscarinic-blocking drug; higher risk of sedation, drowsiness |  | Non-sedating, non-anticholinergic antihistamines^f^ like loratadine, cetirizine, but not terfenadine (which is PIM). *E* |
| R06AB02 | Dexchlorpheniramine  In lists: 1, 4, 5 (A); 5, 6 (B) | 17; 1.47 [1.10-1.84]; 1.00 | Anticholinergic side effects (e.g. confusion, sedation) | 5 mg/d. *E* | Non-sedating, non-anticholinergic antihistamines^f^ like loratadine, cetirizine, but not terfenadine (which is PIM). *E* |
| R06AB03 | Dimetindene  In lists: 4 (A); 6 (B) | 16; 1.56 [1.13-2.00]; 1.00 | Anticholinergic side effects (e.g. constipation, dry mouth); impaired cognitive performance;  electrocardiographic changes (prolonged QT) |  | Non-sedating, non-anticholinergic antihistamines^f^ like loratadine, cetirizine, but not terfenadine (which is PIM). *E* |
| R06AB04 | Chlorpheniramine (=Chlorphenamine)  In lists: 1, 4 (A); 5, 6 (B) | 17; 1.41 [1.05-1.78]; 1.00 | Anticholinergic side effects (e.g. constipation, dry mouth); impaired cognitive performance;  electrocardiographic changes (prolonged QT) |  | Non-sedating, non-anticholinergic antihistamines^f^ like loratadine, cetirizine, but not terfenadine (which is PIM). *E* |
| R06AB05 | Pheniramine  In lists: 1 (A); 6 (B) | 15; 1.40 [1.12-1.68]; 1.00 | No proven efficacy; muscarinic-blocking agents; higher risk of confusion, sedation |  | Non-sedating, non-anticholinergic antihistamines^f^ like loratadine, cetirizine, but not terfenadine (which is PIM). *E* |
| R06AB52 | Dexchlorpheniramine-Betamethason  In lists: 1, 5 (A); 5, 6 (B) | 16; 1.31 [0.99-1.63]; 1.00 | Muscarinic-blocking drug; higher risk of sedation, drowsiness |  | Non-sedating, non-anticholinergic antihistamines^f^ like loratadine, cetirizine, but not terfenadine (which is PIM). *E* |
| R06AC04 | Tripelennamine  In lists: 6 (B) | 16; 1.75 [1.22-2.28]; 1.00 | Anticholinergic side effects (e.g. confusion, sedation) |  | Non-sedating, non-anticholinergic antihistamines^f^ like loratadine, cetirizine, but not terfenadine (which is PIM). *E* |
| R06AD01 | Alimemazine  In lists: 1 (A); 6 (B) | 13; 1.31 [1.02-1.60]; 1.00 | Muscarinic-blocking drug; higher risk of sedation, drowsiness | Reduce dose; start low–go slow. *M* | Non-sedating, non-anticholinergic antihistamines^f^ like loratadine, cetirizine, but not terfenadine (which is PIM). *E* |
| R06AD02 | Promethazine  In lists: 1, 5 (A); 5, 6 (B) | 18; 1.44 [1.14-1.75]; 1.00 | Anticholinergic side effects (e.g. confusion, sedation) | Reduce dose; start low–go slow. *M* Reduce starting dose to 6.25-12.5 mg for iv injection. *M* | Non-sedating, non-anticholinergic antihistamines^f^ like loratadine, cetirizine, but not terfenadine (which is PIM). *E* If used for insomnia: non-pharmacological treatment, passiflora, mirtazapine^d^, trazodone. *E* Consider low doses of short to intermediate benzodiazepines such as lormetazepam (≤0.5 mg/d), brotizolam (≤0.125 mg/d); zolpidem (≤5 mg/d), zopiclon (≤3.75 mg/d), zaleplon (≤5 mg/d) (suggested aternatives to hypnotic/sedative drugs) |
| R06AD07 | Mequitazine  In lists: 1 (A); 6 (B) | 12; 1.33 [0.92-1.75]; 1.00 | Anticholinergic side effects (e.g. confusion, sedation) |  | Non-sedating, non-anticholinergic antihistamines^f^ like loratadine, cetirizine, but not terfenadine (which is PIM). *E* |
| R06AD08 | Oxomemazine  In lists: 1 (A); 6 (B) | 11; 1.36 [0.91-1.82]; 1.00 | No proven efficacy; muscarinic-blocking agents; higher risk of confusion, sedation |  | Non-sedating, non-anticholinergic antihistamines^f^ like loratadine, cetirizine, but not terfenadine (which is PIM). *E* |
| R06AE01 | Buclizine  In lists: 1 (A); 6 (B) | 12; 1.33 [0.92-1.75]; 1.00 | No proven efficacy; muscarinic-blocking agents; higher risk of confusion, sedation |  | Non-sedating, non-anticholinergic antihistamines^f^ like loratadine, cetirizine, but not terfenadine (which is PIM). *E* |
| R06AE03 | Cyclizine  In lists: 3 (A); 6 (B) | 17; 1.53 [1.21-1.85]; 1.00 | No proven efficacy; muscarinic-blocking agents; higher risk of confusion, sedation |  | Non-sedating, non-anticholinergic antihistamines^f^ like loratadine, cetirizine, but not terfenadine (which is PIM). *E* |
| R06AE05 | Meclozine  In lists: 1, 3 (A); 6 (B) | 16; 1.44 [1.05-1.83]; 1.00 | No proven efficacy; muscarinic-blocking agents; higher risk of confusion, sedation |  | Non-sedating, non-anticholinergic antihistamines^f^ like loratadine, cetirizine, but not terfenadine (which is PIM). *E* |
| R06AX02 | Cyproheptadine  In lists: 1, 5 (A); 5, 6 (B) | 18; 1.28 [0.99-1.56]; 1.00 | Anticholinergic side effects (e.g. confusion, sedation) |  | Non-sedating, non-anticholinergic antihistamines^f^ like loratadine, cetirizine, but not terfenadine (which is PIM). *E* |
| R06AX07 | Triprolidine  In lists: 1, 4, 5 (A); 5, 6 (B) | 14; 1.43 [0.99-1.87]; 1.00 | Anticholinergic side effects (e.g. constipation, dry mouth); impaired cognitive performance;  electrocardiographic changes (prolonged QT) |  | Non-sedating, non-anticholinergic antihistamines^f^ like loratadine, cetirizine, but not terfenadine (which is PIM). *E* |
| R06AX12 | Terfenadine  In lists: does not appear as PIM | 17; 1.88 [1.52-2.24]; 2.00 | Adverse effects include prolonged QT interval, tachyarrhythmia, weakness, anxiety, agitation | Administer one tablet daily if CrCl <40 ml/min. *M* | Non-sedating, non-anticholinergic antihistamines^f^ like loratadine, cetirizine, but not terfenadine (which is PIM). *E* |
| R06AX22 | Ebastine  In lists: does not appear as PIM | 19; 2.26 [1.84-2.68]; 2.00 | Adverse events include impaired psychomotor performance with 50 mg or greater, somnolence, tachycardia, fatigue | Avoid / reduce dose if severe renal failure. *M* | Non-sedating, non-anticholinergic antihistamines^f^ like loratadine, cetirizine, but not terfenadine (which is PIM). *E* |
| R06AX23 | Pimethixene  In lists: 1 (A); 6 (B) | 11; 1.36 [0.91-1.82]; 1.00 | No proven efficacy; muscarinic-blocking agents; higher risk of confusion, sedation |  | Non-sedating, non-anticholinergic antihistamines^f^ like loratadine, cetirizine, but not terfenadine (which is PIM). *E* |
| N05BB01 | Hydroxyzine  In lists: 1, 3, 4, 5 (A); 5 (B) | 20; 1.40 [1.12-1.68]; 1.00 | Anticholinergic side effects (e.g. constipation, dry mouth); impaired cognitive performance, confusion, sedation; electrocardiographic changes (prolonged QT) | Reduce dose to at least 50% less than dose used for healthy younger adults. *E, M* | Non-sedating, non-anticholinergic antihistamines^f^ like loratadine, cetirizine, but not terfenadine (which is PIM). *E* Alternative therapies depending on indication. *E* |
| ^a^Category A (A): precisely this active substance is named as a PIM. Category B (B): i) this active substance is characterized as a PIM only in the case of certain clinical conditions or comorbidities or ii) this active substance is not specifically named but considered as a PIM drug class (e.g. anticholinergics or long-acting benzodiazepines). ^b^Decisive Delphi round: Delphi round in which consensus was reached (1st Delphi round: 26 experts participated; 2nd Delphi round: 24 experts participated; these numbers comprise two groups of 2 and 3 experts, respectively, doing joint assessments). ^c^Drug reevaluated during the last brief survey. ^d^Caution, this drug was judged to be questionable PIM. ^e^The following drugs belonging to this medication group were judged to be questionable PIM: citalopram, sertraline, escitalopram. ^f^In the group of non-sedating antihistamines, only loratadine was evaluated and judged to be questionable PIM; other drugs such as cetirizine were not evaluated. ^g^ATC according to WIDO (2013) [46]; ^h^ATC according to WHO ATC-code website 2013; ^i^ATC according to WHO ATC-code website 2014.  *E*: Experts; *M*: Micromedex^®^ [32]; *P*: PRISCUS list [22]; *L*: Laroche et al (2007) [3]; *McL*: McLeod et al (1997) [26]; *B*: Beers list (2012) [18]. ACE: Angiotensin-Converting-Enzyme; ADHD: Attention Deficit Hyperactivity Disorder; CNS: Central Nervous System; ECG: Electrocardiographic; GI: Gastrointestinal; PIM: Potentially Inappropriate Medication; PPI: Proton-Pump Inhibitors; SIADH: Syndrome of Inappropriate Antidiuretic Hormone secretion. Dosing abbreviations: CrCl: Creatinine Clearance; d: day; GFR: Glomerular Filtration Rate; iv: intravenous; mcg: micrograms; mg: milligram; min: minute; mL: millilitre; q: every.  Note: if nothing is stated under “Dose adjustment / special considerations of use”, this means that no suggestion was made either by the experts or in Micromedex^®^. | | | | | |

The EU(7)-PIM list: a list of potentially inappropriate medications for older people consented by experts from seven European countries. European Journal of Clinical Pharmacology. Anna Renom-Guiteras*, Gabriele Meyer, Petra A Thürmann. *Corresponding author: Faculty of Health, Institute of General Medicine and Family Medicine, University of Witten/Herdecke. Alfred-Herrhausen-Straße 50, 58448 Witten, Germany. [Anna.Renom@uni-wh.de](mailto:Anna.Renom@uni-wh.de).
