# Supplementary material for: The EU(7)-PIM list: a list of potentially inappropriate medications for older people consented by experts from seven European countries
Source: Eur J Clin Pharmacol. 2015 May 14;71(7):861–75. doi: 10.1007/s00228-015-1860-9 (PMC4464049; doi:10.1007/s00228-015-1860-9)
Supplement: Supplementary file 2 — (DOCX 25.9 kb) [file 228_2015_1860_MOESM2_ESM.docx]

Appendix 2: Questionable Potentially Inappropriate Medications (Questionable PIM): results of the Delphi survey.

| **Drug ATC^a^** | **Questionable PIM**  (number of experts’ answers at decisive Delphi round^b^) | **Results of the 5-point Likert scale** | |
| --- | --- | --- | --- |
|  |  | **Median** | **Mean [95% confidence interval]** |
| **A** | **Alimentary tract and metabolism** |  |  |
| ***A06*** | ***Laxatives*** |  |  |
| ***A06A*** | ***Laxatives*** |  |  |
| A06AC01 | Plantago ovate (=Ispaghula, =Psylla seed) (17) | 3 | 2.82 [2.27 - 3.38] |
| ***A10*** | ***Drug used in Diabetes*** |  |  |
| ***A10B*** | ***Blood glucose lowering drugs, excl. insulins*** |  |  |
| A10BA02 | Metformin (>2 x 850 mg/d) (21) | 2 | 2.57 [2.10 - 3.04] |
| **B** | **Blood and blood forming organs** |  |  |
| ***B01*** | ***Antithrombotic agents*** |  |  |
| ***B01A*** | ***Antithrombotic agents*** |  |  |
| B01AC06 | Aspirin low dose in primary prevention of cardiovascular disease (21) | 2 | 2.71 [2.23 - 3.19] |
| **C** | **Cardiovascular system** |  |  |
| ***C07*** | ***Beta-blocking agents*** |  |  |
| ***C07A*** | ***Beta-blocking agents*** |  |  |
| C07AG02 | Carvedilol (21) | 3 | 3.00 [2.50 - 3.50] |
| ***C08*** | ***Calcium channel blockers*** |  |  |
| ***C08C*** | ***Selective calcium channel blockers with mainly vascular effects*** |  |  |
| C08CA01 | Amlodipine (21) | 3 | 3.33 [2.85 - 3.82] |
| C08CA02 | Felodipine (18) | 3 | 2.78 [2.22 - 3.33] |
| **G** | **Genito urinary system and sex hormones** |  |  |
| ***G04*** | ***Urologicals*** |  |  |
| ***G04C*** | ***Drug used in benign prostatic hypertrophy*** |  |  |
| G04CA02 | Tamsulosin (19) | 3 | 3.00 [2.55 - 3.45] |
| **J** | **Anti-infectives for systematic use** |  |  |
| ***J01*** | ***Antibacterial for systemic use*** |  |  |
| ***J01M*** | ***Quinolone antibacterials*** |  |  |
| J01MA02 | Ciprofloxacin (21) | 3 | 3.29 [2.83 - 3.74] |
| J01MA12 | Levofloxacin (20) | 3.5 | 3.20 [2.73 - 3.67] |
| **N** | **Nervous system** |  |  |
| ***N02*** | ***Analgesics*** |  |  |
| ***N02A*** | ***Opioids*** |  |  |
| N02AA01 | Morphine sulfate (non-sustained-release) (21) | 3 | 3.33 [2.89 - 3.77] |
| ***N02B*** | ***Other analgesics and antipyretics*** |  |  |
| N02BB02 | Metamizole (16) | 1.5 | 2.25 [1.14 - 3.09] |
| ***N03*** | ***Antiepileptics*** |  |  |
| ***N03A*** | ***Antiepileptics*** |  |  |
| N03AF02 | Oxcarbazepine (20) | 2 | 2.65 [2.12 - 3.18] |
| N03AG01 | Valproic acid (20) | 2.5 | 2.95 [2.48 - 3.42] |
| N03AX09 | Lamotrigine (19) | 3 | 2.84 [2.35 - 3.33] |
| N03AX12 | Gabapentin (21) | 3 | 2.95 [2.53 - 3.37] |
| N03AX14 | Levetiracetam (18) | 4 | 3.17 [2.59 - 3.74] |
| N03AX15 | Zonisamide (11) | 2 | 1.82 [1.16 - 2.48] |
| N03AX16 | Pregabalin (21) | 2 | 2.81 [2.36 - 3.26] |
| ***N04*** | ***Antiparkinson drugs*** |  |  |
| ***N04B*** | ***Dopaminergic agents*** |  |  |
| N04BX01 | Tolcapone (15) | 2 | 2.60 [1.94 - 3.26] |
| N04BX02 | Entacapone (16) | 2.5 | 2.81 [2.22 - 3.40] |
| ***N05*** | ***Psycholeptics*** |  |  |
| ***N05A*** | ***Antipsychotics*** |  |  |
| N05AH04 | Quetiapine (18) | 2 | 2.67 [2.10 - 3.23] |
| ***N06*** | ***Psychoanaleptics*** |  |  |
| ***N06A*** | ***Antidepressants*** |  |  |
| N06AB04 | Citalopram (21) | 3 | 2.95 [2.51 - 3.40] |
| N06AB06 | Sertraline (21) | 3 | 2.95 [2.53 - 3.37] |
| N06AB10 | Escitalopram (21) | 3 | 2.86 [2.42 - 3.30] |
| N06AX11 | Mirtazapine (21) | 2 | 2.62 [2.20 - 3.04] |
| ***N06D*** | ***Anti-dementia drugs*** |  |  |
| N06DX01 | Memantine (20) | 3 | 3.15 [2.54 - 3.76] |
| **R** | **Respiratory system** |  |  |
| ***R03*** | ***Drugs for obstructive airway diseases*** |  |  |
| ***R03B*** | ***Other drugs for obstructive airway diseases, inhalants*** |  |  |
| R03BB01 | Ipratropium bromide (inhaled) (21) | 3 | 2.81 [2.34 - 3.28] |
| R03BB04 | Tiotropium bromide (inhaled) (20) | 2 | 2.70 [2.17 - 3.23] |
| ***R06*** | ***Antihistamines for systemic use*** |  |  |
| ***R06A*** | ***Antihistamines for systemic use*** |  |  |
| R06AX13 | Loratadine (19) | 3 | 2.74 [2.32 - 3.16] |
| ^a^According to WHO ATC-code list 2011 [30]; ^b^Decisive Delphi round: Delphi round in which the results presented were obtained (1st Delphi round: 26 experts participated; 2nd Delphi round: 24 experts participated; these numbers comprise two groups of 2 and 3 experts, respectively, doing joint assessments). | | | |

The EU(7)-PIM list: a list of potentially inappropriate medications for older people consented by experts from seven European countries. European Journal of Clinical Pharmacology. Anna Renom-Guiteras*, Gabriele Meyer, Petra A Thürmann. *Corresponding author: Faculty of Health, Institute of General Medicine and Family Medicine, University of Witten/Herdecke. Alfred-Herrhausen-Straße 50, 58448 Witten, Germany. [Anna.Renom@uni-wh.de](mailto:Anna.Renom@uni-wh.de).
