# Supplementary material for: The EU(7)-PIM list: a list of potentially inappropriate medications for older people consented by experts from seven European countries
Source: Eur J Clin Pharmacol. 2015 May 14;71(7):861–75. doi: 10.1007/s00228-015-1860-9 (PMC4464049; doi:10.1007/s00228-015-1860-9)
Supplement: Supplementary file 3 — (DOCX 19 kb) [file 228_2015_1860_MOESM3_ESM.docx]

Appendix 3: Non Potentially Inappropriate Medications (Non-PIM): results of the Delphi survey.

| **Drug ATC^a^** | **Non-potentially inappropriate drugs**  (number of experts’ answers at decisive Delphi round^b^) | **Results of the 5-point Likert scale** | |
| --- | --- | --- | --- |
|  |  | **Median** | **Mean [95% confidence interval]** |
| **A** | **Alimentary tract and metabolism** |  |  |
| ***A06*** | ***Laxatives*** |  |  |
| ***A06A*** | ***Laxatives*** |  |  |
| A06AD15 | Macrogol (2) | 3.5 | 3.45 [3.03 - 3.87] |
| A06AD11 | Lactulose (21) | 4 | 3.71 [3.36 - 4.07] |
| **B** | **Blood and blood forming organs** |  |  |
| ***B01*** | ***Antithrombotic agents*** |  |  |
| ***B01A*** | ***Antithrombotic agents*** |  |  |
| B01AC04 | Clopidogrel (23) | 4 | 3.74 [3.23 - 4.25] |
| ^a^According to WHO ATC-code list 2011 [30]; ^b^Decisive Delphi round: Delphi round in which the results presented were obtained (1st Delphi round: 26 experts participated; 2nd Delphi round: 24 experts participated; these numbers comprise two groups of 2 and 3 experts, respectively, doing joint assessments). | | | |

The EU(7)-PIM list: a list of potentially inappropriate medications for older people consented by experts from seven European countries. European Journal of Clinical Pharmacology. Anna Renom-Guiteras*, Gabriele Meyer, Petra A Thürmann. *Corresponding author: Faculty of Health, Institute of General Medicine and Family Medicine, University of Witten/Herdecke. Alfred-Herrhausen-Straße 50, 58448 Witten, Germany. [Anna.Renom@uni-wh.de](mailto:Anna.Renom@uni-wh.de).
